# Sequential versus concomitant treatment of androgen receptor signaling inhibitors and docetaxel for metastatic hormone-sensitive prostate cancer: an network meta-analysis

**DOI:** 10.3389/fphar.2024.1462360

**Published:** 2024-10-28

**Authors:** Chun Xing Li, Cong Ying Li, Yu Qiao Wang, Hua Liu, Zhan Jiang Yang, Xian Zhang, Guan Chun Wang, Lei Wang

**Affiliations:** ^1^ Department of Pharmacy, Aerospace Center Hospital, Peking University Aerospace School of Clinical Medicine, Beijing, China; ^2^ Beijing Rehabilitation Hospital, Capital Medical University, Beijing, China; ^3^ Department of Pharmacy, the 305th Hospital of PLA, Beijing, China; ^4^ Department of Urology, Peking University Shougang Hospital, Beijing, China; ^5^ Peking University Wu-jieping Urology Center, Peking University Health Science Center, Beijing, China

**Keywords:** prostate cancer, hormonal therapy, androgen receptor signaling inhibitors, sequential treatment, combination treatment ∗ overall survival, radiographic progression-free survival

## Abstract

**Background:**

Androgen receptor signaling inhibitors (ARSis), when administered sequentially or in combination with docetaxel and androgen deprivation therapy (ADT), have been shown to enhance overall survival (OS) and progression-free survival (PFS) in patients with metastatic hormone-sensitive prostate cancer (mHSPC). Nonetheless, the optimal sequence for administering chemotherapy and ARSis remains to be determined.

**Objective:**

To compare the efficacy of ARSis sequential therapy with ARSis combined therapy for mHSPC, and to evaluate the efficacy and safety of different combination regimens.

**Methods:**

The PubMed, Embase, Cochrane Central, and ClinicalTrials.gov databases were searched from their inception through 14 July 2024, to identify eligible phase III randomized clinical trials (RCTs) evaluating the combination or sequential use of docetaxel + ADT with abiraterone, enzalutamide, apalutamide, or darolutamide. The outcomes of interest included OS, PFS, time to prostate-specific antigen (PSA) progression, grade 3–5 adverse events (AEs), and serious adverse events (SAEs).

**Results:**

Five RCTs involving 2836 patients were included in the analysis. When comparing ARSis sequential therapy to ARSis combined therapy, no significant differences were observed in OS (Hazard Ratio (HR): 1.17, 95% Confidence Interval (CI): 0.69–1.96), PFS (HR: 1.03, 95% CI: 0.47–2.22), or time to PSA progression (HR: 0.48, 95% CI: 0.03–7.69). Within the different ARSis combined regimens, the triple therapies involving enzalutamide, abiraterone, and darolutamide demonstrated comparable efficacy and safety profiles in the overall population, and their efficacy in patients with high-volume disease or low-volume disease was also similar.

**Conclusion:**

ARSis sequential therapy did not significantly differ from ARSis combined therapy in improving OS and PFS among patients with mHSPC, and thus can be considered as a viable treatment option.

## 1 Introduction

Prostate cancer (PC) is the most prevalent malignant tumor among males, with 1.4 million new cases annually; moreover, around 20% of these cases involve metastatic hormone-sensitive prostate cancer (mHSPC) (Sung et al., 2021; Tilki et al, 2024). Androgen deprivation therapy (ADT) refers to the surgical removal of the testicles or the use of medication to regulate and reduce the production of androgens within the patient’s body. For mHSPC, ADT has historically been the first-line therapy and the only systematic treatment option (Chi et al., 2019; Mohler et al., 2019; Kinsey et al., 2020). Due to ongoing research and the development of new medications, the standard of care (SOC) for mHSPC has evolved from simple ADT or ADT combined with first-generation antiandrogens, such as bicalutamide and flutamide, to ADT plus docetaxel (Sweeney et al., 2015; Wang et al., 2023) or ADT plus novel hormonal agents, also known as androgen receptor signaling inhibitors (ARSis), including abiraterone, enzalutamide, apalutamide, darolutamide, etc. (Fizazi et al., 2022; Smith et al., 2022; Davis et al., 2019; Armstrong et al., 2019; Sartor and de Bono, 2018).

The EAU Guidelines 2024 (Tilki et al., 2024) recommend the triple therapy of ADT + docetaxel + ARSis based on the PEACE-1 (abiraterone) (Fizazi et al., 2022) and ARASENS (darolutamide) (Smith et al., 2022) studies, which shows better survival benefits compared to ADT + docetaxel (Ciccarese et al., 2022; James et al., 2016; Yanagisawa et al., 2022; Menges et al., 2022; Wang et al., 2021). In ARASENS (Smith et al., 2022), all patients received docetaxel in combination with ADT as the established SOC. In PEACE-1 (Fizazi et al., 2022), ADT was initially considered to be SOC from November 2013, then docetaxel was permitted at the researcher’s discretion since October 2015, and became a mandatory component of SOC from August 2017. Given that ARSi and docetaxel were administered simultaneously, these two studies represent ARSis combination therapy. In the ENZAMET trial (Davis et al., 2019), the majority of patients (around 65%) did not initiate docetaxel chemotherapy before randomization and were instructed to begin docetaxel approximately 4–6 weeks post-randomization, which is comparable to initiating enzalutamide. A minority of patients (approximately 35%) who had already undergone one to two cycles of docetaxel prior to randomization were still eligible for enrollment and subsequently received the remaining four to five cycles of docetaxel in conjunction with enzalutamide. Therefore, the study was also regarded as evaluating ARSis combination therapy.

ARSis sequential therapy refers to the administration of ARSis therapy following the use of docetaxel while ADT is ongoing. It also serves as an alternative treatment approach for patients with mHSPC. In both TITAN (Chi et al., 2019) and ARCHES (Armstrong et al., 2019), up to six courses of prior docetaxel chemotherapy were permitted, and the final treatment administration had to be completed within 2 months prior to randomization. The two studies, in which 10.7% and 17.8% of the enrolled patients had previously received docetaxel, respectively, were considered as ARSis sequential therapy. Subgroup analyses from the TITAN (Chi et al., 2019) and ARCHES (Armstrong et al., 2019) trials indicated that sequencing ARSis after ADT plus docetaxel demonstrated a trend towards OS benefit compared to ADT plus docetaxel alone (Chi et al., 2021; Armstrong et al., 2022).

Compared with ARSis combined therapy, sequential therapy may have more advantages in drug safety, reducing expenditure, saving medical resources and improving compliance, and it also aligns with the medication philosophy of some physicians. However, previous studies did not specifically study sequential therapy in mHSPC patients, and the conclusions from the subgroup analyses are not clear (Mandel et al., 2023; Chen et al., 2020; Dou et al., 2023; Hussain et al., 2023). Neither the NCCN nor the EAU guidelines provide pertinent recommendations. Addressing this matter should facilitate the provision of more personalized and evidence-based treatment options and optimizations in clinical practice. Therefore, the current study aimed to compare the efficacy of ARSis sequential therapy against that of combined therapy through a systematic literature review and network meta-analysis (NMA). The findings will inform the medication regimens for mHSPC patients.

## 2 Methods

This NMA was performed in accordance with the guidelines of the Preferred Reporting Items for Systematic Reviews and Meta-analyses (PRISMA) extension statement (Page et al., 2021; Hutton et al., 2015), and it was registered in the International Prospective Register of Systematic Reviews database (PROSPERO: CRD42022363656).

### 2.1 Search strategy

We conducted a comprehensive search of the PubMed, Embase, Cochrane Central Register of Randomized Controlled Trials (CENTRAL), and ClinicalTrials.gov databases up to 20 October 2022, to identify eligible randomized controlled trials (RCTs). The search was subsequently updated in July 2024. In addition, we scrutinized proceedings from key conferences, including the American Society of Clinical Oncology and the European Society for Medical Oncology. Two authors (Li CY and Wang YQ) independently screened the literature based on predefined inclusion and exclusion criteria, which were formulated in line with the PICOS principles (participants, intervention, comparison, outcomes, and study characteristics, and study design). Any discrepancies were resolved through discussions with a third author (Li CX). The specific search strategies are detailed in Supplementary Material 1.

### 2.2 Inclusion and exclusion criteria

The inclusion criteria were as follows: 1) mHSPC patients aged ≥18 years; 2) patients with initial onset or those who progressed after previous local therapy; and 3) patients with a duration of ADT treatment in the localized prostate cancer stage no longer than 3 years or a duration of ADT in the metastatic prostate cancer stage no longer than 6 months. The following interventions were eligible: ADT + docetaxel + ARSis sequential therapy or ADT + docetaxel + ARSis combined therapy. Sequential therapy was defined as the initial treatment of ADT + 6-week cycles of docetaxel chemotherapy followed by ARSis treatment after discontinuation of docetaxel. Combined therapy was defined as the combination of ADT + docetaxel with ARSis. ARSis included abiraterone, enzalutamide, apalutamide and darolutamide. The outcomes of interest were overall survival (OS), radiographic progression-free survival (rPFS), clinical progression-free survival (cPFS), time to prostate-specific antigen (PSA) progression and safety indicators of grade 3–5 adverse events (AEs) and serious adverse events (SAEs). The exclusion criteria were as follows: 1) patients did not have mHSPC; 2) studies that did not have any relevant or original data; 3) non-RCTs such as letters and case reports; 4) duplicate studies; and 5) non-English articles.

### 2.3 Data extraction

The data were extracted according to the PRISMA guidelines. The following data were extracted: the first author’s name, publication date, participant characteristics, inclusion and exclusion criteria, number of cases in each group, interventions, follow-up duration, hazard ratios (HRs) of outcomes, 95% confidence intervals (CIs), and number of AEs. When multiple papers reported results from the same study at different stages, only the most recent results were considered.

### 2.4 Risk of bias assessment

A risk of bias assessment was carried out by two authors independently using the revised Cochrane Collaboration Risk of Bias Tool (RoB) 2.0 (Sterne et al., 2019). Any disagreements were resolved via discussion with a third author. The risk of bias was categorized as “low risk of bias”, “some concerns” and “high risk of bias”. Each study was evaluated on the following domains: selection bias, performance bias, and detection bias. If all aspects were deemed to have a low risk of bias, the entire study was considered to have a low risk of bias. Conversely, if any aspect was determined to have a high risk of bias, the whole study was classified as having a high risk of bias; all other situations were categorized as having a moderate risk of bias.

### 2.5 Data synthesis strategy

Three similar indicators related to PFS were used in different studies, including rPFS (Armstrong et al., 2019; James et al., 2016; Menges et al., 2022; Wang et al., 2021; Mandel et al., 2023; Hutton et al., 2015; HHoyle et al., 2019; Sydes et al., 2018; Mori et al., 2022) and cPFS (Davis et al., 2019; Armstrong et al., 2019; Yanagisawa et al., 2022). Since the three were similar in definition and in most studies, imaging progression occurred earlier than the aggravation of clinical symptoms and death, we unified the three into one “generalized PFS” in the current meta-analysis (James et al., 2016). Subgroup analysis was performed according to tumor burden (high-volume versus low-volume disease). In accordance with the CHAARTED study criteria (Armstrong et al., 2019; Mandel et al., 2023; Jian et al., 2022), high-volume disease (HVD) was defined as the presence of visceral metastases and/or four or more bone metastases, with at least one bone metastasis located outside the spine and pelvis.

### 2.6 Statistical analysis

Our NMA was conducted using the “gemtc” and “rjags” packages of R 4.0.5 software, employing the Monte Carlo Markov Chain (McMc) method within a Bayesian models. Bayesian NMA is a statistical method used to combine the results of multiple independent studies to obtain more accurate estimates and inferences (Uhlmann et al., 2018). Forest plots and network diagrams were generated for each outcome. HRs and 95% CIs were calculated for each intervention by using the consistency model. The benefits of treatments were ranked using the Surface Under the Cumulative Ranking Curve (SUCRA). The SUCRA index spans from 0 (or 0%) to one (or 100%), with the treatment scoring the highest SUCRA value deemed the optimal choice, and the one with the lowest SUCRA value considered the least effective. The analysis employed four model chains, an initial value of 2.5, a sampling number of 5,000, and 50,000 iterations with a step size of 10. The “mtc.anohe” command in the “gemtc” package was utilized, and trace plotting and density plotting methods were employed to evaluate the model’s convergence (Shim et al., 2019; Rücker and Schwarzer, 2016; Woods et al., 2010). We extracted the “number of patients with grade 3–5 AEs/total patients” from each group and calculated the odds ratio (OR) and 95% CIs.

## 3 Results

### 3.1 Study selection and characteristics

**FIGURE 1:**
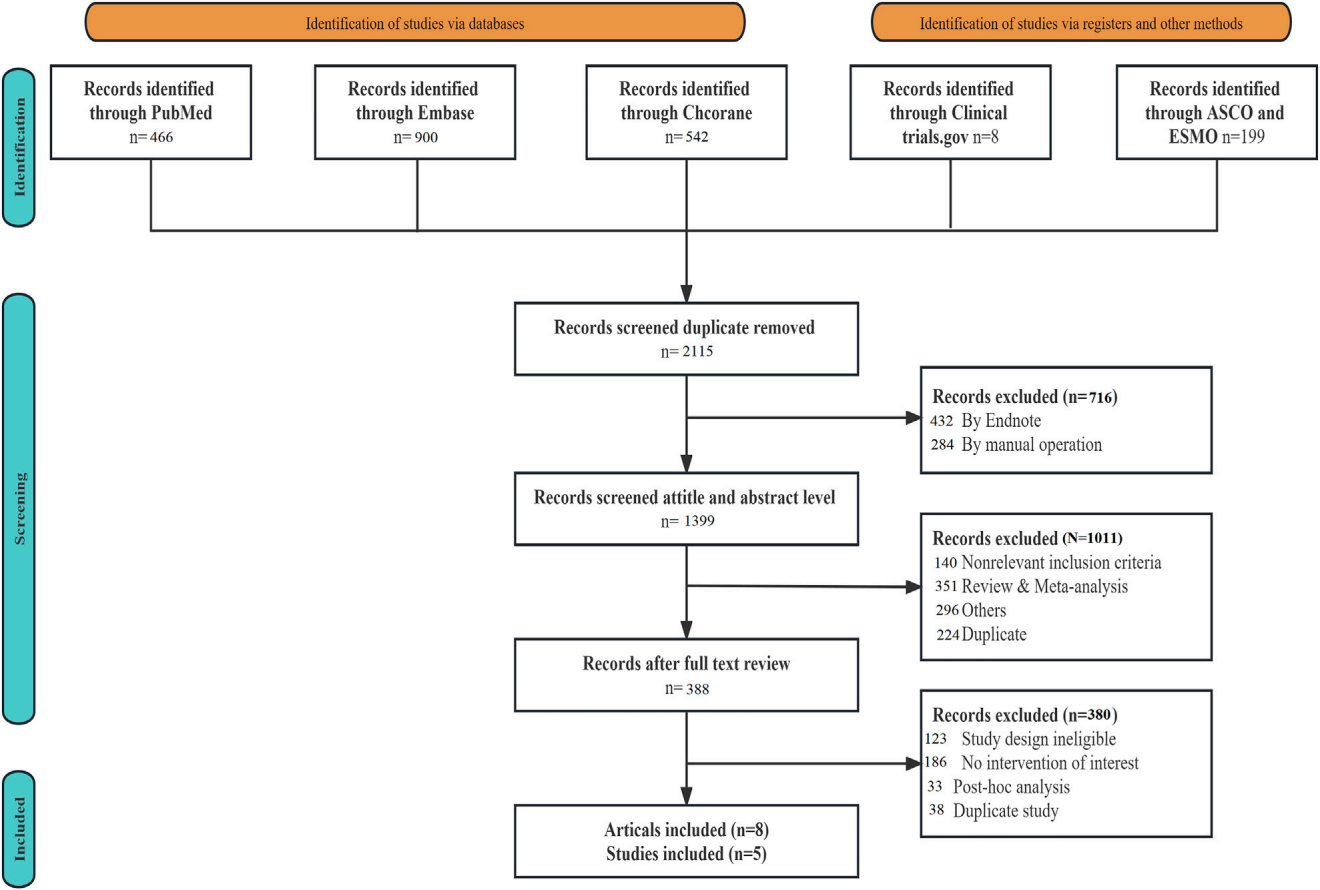
The flow chart of literature review screening.

A total of 2,115 articles were retrieved during the literature search. Following the removal of duplicate studies using EndNote 19.0, along with manual elimination, and conducting preliminary and fine screening based on the established inclusion and exclusion criteria, 2,107 articles were excluded. Ultimately, eight articles, comprising five RCTs (ENZAMET, PEACE-1, ARASENS, ARCHES, and TITAN), were included (Figure 1). Table 1 provides the basic information for each study. The number of patients in each study ranged from 113 to 1,305, with a median age spanning from 41 to 70 years. The median follow-up time varied between 34 and 83.9 months.

**TABLE 1:** Characteristics of the included studies.

	ARSi combined with DOC + ADT			ARSi sequential to DOC + ADT	
Trial	ARASENS (N = 1305)	PEACE-1 (N = 710)^a^ (Total pts: 1172)	ENZAMET (N = 503)^a^ (Total pts: 1125)	ARCHES (N = 205)^a^ (Total pts: 1150)	TITAN (N = 113)^a^ (Total pts: 1052)
First author	Smith MR	Fizazi K	Davis ID	Armstrong AJ	Chi KN
Year	2022	2022	2019	2019	2019
Experimental arm	Darolutamide + DOC + ADT	Abiraterone + DOC + ADT	Enzalutamide + DOC + ADT	Enzalutamide + DOC + ADT	Apalutamide + DOC + ADT
Control arm	DOC + ADT	DOC + ADT	DOC + ADT	DOC + ADT	DOC + ADT
The use of docetaxel	Six cycles of docetaxel were administered concurrently with ARSI after randomization	Six cycles of docetaxel were administered concurrently with ARSI. The first docetaxel cycle had to be administered within 14 days after randomisation	Docetaxel for a maximum of 6 cycles up to 2 cycles were permitted before randomization	Up to 6 cycles, with the last dose ≤2 months prior to randomization and with no evidence of progression during treatment or before randomization	Up to 6 cycles, with the last dose ≤2 months prior to randomization and with no evidence of progression during treatment or before randomization
Patients no. (Exp. vs Ctrl.)	651/654	355/355	254/249	103/102	58/55
Age, years (Exp.)	67 (range: 41–89)	67 (range: 37–94)	69.2 (IQR: 63.2–74.5)	67 (range: 46–84)	69 (range:45–94)
Age, years (Ctrl.)	67 (range: 42–86)	66 (range: 43–87)	69.0 (IQR: 63.6–74.5)	68 (range: 42–83)	68 (range:43–90)
Serum PSA level - ng/ml (Exp.)	Median (Range) 30.3 (0.0–9219.0)	Median (IQR) 14 (2–59)	NR	Median (Range) 0.8 (0.0–493.7)	Median (Range) 5.97 (0–2682)^b^
Serum PSA level - ng/ml (Ctrl.)	Median (Range) 24.2 (0.0–11,947.0)	Median (IQR) 12 (3–60)	NR	Median (Range) 0.76 (0.0–280.8)	Median (Range) 4.02 (0–2229)^b^
Gleason score at initial diagnosis -no. (%)(Exp.)	<8:122 (18.7) ≥8:505 (77.6)	<8:79 (23%) ≥8:270 (77%)	<8:27%^b^ ≥8:59.5%^b^	<8:23 (22.3%) ≥8:76 (73.8%)	<8:33.1%^b^ ≥8:66.9%^b^
Gleason score at initial diagnosis -no. (%)(Ctrl.)	<8:118 (18.0) ≥8:516 (78.9)	<8:71 (20%) ≥8:276 (80%)	<8:29%^b^ ≥8:57.1%^b^	<8:26 (25.5%) ≥8:72 (70.6%)	<8:32.1%^b^ ≥8:67.9%^b^
Metastasis stage at initial diagnosis -no. (%) (Exp.)	M1: 558 (85.7) M0: 86 (13.2)	M1: 100%	M1: 57.7%^b^ M0: 42.3%^b^	M1: 88 (88%) M0: 12 (12%)	M1: 78.3%^b^ M0: 16.2%^b^
Metastasis stage at initial diagnosis -no. (%) (Ctrl.)	M1: 566 (86.5) M0: 82 (12.5)	M1: 100%	M1: 58.2%^b^ M0: 41.8%^b^	M1: 85 (83.3%) M0: 17 (16.7%)	M1: 83.7%^b^ M0: 11.2%^b^
HVD vs LVD no. (Exp.)	497/154	224/131	177/77	73/30	NR
HVD vs LVD no. (Ctrl.)	508/146	232/123	179/70	72/30	NR
Completion of six docetaxel cycles (Exp. vs Ctrl.)	87.6% vs 85.5%	median 6 cycles in both arms (IQR 6–6)	65.4% vs 76.1%	86.4% vs 89.2%	median 6 cycles in both arms
	HR for OS (95% CI)				
All patients	0.68 (0.57–0.80)	0.75 (0.59–0.95)	0.90 (0.62–1.31)	0.74 (0.46–1.20)	1.12 (0.59–2.12)
HVD	0.69 (0.57–0.82)	0.72 (0.55–0.95)	0.97 (0.64–1.46)	NR	NR
LVD	0.68 (0.41–1.13)	0.83 (0.50–1.39)	0.65 (0.25–1.71)	NR	NR
	HR for rPFS✝ (95% CI)				
All patients	NR	0.50 (0.34–0.71)	0.48 (0.37–0.62)	0.52 (0.30–0.89)	NR
HVD	NR	0.47 (0.30–0.72)	0.51 (0.38–0.69)	NR	NR
LVD	NR	0.58 (0.29–1.15)	0.37 (0.20–0.67)	NR	NR

^a^Subgroup of patients who received docetaxel chemotherapy.✝clinical Progression-free survival (cPFS) in ENZAMET, study; ARSI: androgen receptor signaling inhibitors; ADT: androgen deprivation treatment; DOC: docetaxel; Exp.: experimental arm; Ctrl.: control arm; OS: overall survival; rPFS: radiographic progression-free survival; IQR: interquartile range; NR: not reported; HVD: patients with high volume disease; LVD: patients with low volume disease.

^b^Data from all patients in the trial, not only those with docetaxel use.

Three studies—ENZAMET, PEACE-1, and ARASENS—utilized a combination therapy involving ARSis, whereas the remaining two studies, TITAN and ARCHES, employed sequential therapy with ARSis. A total of 2,518 individuals were administered the combination therapy, and 318 underwent sequential therapy. The median age of patients in both treatment groups was comparable, as were their Gleason scores and metastatic stages at the time of initial diagnosis. The sequential therapy group exhibited lower serum PSA levels compared to the combination therapy group, and no subgroup analyses were conducted to assess the impact on patients with high and low tumor burdens within the sequential therapy group. (Table1, Supplementary Material 3).

### 3.2 Risk of bias assessment

The risk of bias for each study is provided in Supplementary Material 2. The included studies were phase III RCTs, and the risk of bias for all five trials was deemed to be low.

### 3.3 Overall survival (OS)

**FIGURE 2:**
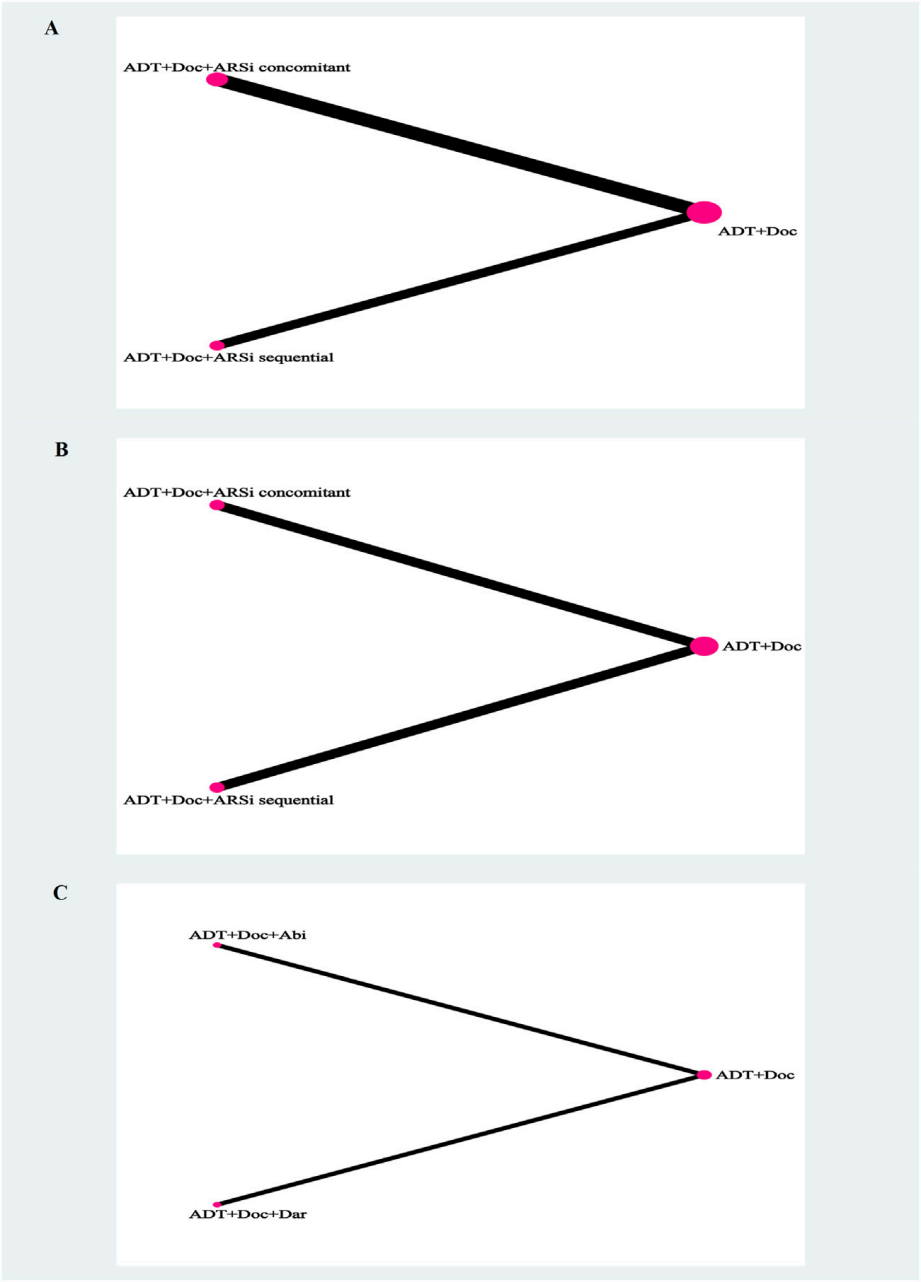
The network plot of network meta-analysis. Legend: overall survival **(A)**, generalized progression-free survival **(B)**, grade 3–5 adverse events **(C)**. Each circle indicates a treatment node. Lines connecting 2 nodes represent direct comparisons between 2 treatments. The size of the nodes is proportional to the number of trials evaluating each treatment. The thickness of the lines is proportional to the number of trials directly comparing the 2 connected treatments.

**FIGURE 3:**
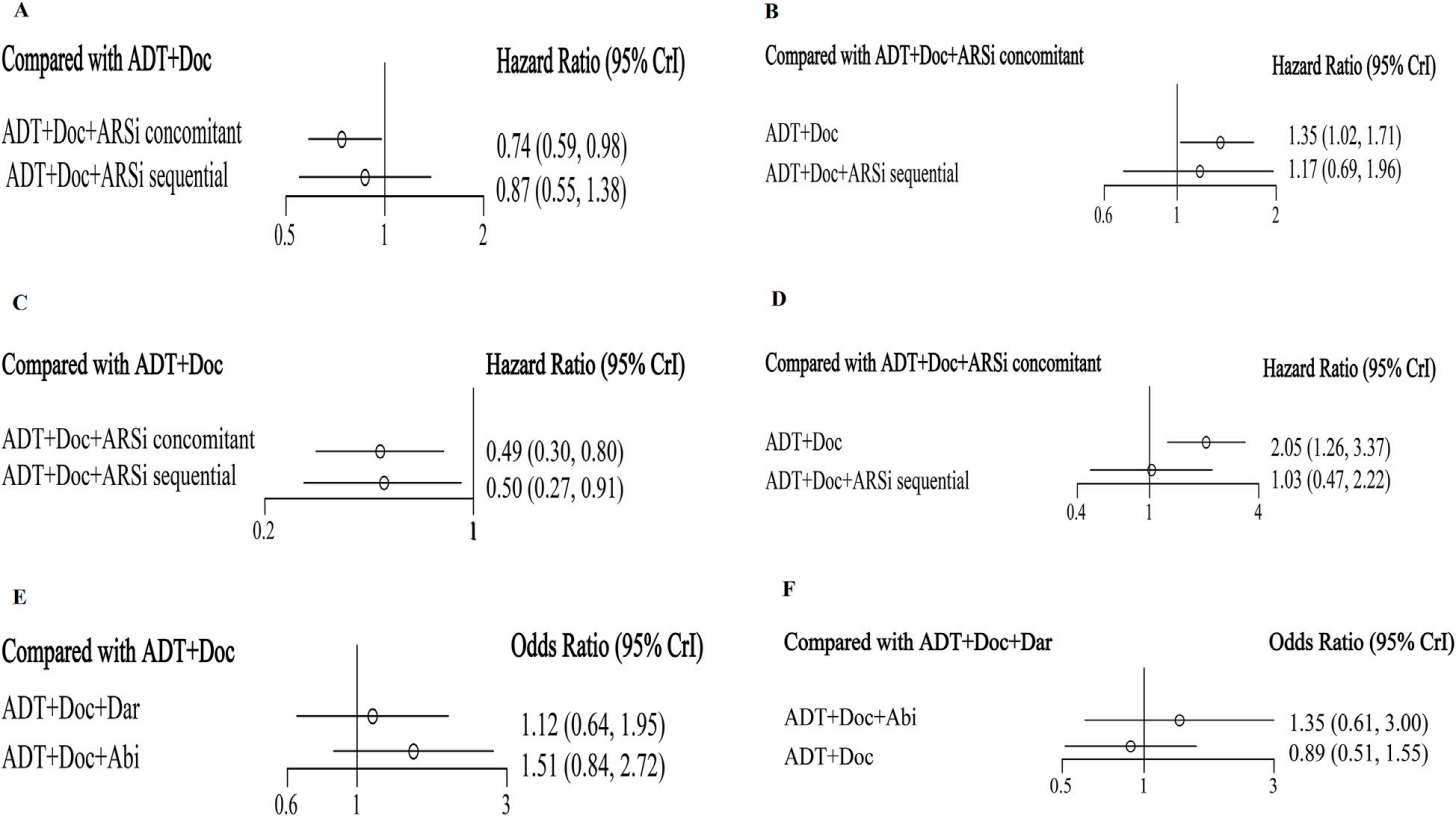
The forest plot of network meta-analysis. Legend: overall survival **(A, B)**, generalized progression-free survival **(C, D)**, grade 3–5 adverse events **(E, F)**.

All five studies analyzed OS (Supplementary Material 4). The network diagram depicting different interventions for mHSPC is presented in Figure 2A. The trace plot and density plot showed perfect model convergence (Supplementary Material 10). The results showed that compared with ADT + docetaxel, ARSis sequential therapy (HR: 0.87, 95% CI: 0.55–1.38) (Figure 3A) did not yield a statistically significant improvement in OS for mHSPC patients; ARSis combined therapy (HR: 0.74, 95% CI: 0.59–0.98) (Figure 3A) significantly improved OS in mHSPC patients. Furthermore, when ARSis sequential therapy was compared with ARSis combined therapy, no statistically significant difference was observed in OS (HR: 1.17, 95% CI: 0.69–1.96) (Figure 3B). ARSis combined therapy appeared to offer the most substantial improvement in OS, followed by ARSis sequential therapy, and lastly ADT + docetaxel therapy, based on their respective SUCRA values of 0.88, 0.50, and 0.14 (Supplementary Material 9A, 11A).

In the ARSis combined therapies, when compared with ADT + docetaxel, different ARSis exhibited comparable efficacy in improving OS for mHSPC patients (Supplementary Material 6A, B). In terms of rank probability, docetaxel + ADT + darolutamide had the highest probability of being ranked the best (SUCRA 0.79), followed by docetaxel + ADT + abiraterone (SUCRA 0.65) and docetaxel + ADT + enzalutamide (SUCRA 0.39) (Supplementary Material 9B, 11B). In ARSis sequential therapies, when compared with ADT + docetaxel, enzalutamide and apalutamide showed similar effects in improving OS for mHSPC patients (Supplementary Material 6C, D), with enzalutamide having the highest rank probability (SUCRA 0.83) (Supplementary Material 9C, 11C).

### 3.4 Generalized PFS

Excluding ARASENS, the remaining four studies reported rPFS or cPFS (Figure 2B). Two of these studies (ARCHES, TITAN) implemented ARSis sequential therapy, whereas the other two (PEACE-1, ENZAMET) utilized ARSis combined interventions. The findings showed that, in comparison with ADT + docetaxel, both ARSis sequential therapy (HR: 0.50, 95% CI: 0.27–0.91) and ARSis combined therapy (HR: 0.49, 95% CI: 0.30–0.80) significantly enhanced generalized PFS in mHSPC patients (Figure 3C). However, upon comparing ARSis sequential therapy with ARSis combined therapy, no statistically significant difference was found in terms of improving generalized PFS for mHSPC patients (HR: 1.03, 95% CI: 0.47–2.22) (Figure 3D). ARSis combined interventions appeared to offer the greatest improvement in generalized PFS, followed by ARSis sequential interventions and finally ADT + docetaxel according to their SUCRA values of 0.76, 0.73 and 0.01, respectively (Supplementary Material 9F, 11F).

In terms of ARSis combination therapy, different ARSis showed similar effects in improving generalized PFS (docetaxel + ADT + enzalutamide: HR: 0.48, 95% CI: 0.19–1.25; docetaxel + ADT + abiraterone: HR: 0.50, 95% CI: 0.19–1.32) (Supplementary Material 6J, K). In ARSis sequential therapy, enzalutamide and apalutamide showed similar effects in improving rPFS for mHSPC patients (docetaxel + ADT + apalutamide: HR: 0.52, 95% CI: 0.16–1.52; docetaxel + ADT + enzalutamide: HR: 0.48, 95% CI: 0.15–1.56) (Supplementary Material 6L, M).

### 3.5 Time to PSA progression

Regarding the time to PSA progression, two noteworthy studies (ARCHES and ENZAMET) were examined (Supplementary Material 5I). The results revealed that compared to docetaxel, enzalutamide sequential therapy (HR: 0.22, 95% CI: 0.03–1.67) and enzalutamide combined therapy (HR: 0.46, 95% CI: 0.06–3.07) exhibited a favorable trend in enhancing the time to PSA progression in mHSPC patients (Supplementary Material 6R). However, when comparing the two enzalutamide therapies, no statistically significant difference in improving the time to PSA progression was observed in mHSPC patients (Supplementary Material 6S, T).

### 3.6 Subgroup analysis

Sequential therapy lacks subgroup data on tumor burden, with only three studies on ARSis combination therapy reporting subgroup data on tumor burden; therefore, we only conducted a subgroup analysis on ARSis combination therapy. The results showed that in the HVD or LVD subgroups, there were no statistically significant differences in OS and PFS for various ARSis combination therapies (Supplementary Material 6).

### 3.7 Satety

Only two ARSis combined studies (PEACE-1 and ARASENS) conducted a detailed analysis of grade 3–5 AEs (Figure 2C). The results showed that there was no significant difference between abiraterone and darolutamide in grade 3–5 AEs (Figures 3E, F) or SAEs (Supplementary Material 6U, V).

Currently, no data comparing the safety of sequential therapy and combination therapy has been found in studies that meet the inclusion criteria. The discussion will mention the *post hoc* analyses of the ARCHES study, which analyzed the safety differences between sequential therapy with docetaxel + ADT + enzalutamide versus docetaxel + ADT versus ADT + enzalutamide.

## 4 Discussion

In recent years, the treatment regimen for patients with mHSPC has shifted from ADT monotherapy to a combination of ADT and docetaxel/abiraterone (ARSis) dual therapy. It is anticipated that this will further evolve into a triple therapy regimen, including ADT, docetaxel, and ARSis, for patients with HVD. The ongoing refinement of treatment protocols has significantly improved survival outcomes for mHSPC patients (Siegel et al., 2022; James et al., 2017; Armstrong et al., 2021). ARSis directly modulate androgen receptor signaling, whereas docetaxel indirectly targets androgen receptor translocation by suppressing microtubule protein polymerization (Hoyle et al., 2019; Tannock et al., 2004; Nader et al., 2018; Thadani-Mulero et al., 2012; Rizzo, 2021). Consequently, these two treatments can synergistically disrupt androgen receptor activity (Maiorano et al., 2022). Results from the PEACE-1 and ARASENS trials (Hoyle et al., 2019; Rizzo, 2021) have demonstrated that initiating ARSis in conjunction with docetaxel and ADT can enhance patient OS more effectively than dual therapy with ADT and docetaxel alone. Furthermore, the latest meta-analysis data (Mori et al., 2022; Jian et al., 2022; Thadani-Mulero et al., 2012) indicates that triple therapy leads the pack in terms of OS benefits.

However, the optimal sequence of chemotherapy and ARSis remains to be determined, despite some opinions suggesting that the efficacy of ARSis could be compromised if preceded by docetaxel (Rice et al., 2019). In clinical practice, the sequential use of ADT + docetaxel therapy, followed by ARSis, is commonly employed, primarily based on two considerations. 1) Currently, those who initially receive docetaxel chemotherapy are generally mHSPC patients with HVD. However, not all patients with HVD may require an aggressive treatment regimen that combines docetaxel and ARSis from the outset. 2) Initiating therapy with a more intense regimen can lead to more severe side effects and a greater financial burden for patients. In contrast, sequential therapy with ARSis following docetaxel is less harsh and has better patient compliance.

To evaluate the effectiveness of the aforementioned sequential therapy compared to combination therapy, and then provide a basis for treatment options, we undertook a NMA of ARSis administered concurrently with or following ADT + docetaxel for the management of mHSPC. This study encompassed a group of patients from the ARCHES and TITAN trials who had previously received ARSis post-docetaxel as the sequential therapy cohort, and patients from the ENZAMET, PEACE-1, and ARASENS studies who were treated with ARSis and docetaxel simultaneously as the combination therapy cohort.

Table 1 shows that compared to ADT + Doc, the HR value for sequential ADT + Doc + Apa is 1.12, which is greater than 1, indicating a trend towards a negative effect, but this is not statistically significant. This is a common phenomenon in research. Primarily, this can be attributed to two factors: 1) a small sample size (n = 58/55); 2) There are many factors affecting OS (such as tumor burden, etc.). Therefore, the comparison results still look forward to verification with larger sample size data.

To our knowledge, this study represents the first comparative analysis of the distinctions between sequential and combination treatment regimens. The findings indicated that there was no significant difference in OS, PFS, or time to PSA progression between sequential therapy and combination therapy. Although combination regimens are theoretically anticipated to outperform sequential regimens due to their increased intensity, this study observed minimal differences in generalized PFS (HR: 1.03, 95% CI: 0.47–2.22). Moreover, while combination regimens were ranked higher in terms of OS, the advantage over sequential therapy was not statistically significant (HR: 1.17, 95% CI: 0.69–1.96). These outcomes lend support to the adoption of sequential regimens for specific patient populations, such as mHSPC patients with HVD, who are relatively older, have multiple comorbidities, and are concerned about the side effects of treatment.

Due to the absence of stratified data on HVD and LVD among patients receiving sequential treatments, we compared the data for HVD and LVD subgroups across various combination regimens. The findings indicated that there was no significant difference in efficacy among triple therapies involving darolutamide, abiraterone, and enzalutamide. However, darolutamide triple therapy emerged as the most effective in terms of the ranking of curative effects.

The increased toxicity associated with intensive therapy can influence clinical decision-making greatly. Due to the limited safety data from subgroup studies, it was not possible to compare the safety differences between sequential therapy and combination therapy. In ARASENS and PEACE-1, the incidence of AEs were highest during the first 6 months of treatment, just the overlapping period when ARSis were administered in combination with docetaxel. In PEACE-1 and ENZAMET, grade 3–5 AEs were reported more frequently in experimental groups compared with that in the control groups. This suggests that the safety of ARSis in combination with docetaxel should be paid more attention to. Specifically, hypertension (6.4% vs 3.2%) was the only significantly elevated grade 3 to 4 adverse effect in ARASENS. Among patients receiving docetaxel in PEACE-1, moderate differences were seen in grade 3 or worse AEs for hypertension (22% vs 13%) and hepatotoxicity (6% vs 1%). For patients treated with docetaxel in ENZAMET, neutropenia was the only grade 3 or 4 adverse effect that occurred more frequently (Hussain et al., 2024).

Docetaxel is associated with high frequencies of AEs, but due to limited time of administration, the AEs will be significantly reduced and often well controlled after the chemotherapy cycles. At this time, sequential ARSis should have certain advantages from the safety point of view, especially for elderly and weak patients. In TITAN, there was no substantial difference in the safety profile of apalutamide between patients with or without prior docetaxel. Post hoc analyses results from the ARCHES study Azad et al., 2022 showed that the incidence of total adverse events (AEs) (70/103, 68% vs 254/469, 54.2%), fatigue (34/103, 33.0% vs 39/469, 8.3%), and hypertension (10/103, 9.7% vs 104/469, 22.2%) was slightly higher in the docetaxel + ADT + enzalutamide group compared to the ADT + enzalutamide group, and similar to that of docetaxel + ADT (total AEs 64/102, 62.7%; fatigue 30/102, 29.4%; hypertension 9/102, 8.8%), but there lacked data of grade 3–4 AEs. Future prospective head-to-head studies comparing the safety and efficacy of abiraterone/enzalutamide sequential or in combination with docetaxel will shed more light on these issues.

The limitations of this paper are as follows. 1) The sample size of the sequential treatment group is relatively small, which diminishes the statistical power. 2) The ENZAMET, ARCHES, and TITAN trials were not strictly designed in accordance with three-drug combination therapy or sequential therapy protocols. The data utilized in this paper are derived solely from the outcomes of subgroup analyses. The quality and reliability of these data should be scrutinized with care. 3) There is a deficiency in subgroup data for the sequential treatment group, notably a scarcity of further stratification for patients with HVD. To achieve more compelling results, larger-scale and specifically designed clinical trials are required. 4) The absence of data comparing the safety profiles of sequential therapy and combination therapy leaves a gap in our understanding. There is an expectation for research that addresses this safety aspect.

## 5 Conclusion

In summary, this study conducted a comprehensive review of triple therapy regimens for mHSPC. The findings indicated that the combination of ADT with docetaxel and an ARSi resulted in survival benefits over ADT plus docetaxel alone. Furthermore, sequential administration of an ARSi following ADT plus docetaxel also demonstrated survival advantages compared to ADT plus docetaxel alone. An indirect comparison of sequential therapy and combination therapy suggested that sequential therapy is not inferior in terms of efficacy and can be considered a viable treatment option in clinical practice, particularly for patients with HVD who are particularly concerned about treatment-related side effects.

## Data Availability

The original contributions presented in the study are included in the article/Supplementary Material, further inquiries can be directed to the corresponding authors.

## References

[B1] Armstrong A. J. Azad A. A. Iguchi T. Szmulewitz R. Z. Petrylak D. P. Holzbeierlein J. (2022). Improved survival with enzalutamide in patients with metastatic hormone-sensitive prostate cancer. J. Clin. Oncol. 40, 1616–1622. 10.1200/JCO.22.00193 35420921 PMC9113211

[B2] Armstrong A. J. Iguchi T. Azad A. A. Szmulewitz R. Z. Holzbeierlein J. Villers A. (2021). Final overall survival (OS) analysis from ARCHES: a phase III, randomized, double-blind, placebo (PBO)-controlled study of enzalutamide (ENZA) androgen deprivation therapy (ADT) in men with metastatic hormone-sensitive prostate cancer (mHSPC). Ann. Oncol. 32, S1283–S1346.

[B3] Armstrong A. J. Szmulewitz R. Z. Petrylak D. P. Holzbeierlein J. Villers A. Azad A. (2019). ARCHES: a randomized, phase III study of androgen deprivation therapy with enzalutamide or placebo in men with metastatic hormone-sensitive prostate cancer. J. Clin. Oncol. 37, 2974–2986. 10.1200/JCO.19.00799 31329516 PMC6839905

[B4] Azad A. A. Armstrong A. J. Alcaraz A. Szmulewitz R. Z. Petrylak D. P. Holzbeierlein J. (2022). Efficacy of enzalutamide in subgroups of men with metastatic hormone-sensitive prostate cancer based on prior therapy, disease volume, and risk. Prostate Cancer Prostatic Dis. 25 (2), 274–282. 10.1038/s41391-021-00436-y 34420037

[B5] Chen J. Ni Y. Sun G. Liao B. Zhang X. Zhao J. (2020). Comparison of current systemic combination therapies for metastatic hormone-sensitive prostate cancer and selection of candidates for optimal treatment: a systematic review and Bayesian network meta-analysis. Front. Oncol. 10, 519388. 10.3389/fonc.2020.519388 33072564 PMC7531177

[B6] Chi K. N. Agarwal N. Bjartell A. Chung B. H. Pereira de Santana Gomes A. J. Given R. (2019). Apalutamide for metastatic, castration-sensitive prostate cancer. N. Engl. J. Med. 381, 13–24. 10.1056/NEJMoa1903307 31150574

[B7] Chi K. N. Chowdhury S. Bjartell A. Chung B. H. Pereira de Santana Gomes A. J. Given R. (2021). Apalutamide in patients with metastatic castration-sensitive prostate cancer: final survival analysis of the randomized, double-blind, Phase III TITAN study. J. Clin. Oncol. 39, 2294–2303. 10.1200/JCO.20.03488 33914595

[B8] Ciccarese C. Iacovelli R. Sternberg C. N. Gillessen S. Tortora G. Fizazi K. (2022). Triplet therapy with androgen deprivation, docetaxel, and androgen receptor signalling inhibitors in metastatic castration-sensitive prostate cancer: a meta-analysis. Eur. J. Cancer 173, 276–284. 10.1016/j.ejca.2022.07.011 35964470

[B9] Davis I. D. Martin A. J. Stockler M. R. Begbie S. Chi K. N. Chowdhury S. (2019). Enzalutamide with standard first-line therapy in metastatic prostate cancer. N. Engl. J. Med. 381, 121–131. 10.1056/NEJMoa1903835 31157964

[B10] Dou M. Liang H. Liu Y. Zhang Q. Li R. Chen S. (2023). Based on ARASENS trial: efficacy and safety of darolutamide as an emerging option of endocrinotherapy for metastatic hormone-sensitive prostate cancer-an updated systematic review and network meta-analysis. J. Cancer Res. Clin. Oncol. 149, 7017–7027. 10.1007/s00432-023-04658-6 36856851 PMC11798062

[B11] Fizazi K. Foulon S. Carles J. Roubaud G. McDermott R. Fléchon A. (2022). Abiraterone plus prednisone added to androgen deprivation therapy and docetaxel in *de novo* metastatic castration-sensitive prostate cancer (PEACE-1): a multicentre, open-label, randomised, phase 3 study with a 2 × 2 factorial design. Lancet 399, 1695–1707. 10.1016/S0140-6736(22)00367-1 35405085

[B12] Hhoyle A. P. Ali A. James N. D. Cook A. Parker C. C. de Bono J. S. (2019). Abiraterone in “high-” and “low-risk” metastatic hormone-sensitive prostate cancer. Eur. Urol. 76, 719–728. 10.1016/j.eururo.2019.08.006 31447077

[B13] Hoyle A. P. Ali A. James N. D. Cook A. Parker C. C. de Bono J. S. (2019). Abiraterone in “high-” and “low-risk” metastatic hormone-sensitive prostate cancer. Eur. Urol. 76, 719–728. 10.1016/j.eururo.2019.08.006 31447077

[B14] Hussain M. Fizazi K. Shore N. D. Heidegger I. Smith M. R. Tombal B. (2024). Metastatic hormone-sensitive prostate cancer and combination treatment outcomes: a review. JAMA Oncol. 10 (6), 807–820. 10.1001/jamaoncol.2024.0591 38722620

[B15] Hussain M. Tombal B. Saad F. Fizazi K. Sternberg C. N. Crawford E. D. (2023). Darolutamide plus androgen-deprivation therapy and docetaxel in metastatic hormone-sensitive prostate cancer by disease volume and risk subgroups in the phase III ARASENS trial. J. Clin. Oncol. 41, 3595–3607. 10.1200/JCO.23.00041 36795843

[B16] Hutton B. Salanti G. Caldwell D. M. Chaimani A. Schmid C. H. Cameron C. (2015). The PRISMA extension statement for reporting of systematic reviews incorporating network meta-analyses of health care interventions: checklist and explanations. Ann. Intern Med. 162, 777–784. 10.7326/M14-2385 26030634

[B17] James N. D. de Bono J. S. Spears M. R. Clarke N. W. Mason M. D. Dearnaley D. P. (2017). Abiraterone for prostate cancer not previously treated with hormone therapy. N. Engl. J. Med. 377, 338–351. 10.1056/NEJMoa1702900 28578639 PMC5533216

[B18] James N. D. Sydes M. R. Clarke N. W. Mason M. D. Dearnaley D. P. Spears M. R. (2016). Addition of docetaxel, zoledronic acid, or both to first-line long-term hormone therapy in prostate cancer (STAMPEDE): survival results from an adaptive, multiarm, multistage, platform randomised controlled trial. Lancet 387, 1163–1177. 10.1016/s0140-6736(15)01037-5 26719232 PMC4800035

[B19] Jian T. Zhan Y. Hu K. He L. Chen S. Hu R. (2022). Systemic triplet therapy for metastatic hormone-sensitive prostate cancer: a systematic review and network meta-analysis. Front. Pharmacol. 13, 955925. 10.3389/fphar.2022.955925 36278154 PMC9582339

[B20] Kinsey E. N. Zhang T. Armstrong A. J. (2020). Metastatic hormone-sensitive prostate cancer: a review of the current treatment landscape. Cancer J. 26, 64–75. 10.1097/PPO.0000000000000418 31977388

[B21] Maiorano B. A. De Giorgi U. Roviello G. Messina C. Altavilla A. Cattrini C. (2022). Addition of androgen receptor-targeted agents to androgen-deprivation therapy and docetaxel in metastatic hormone-sensitive prostate cancer: a systematic review and meta-analysis. ESMO Open 7, 100575. 10.1016/j.esmoop.2022.100575 36152486 PMC9588886

[B22] Mandel P. Hoeh B. Wenzel M. Preisser F. Tian Z. Tilki D. (2023). Triplet or doublet therapy in metastatic hormone-sensitive prostate cancer patients: a systematic review and network meta-analysis. Eur. Urol. Focus 9, 96–105. 10.1016/j.euf.2022.08.007 36058809

[B23] Menges D. Yebyo H. G. Sivec-Muniz S. Haile S. R. Barbier M. C. Tomonaga Y. (2022). Treatments for metastatic hormone-sensitive prostate cancer: systematic review, network meta-analysis, and benefit-harm assessment. Eur. Urol. Oncol. 5, 605–616. 10.1016/j.euo.2022.04.007 35599144

[B24] Mohler J. L. Antonarakis E. S. Armstrong A. J. D'Amico A. V. Davis B. J. Dorff T. (2019). Prostate cancer, version 2.2019, NCCN clinical practice guidelines in oncology. J. Netal Comper Canc Netw. 17, 479–505. 10.6004/jnccn.2019.0023 31085757

[B25] Mori K. Mostafaei H. Sari Motlagh R. Pradere B. Quhal F. Laukhtina E. (2022). Systemic therapies for metastatic hormone-sensitive prostate cancer: network metaanalysis. BJU Int. 129, 423–433. 10.1111/bju.15507 34171173 PMC9291853

[B27] Nader R. El Amm J. Aragon-Ching J. B. (2018). Role of chemotherapy in prostate cancer. Asian J. Androl. 20, 221–229. 10.4103/aja.aja_40_17 29063869 PMC5952475

[B28] Page M. J. McKenzie J. E. Bossuyt P. M. Boutron I. Hoffmann T. C. Mulrow C. D. (2021). The PRISMA 2020 statement: an updated guideline for reporting systematic reviews. BMJ 372, n71. 10.1136/bmj.n71 33782057 PMC8005924

[B29] Rice M. A. Malhotra S. V. Stoyanova T. (2019). Second-generation antiandrogens: from discovery to standard of care in castration resistant prostate cancer. Front. Oncol. 9, 801. 10.3389/fonc.2019.00801 31555580 PMC6723105

[B30] Rizzo M. (2021). Mechanisms of docetaxel resistance in prostate cancer: the key role played by miRNAs. Biochim. Biophys. Acta Rev. Cancer 1875, 188481. 10.1016/j.bbcan.2020.188481 33217485

[B31] Rücker G. Schwarzer G. (2016). Automated drawing of network plots in network meta-analysis. Res. Synth. Methods 7, 94–107. 10.1002/jrsm.1143 26060934

[B32] Sartor O. de Bono J. S. (2018). Metastatic prostate cancer. N. Engl. J. Med. 378, 645–657. 10.1056/NEJMra1701695 29412780

[B33] Shim S. R. Kim S. J. Rucker G. (2019). Network meta-analysis: application and practice using R software. Epidemiol. Health 41, e2019013. 10.4178/epih.e2019013 30999733 PMC6635665

[B34] Siegel R. L. Miller K. D. Fuchs H. E. Jemal A. (2022). Cancer statistics, 2022. CA Cancer J. Clin. 72, 7–33. 10.3322/caac.21708 35020204

[B35] Smith M. R. Hussain M. Saad F. Fizazi K. Sternberg C. N. Crawford E. D. (2022). Darolutamide and survival in metastatic hormone-sensitive prostate cancer. N. Engl. J. Med. 386, 1132–1142. 10.1056/nejmoa2119115 35179323 PMC9844551

[B36] Sterne J. A. C. Savović J. Page M. J. Elbers R. G. Blencowe N. S. Boutron I. (2019). RoB 2: a revised tool for assessing risk of bias in randomised trials. BMJ 366, l4898. 10.1136/bmj.l4898 31462531

[B37] Sung H. Ferlay J. Siegel R. L. Laversanne M. Soerjomataram I. Jemal A. (2021). Global cancer statistics 2020: GLOBOCAN estimates of incidence and mortality worldwide for 36 cancers in 185 countries. CA Cancer J. Clin. 71, 209–249. 10.3322/caac.21660 33538338

[B38] Sweeney C. J. Chen Y. H. Carducci M. Liu G. Jarrard D. F. Eisenberger M. (2015). Chemohormonal therapy in metastatic hormone-sensitive prostate cancer. N. Engl. J. Med. 373, 737–746. 10.1056/NEJMoa1503747 26244877 PMC4562797

[B39] Sydes M. R. Spears M. R. Mason M. D. Clarke N. W. Dearnaley D. P. de Bono J. S. (2018). Adding abiraterone or docetaxel to long-term hormone therapy for prostate cancer: directly randomised data from the STAMPEDE multi-arm, multi-stage platform protocol. Ann. Oncol. 29, 1235–1248. 10.1093/annonc/mdy072 29529169 PMC5961425

[B40] Tannock I. F. Wit R. D. Berry W. R. Horti J. Pluzanska A. Chi K. N. (2004). Docetaxel plus prednisone or mitoxantrone plus predisone for advanced prostate cancer. N. Engl. J. Med. 351, 1502–1512. 10.1056/NEJMoa040720 15470213

[B41] Thadani-Mulero M. Nanus D. M. Giannakakou P. (2012). Androgen receptor on the move: boarding the microtubule expressway to the nucleus. Cancer Res. 72, 4611–4615. 10.1158/0008-5472.CAN-12-0783 22987486 PMC3448065

[B42] Tilki D. van den Bergh R. C. N. Briers E. Van den Broeck T. Brunckhorst O. Darraugh J. (2024). EAU-EANM-ESTRO-ESUR-ISUP-SIOG guidelines on prostate cancer. Part II-2024 update: treatment of relapsing and metastatic prostate cancer. Eur. Urol. 86 (2), 164–182. 10.1016/j.eururo.2024.04.010 38688773

[B43] Uhlmann L. Jensen K. Kieser M. (2018). Hypothesis testing in Bayesian network meta-analysis. BMC Med. Res. Methodol. 18 (1), 128. 10.1186/s12874-018-0574-y 30419827 PMC6233362

[B44] Wang L. Li C. Zhao Z. Li X. Tang C. Guan Z. (2023). Comparison of doublet and triplet therapies for metastatic hormone-sensitive prostate cancer: a systematic review and network meta-analysis. Front. Oncol. 13, 1104242. 10.3389/fonc.2023.1104242 36959793 PMC10028133

[B45] Wang L. Paller C. J. Hong H. De Felice A. Alexander G. C. Brawley O. (2021). Comparison of systemic treatments for metastatic castration-sensitive prostate cancer: a systematic review and network meta-analysis. JAMA Oncol. 7, 412–420. 10.1001/jamaoncol.2020.6973 33443584 PMC7809610

[B46] Woods B. S. Hawkins N. Scott D. A. (2010). Network meta-analysis on the log-hazard scale, combining count and hazard ratio statistics accounting for multi-arm trials: a tutorial. BMC Med. Res. Methodol. 10, 54. 10.1186/1471-2288-10-54 20537177 PMC2906500

[B47] Yanagisawa T. Rajwa P. Thibault C. Gandaglia G. Mori K. Kawada T. (2022). Androgen receptor signaling inhibitors in addition to docetaxel with androgen deprivation therapy for metastatic hormone-sensitive prostate cancer: a systematic review and meta-analysis. Eur. Urol. 82, 584–598. 10.1016/j.eururo.2022.08.002 35995644

