# Supplementary material for: Sequential versus concomitant treatment of androgen receptor signaling inhibitors and docetaxel for metastatic hormone-sensitive prostate cancer: an network meta-analysis
Source: Front Pharmacol. 2024 Oct 28;15:1462360. doi: 10.3389/fphar.2024.1462360 (PMC11551020; doi:10.3389/fphar.2024.1462360)
Supplement: Supplementary file 1 [file DataSheet1.docx]

Supplementary Material for

**Docetaxel with Androgen Deprivation Therapy Sequential and Combined with Novel Endocrine Therapy for Metastatic Hormone-sensitive Prostate Cancer： A Systematic Review and Network Meta-analysis**

Supplementary Material 1：Search strategy

Supplementary Material 2：Risk of study bias for OS (A) /generalized PFS (B) /PSA (C)

Supplementary Material 3：Patients’ supplementary information of included trials.

Supplementary Material 4：Hazard Ratio for OS and PFS in included trials

Supplementary Material 5：Network plots

Supplementary Material 6：Supplementary forest plots

Supplementary Material 7：Heterogeneity test

Supplementary Material 8：Relative effect estimates for all possible treatment comparisons

Supplementary Material 9：OS/PFS ranking and ranking probability

Supplementary Material 10：Trace and density plots

Supplementary Material 11：SUCRA plots

Supplementary Material 12：AE data

**Supplementary Material 1： Search strategy**

MEDLINE (PubMed interface)

Related to mHSPC

#1"metastatic"[Title/Abstract]

#2 "metastasis"[Title/Abstract]

#3"advanced"[Title/Abstract]

#4 #1 OR #2 OR #3

#5 "Hormones"[MeSH Terms]

#6 "castration"[MeSH Terms]

#7 "Hormone"[Title/Abstract]

#8 "castration"[Title/Abstract]

#9 #5 OR #6 OR #7 OR #8

#10 "sensitive"[Title/Abstract]

#11 #9 AND #10

#12 " Prostatic Neoplasms "[MeSH Terms]

#13 " Prostate Neoplasms "[Title/Abstract]

#14 " Prostate Neoplasm"[Title/Abstract]

#15 " Prostatic Neoplasm "[Title/Abstract]

#16 "Prostate Cancer" [Title/Abstract]

#17 "Prostate Cancers" [Title/Abstract]

#18 "Cancer of the Prostate " [Title/Abstract]

#19 "Prostatic Cancer " [Title/Abstract]

#20 " Prostatic Cancers "[Title/Abstract]

#21 " Cancer of Prostate "[Title/Abstract]

#22 " prostatic carcinoma "[Title/Abstract]

#23 #12 OR #13 OR #14 OR #15 OR #16 OR #17 OR #18 OR #19 OR #20 OR #21 OR #22

#24 #4 AND #11 AND #23

Related to RCT

#25 "Randomized Controlled Trials as Topic "[MeSH Terms]

#26 "randomized control trial "[Title/Abstract]

#27 "Randomized Controlled Trials "[Title/Abstract]

#28 "RCT"[Title/Abstract]

#29 "RCTs"[Title/Abstract]

#30 "randomized "[Title/Abstract]

#31 "randomly "[Title/Abstract]

#32 "placebo"[Title/Abstract]

#33 " trial "[Title/Abstract]

#34 #25 OR #26 OR#27 OR #28 OR #29 OR #30 OR #31 OR #32 OR #33

Combined items

#35 #24 AND #34

EMBASE

Related to mHSPC

#1 metastatic：ti,ab,kw

#2 metastasis：ti,ab,kw

#3 advanced：ti,ab,kw

#4 #1 OR #2 OR #3

#5 'hormones'/de

#6 'castration'/de

#7 hormone：ti,ab,kw

#8 castrations：ti,ab,kw

#9 #5 OR #6 OR #7 OR #8

#10 sensitive：ti,ab,kw

#11 #9 AND #10

#12 'prostatic neoplasms'/de

#13'prostate neoplasms'：ti,ab,kw

#14 'prostate neoplasm'：ti,ab,kw

#15 ' prostatic neoplasm'：ti,ab,kw

#16 ' prostate cancer '：ti,ab,kw

#17 ' prostate cancers '：ti,ab,kw

#18 'cancer of the prostate '：ti,ab,kw

#19 'prostatic cancer '：ti,ab,kw

#20 'prostatic cancers '：ti,ab,kw

#21' cancer of prostate '：ti,ab,kw

#22 ' prostatic carcinoma '：ti,ab,kw

#22 'prostate neoplasm'：ti,ab,kw

#23 #12 OR #13 OR #14 OR #15 OR #16 OR #17 OR #18 OR #19 OR #20 OR #21 OR #22

#24 #4 AND #11 AND #23

Related to RCT

#25'randomized controlled trials as topic'/de

#26 'randomized control trial'：ti,ab,kw

#27'randomized controlled trials'：ti,ab,kw

#28 rct：ti,ab,kw

#29 rcts：ti,ab,kw

#30 randomized：ti,ab,kw

#31 randomly：ti,ab,kw

#32 placebo：ti,ab,kw

#33 trial：ti,ab,kw

#34 #25 OR #26 OR#27 OR #28 OR #29 OR #30 OR #31 OR #32 OR #33

Combined items

#35 #24 AND #34

Cochrane CENTRAL

Related to mHSPC

#1 (metastatic)：ti,ab,kw

#2 (metastasis)：ti,ab,kw

#3 (advanced)：ti,ab,kw

#4 Hormones

#5 castration

#6 (Hormone)：ti,ab,kw

#7 (castrations)：ti,ab,kw

#8 (sensitive)：ti,ab,kw

#9 "Prostatic Neoplasms"

#10 ("Prostate Neoplasms")：ti,ab,kw

#11 (“Prostate Neoplasm”)：ti,ab,kw

#12 (“Prostatic Neoplasm”)：ti,ab,kw

#13 (“Prostate Cancer”)：ti,ab,kw

#14 (“Prostate Cancers”)：ti,ab,kw

#15 (“Cancer of the Prostate”)：ti,ab,kw

#16 (“Prostatic Cancer”)：ti,ab,kw

#17 (“Prostatic Cancers”)：ti,ab,kw

#18 (“Cancer of Prostate”)：ti,ab,kw

#19 (“prostatic carcinoma”)：ti,ab,kw

#20 #1 or #2 or #3

#21 #4 or #5 or #6 or #7

#22 #9 or #10 or #11 or #12 or #13 or #14 or #15 or #16 or #17 or #18 or #19

#23 #20 and #21 and #8 and #22

Related to RCT

#24 “Randomized Controlled Trials as Topic”

#25 (“randomized control trial”)：ti,ab,kw

#26 (“Randomized Controlled Trials”)：ti,ab,kw

#27 (RCT)：ti,ab,kw

#28 (RCTs)：ti,ab,kw

#29 (randomized)：ti,ab,kw

#30 (randomly)：ti,ab,kw

#31 (placebo)：ti,ab,kw

#32 (trial)：ti,ab,kw

#33 #24 or #25 or #26 or #27 or #28 or #29 or #30 or #31 or #32

Combined items

#34 #23 and #33

***ClinicalTrials.gov***

#1 Condition or disease： Metastatic Hormone-Sensitive Prostate Cancer

#2 Condition or disease： Prostatic Neoplasm

#3 Condition or disease： Prostatic

#4Condition or disease： Neoplasm

#5 Study Results： Studies With Results

**Supplementary Material 2： Risk of study bias for OS (A) / generalized PFS (B) /PSA (C)**

OS：Overall Survival；generalized PFS：generalized Progression-free Survival；PSA：Prostate-Specific Antigen


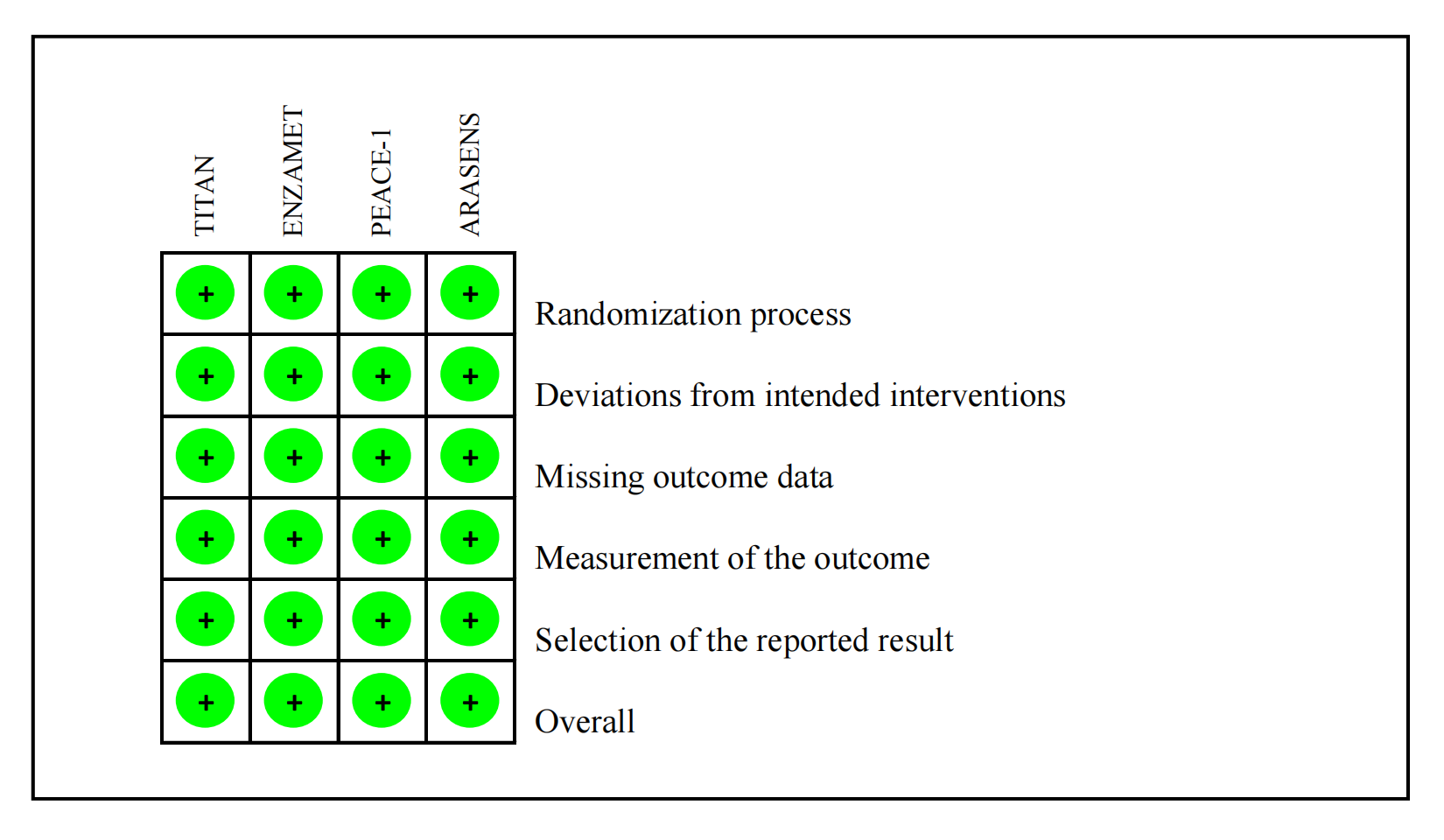

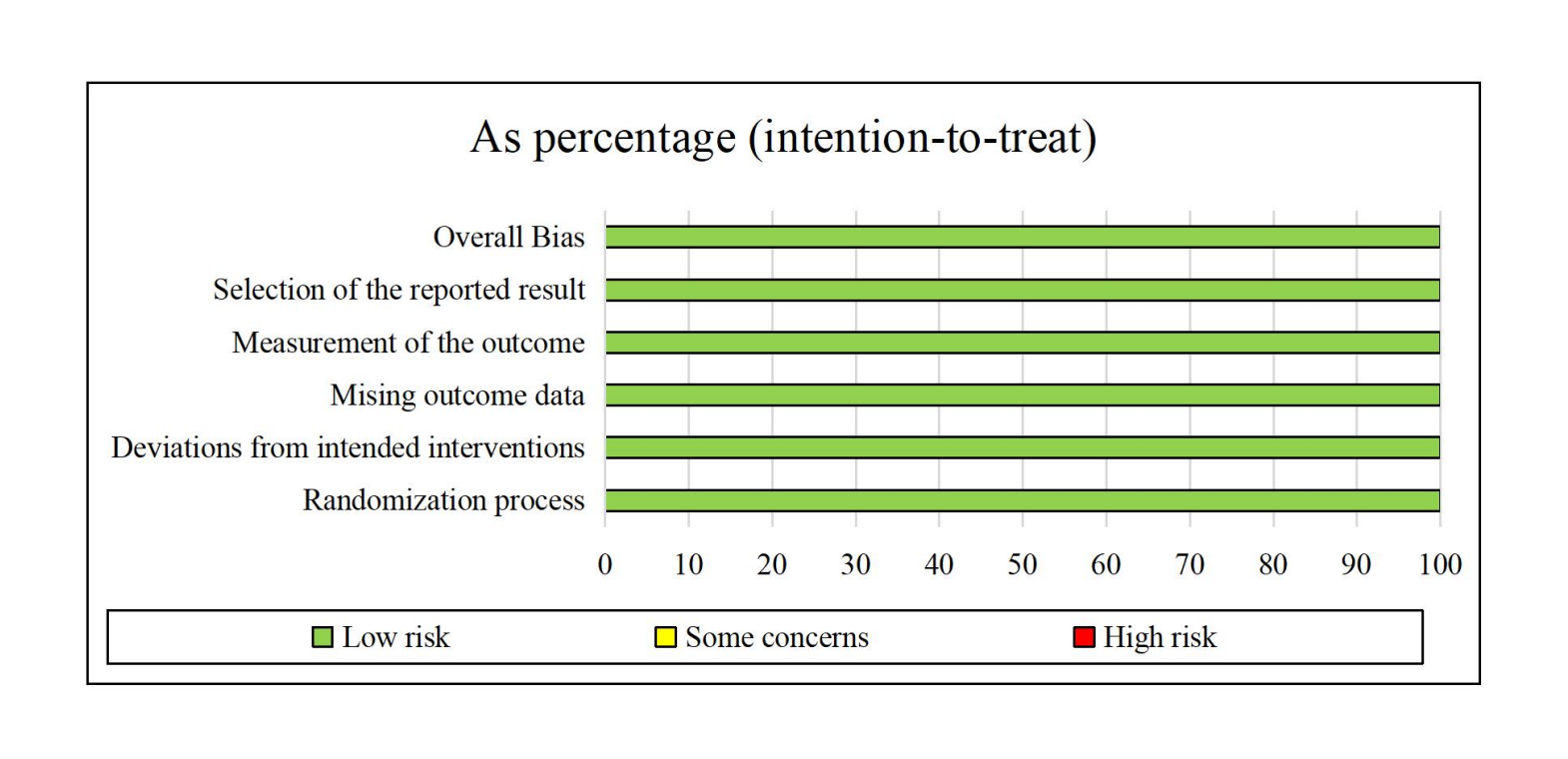


**A (OS)**


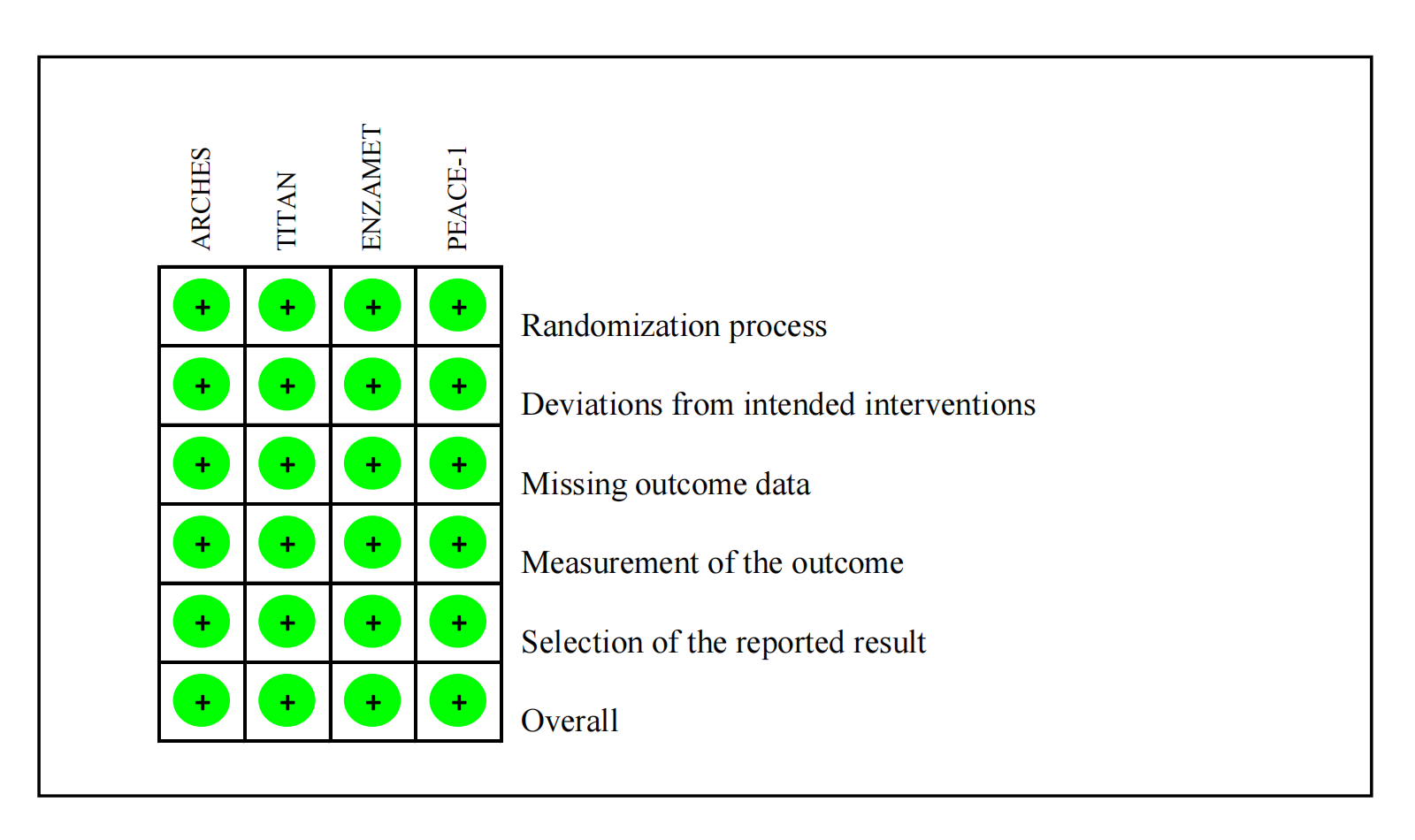

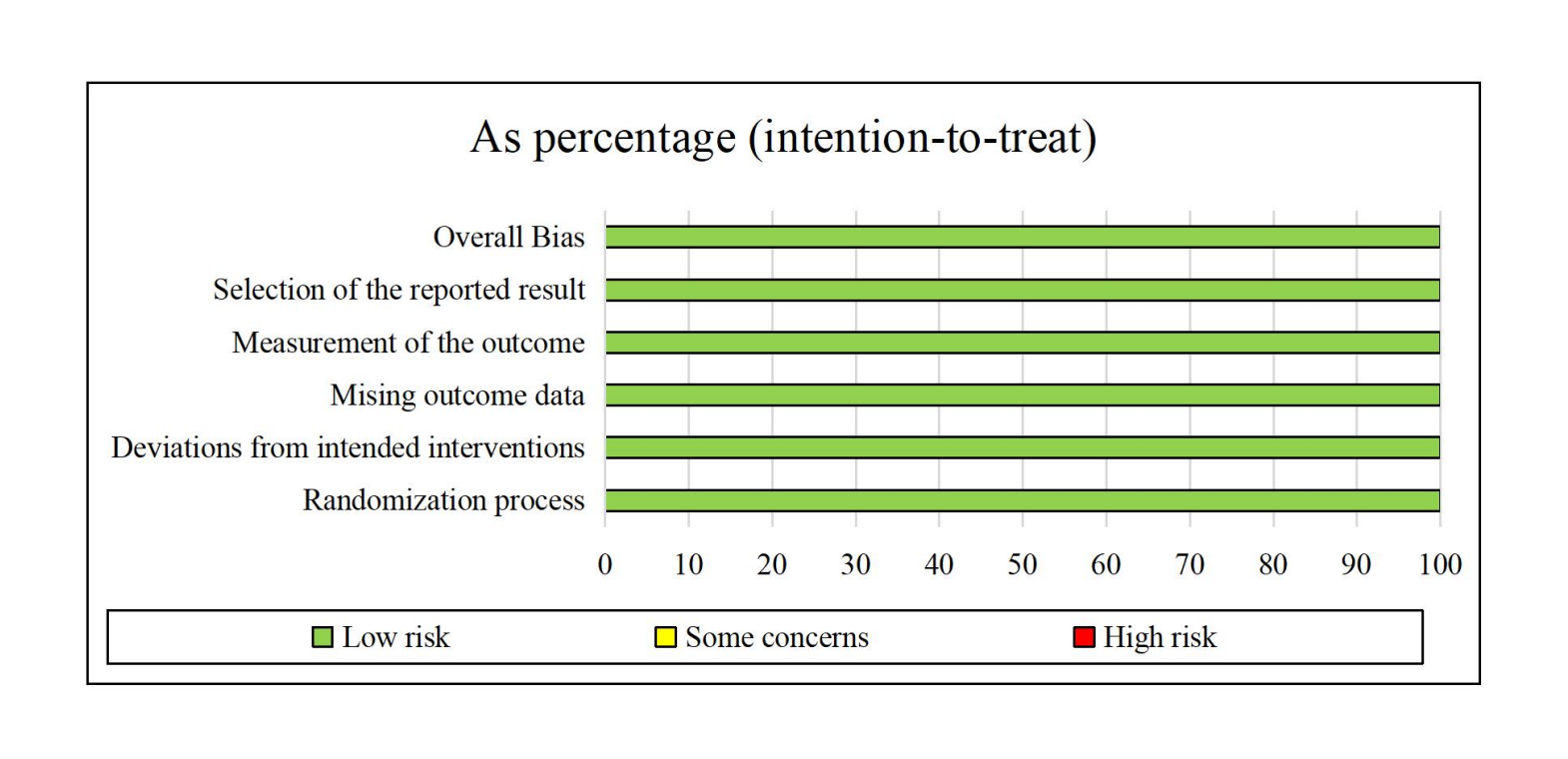


**B (generalized PFS)**


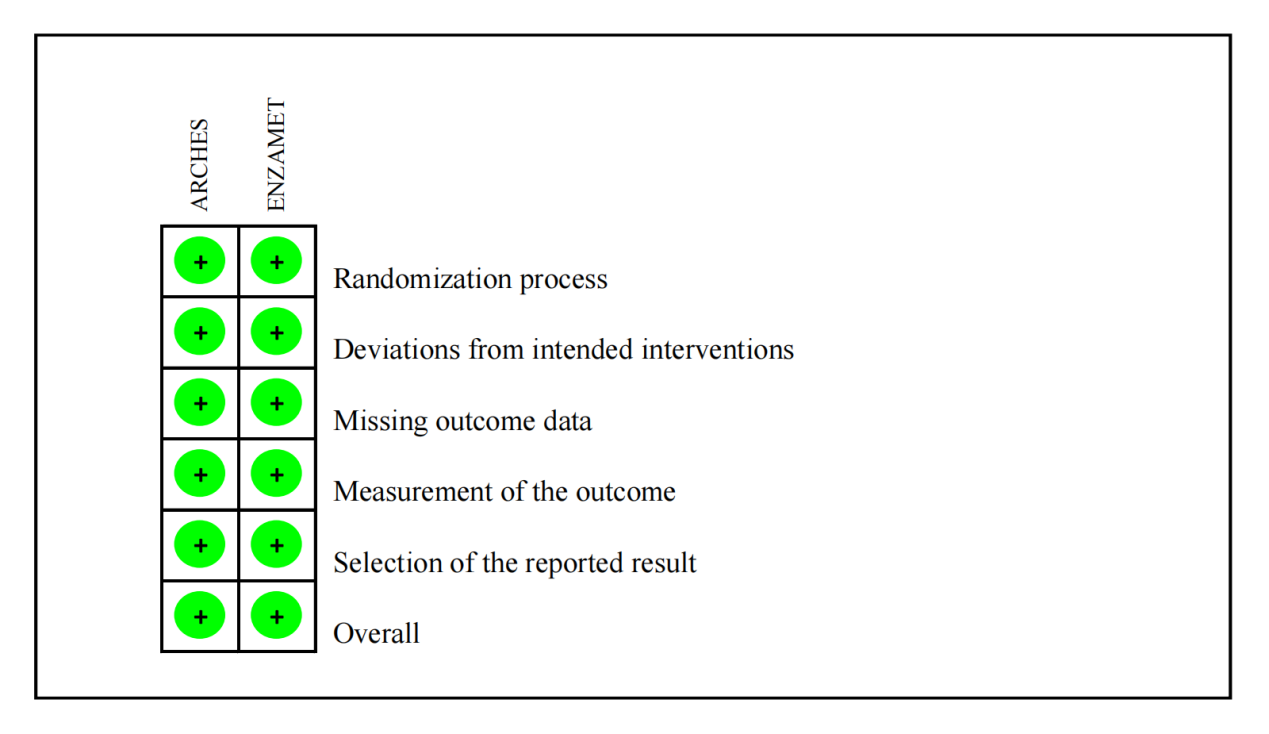

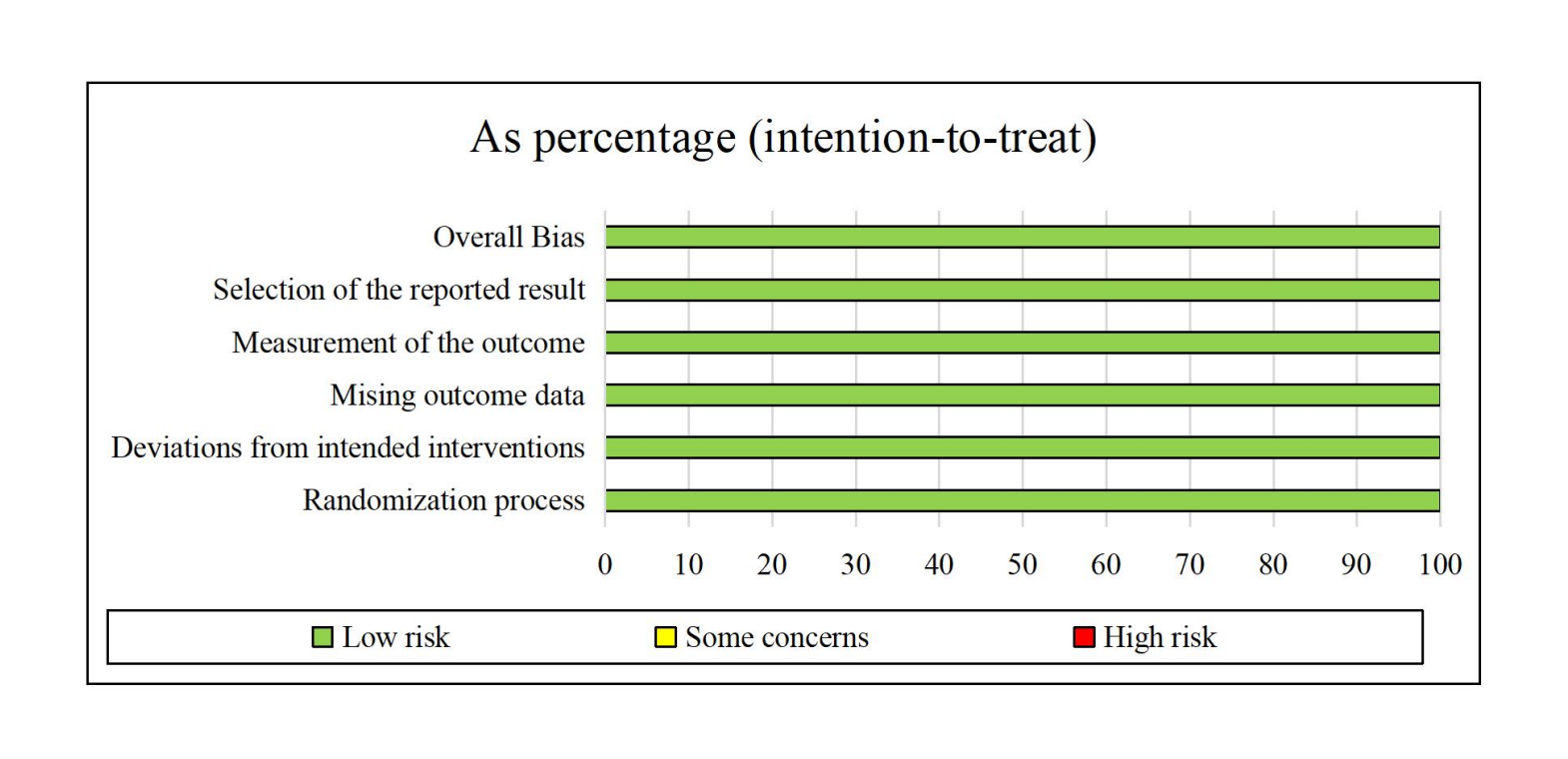


**C (PSA)**

**Supplementary Material 3： Patients’ supplementary information of included trials.**

| Trial | Number of patients((Exp. vs. Ctrl, no. %)) | Performance status  (ECOG/WHO grade, %) | Age (Exp. vs. Ctrl, yrs) | PSA (Exp. vs. Ctrl, ng/ml) | Gleason score (Exp. vs. Ctrl, no. %) | Visceral metastases(Exp. vs. Ctrl, no. %). | Metastatic burden  (CHAARTED definition, Exp. vs. Ctrl, no. %) | Metastases at initial diagnosis (Exp. vs. Ctrl, no. %) |
| --- | --- | --- | --- | --- | --- | --- | --- | --- |
| ARCHES | 103(50) vs102(50) | NR | NR | NR | NR | NR | NR | NR |
| TITAN | 58(51) vs 55(49) | NR | NR | NR | NR | NR | NR | NR |
| ENZAMET | 254(45)vs. 249(44) | NR | NR | NR | NR | NR | LVD：77(30) vs. 70(28)  HVD：177(70) vs. 179(72) | NR |
| PEACE-1 | 355(50) vs 355(50) | Grade 0, 69.9%  Grade 1-2, 30.1% | Median(IQR)：  66(60-70)vs. 66(59-70) | Median(IQR)：14(2-59) vs. 12(3-60)  Unknown：0 vs 2 | ≤7：79(23) vs.71(20)  8-10：270(77) vs.276(80)  Unknown： 6(2) vs 8(2) | 41(12)vs.47(13) | LVD：131(37) vs. 123(35)  HVD： 224(63) vs. 232(65) | Total：710(100) |
| ARASENS | 651(50) vs 654(50) | Grade 0, 71.1%  Grade 1, 28.7% | Median(Range)：  67(41-89)vs. 67(42-86) | Median(Range)：  30.3(0-9219) vs.24.2 (0-11947) | ＜8： 122(18.7) vs.118(18)  ≥8： 505(77.6)vs.516(78.9)  Unknown：24(3.7) vs.20(3.1) | 111(17)vs.118(18) | NR | 558(85.7)vs. 566(86.5)  unknown： 6 vs 7 |

Ctrl.：control group；ECOG：Eastern Cooperative Oncology Group；Exp.：experimental group；HVD：high volume disease；IQR：interquartile range；LVD：low volume disease；NR：not reported；PSA：Prostate-Specific Antigen.

**Supplementary Material 4： Hazard Ratio for OS and PFS in included trials**

| STUDY No.[Ref.] | Trial | Comparison | HR (OS  -total) | HVD-pts  number | HR (OS-HVD) | LVD-pts  number | HR (OS-LVD) | PFS type | HR(rPFS  -total) | HR(rPFS  -HVD) | HR(rPFS  -LVD) |
| --- | --- | --- | --- | --- | --- | --- | --- | --- | --- | --- | --- |
| 1 | ARCHES | ENZA + DOC + ADT vs DOC + ADT | 0.74, 0.46-1.20 | NR | NR | NR | NR | rPFS | 0.52, 0.30-0.89 | NR | NR |
| 2 | TITAN | APA + DOC + ADT vs DOC + ADT | 1.12 0.59-2.12 | NR | NR | NR | NR | rPFS | 0.47  0.22-1.01 | NR | NR |
| 3 | ENZAMET | ENZA + DOC + ADT vs DOC + ADT | 0.90 ,  0.62-1.31 | 356 | 0.97,  0.64- 1.46 | 147 | 0.65,  0.25- 1.71 | cPFS | 0.48, 0.37-0.62 | 0.51  0.38-0.69 | 0.37  0.20-0.67 |
| 4 | PEACE-1 | AAP + DOC + ADT vs DOC + ADT | 0.75, 0.59-0.95 | 456 | 0.72,  0.55-0.95 | 254 | 0.83, 0.50-1.39 | rPFS | 0.50,  0.34-0.71 | 0.47  0.30-0.72 | 0.58  0.29-1.15 |
| 5 | ARASENS | DAR + DOC + ADT vs DOC + ADT | 0.68, 0.57-0.80 | 1005 | 0.69, 0.57-0.82 | 300 | 0.68, 0.41-1.13 | NR | NR | NR | NR |

ENZA：enzalutamide；DOC：docetaxel；ADT：androgen deprivation therapy；APA：apalutamide；AAP：abiraterone；DAR：darolutamide；OS：Overall Survival；PFS：Progression Free Survival；rPFS：Radiographic Progression-free Survival；cPFS：Clinical Progression-free Survival；HR：Hazard Ratio；HVD：high volume disease；IQR：interquartile range；LVD：low volume disease；NR：not reported；、

**Supplementary Material 5： Network plots**

OS：overall survival；generalized PFS：generalized progression free survival； rPFS：radiographic progression free survival；PSA：Prostate-Specific Antigen；ADT：androgen deprivation treatment；Doc：docetaxel；Abi：abiraterone；Enz：enzalutamide；Apa：apalutamide；Dar：darolutamide；HV：high volume；LV：low volume；SAE：Serious adverse event.

A：Network plot for OS (patients with ADT + Doc + ARSi concomitant)


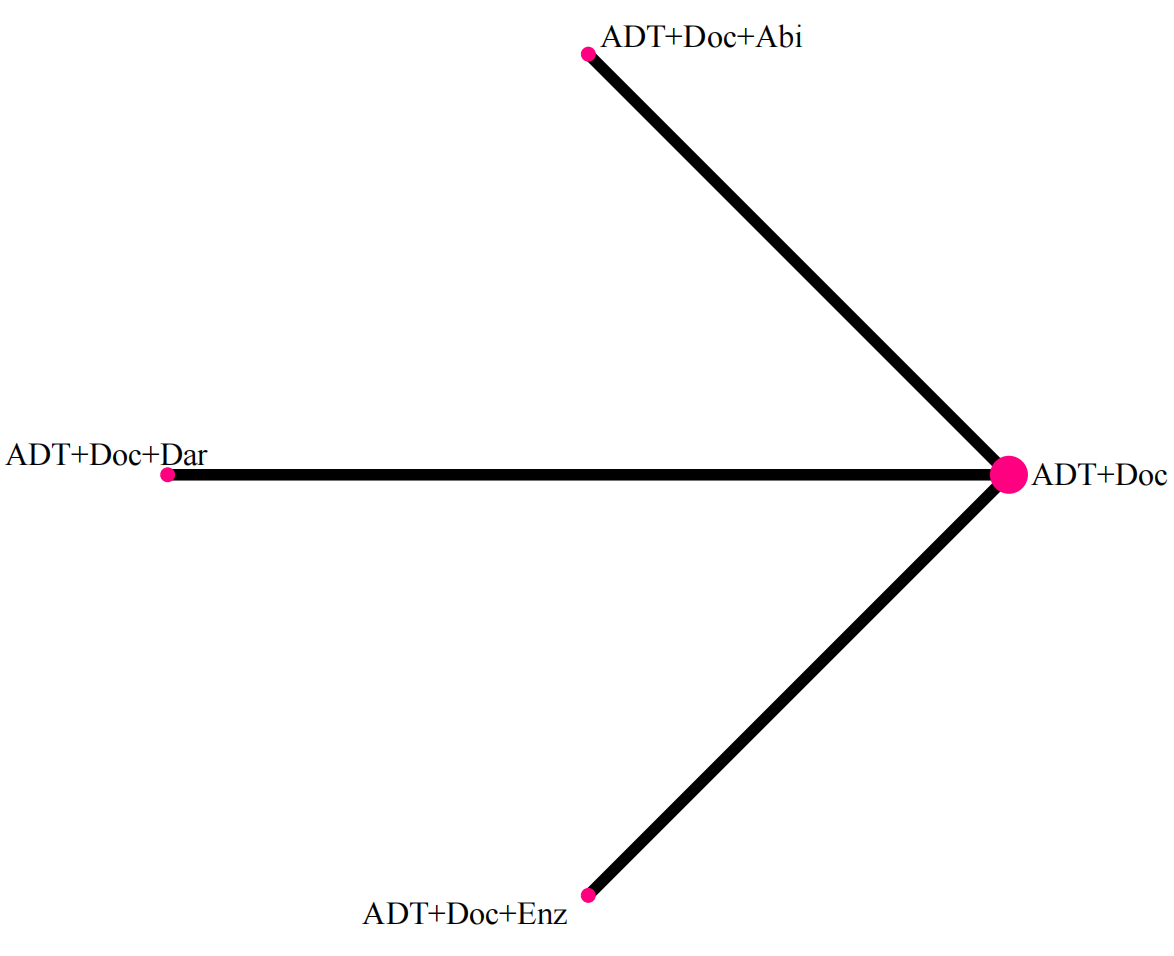


B：Network plot for OS (patients with ADT + Doc + ARSi sequential)


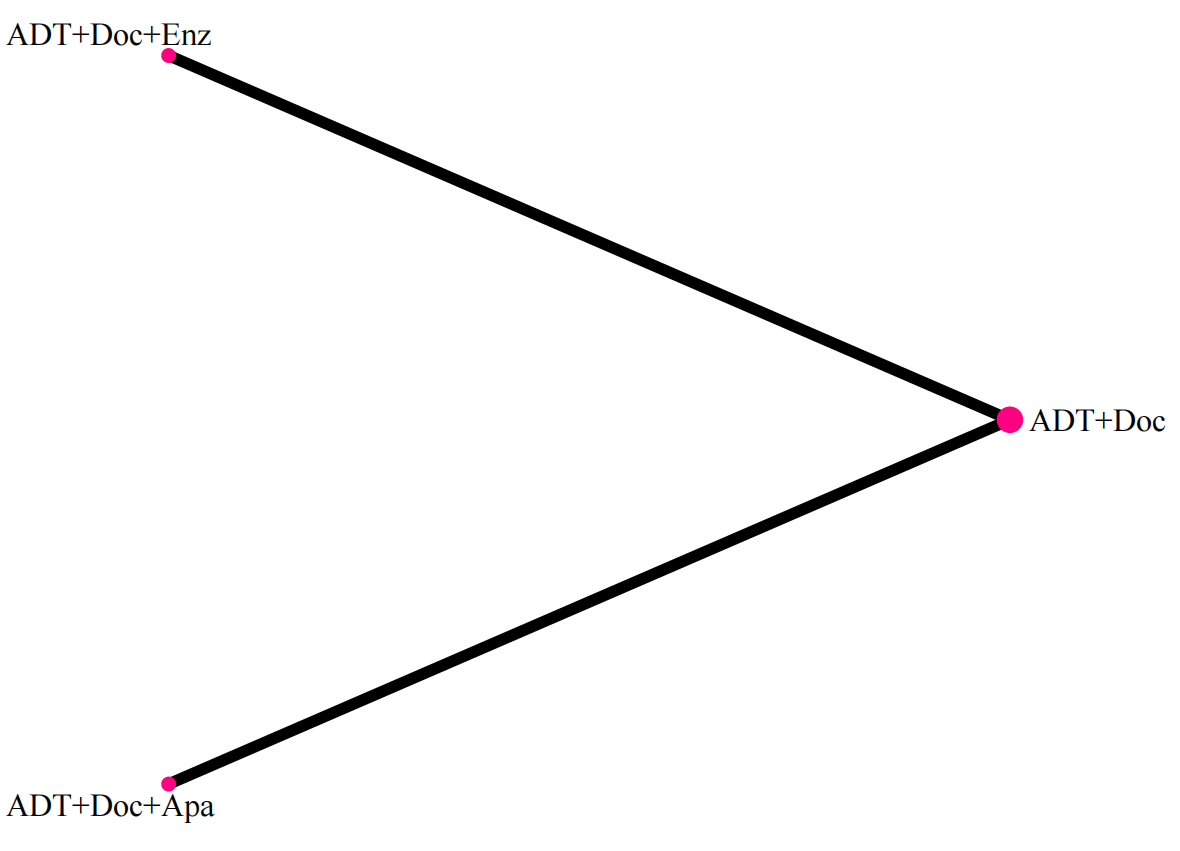


C：Network plot for OS (patients with Low volume)


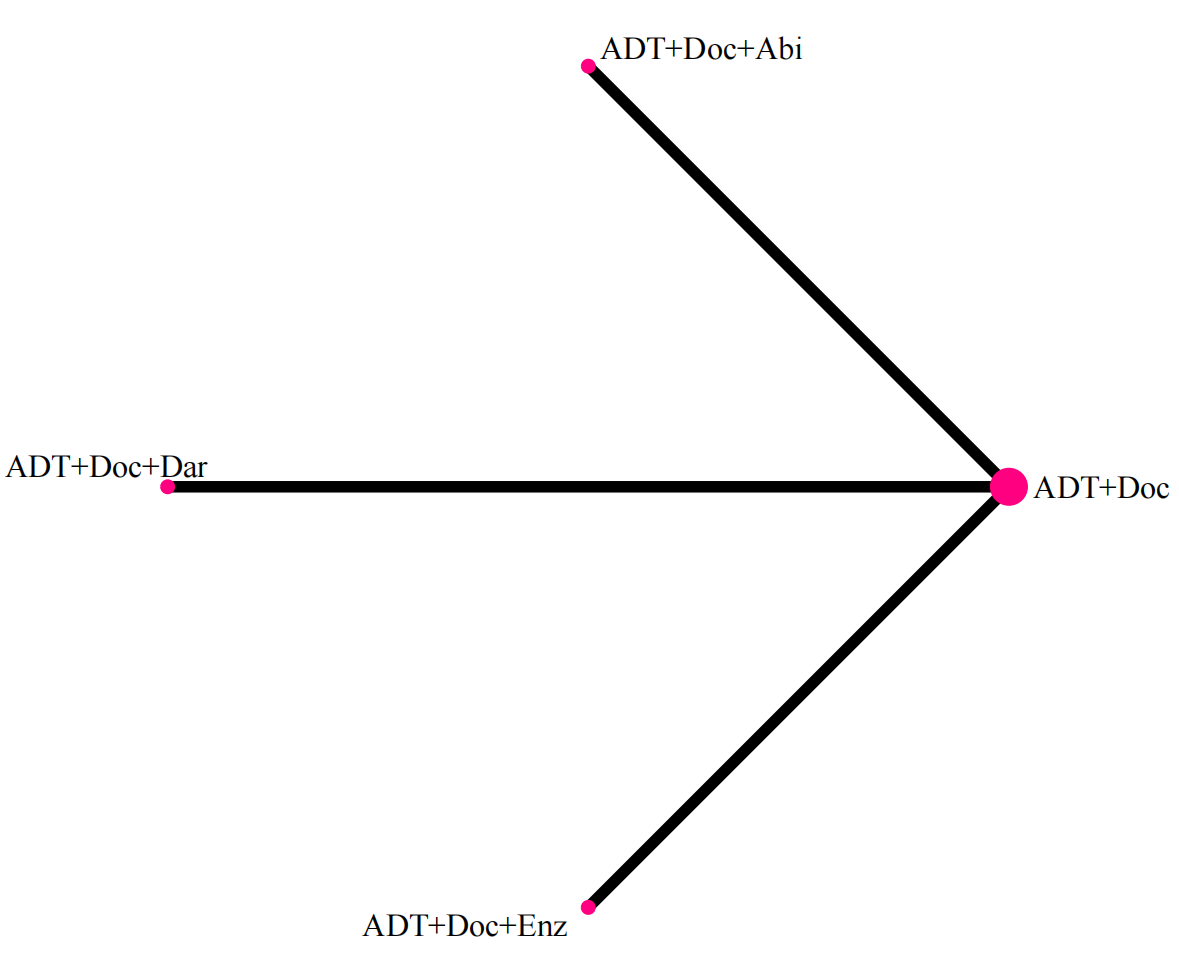


D：Network plot for OS (patients with High volume)


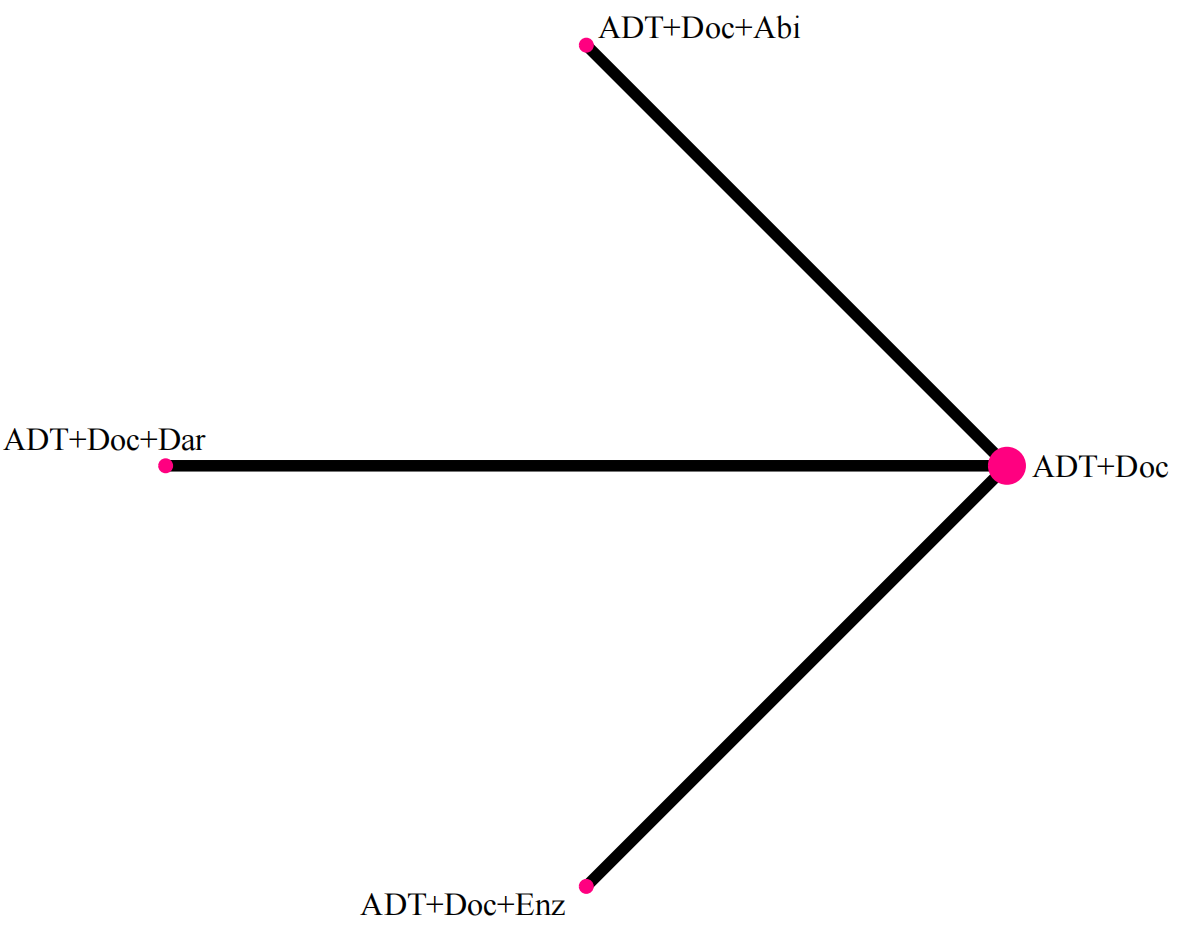


E：Network plot for generalized PFS for patients with ADT + Doc + ARSi concomitant


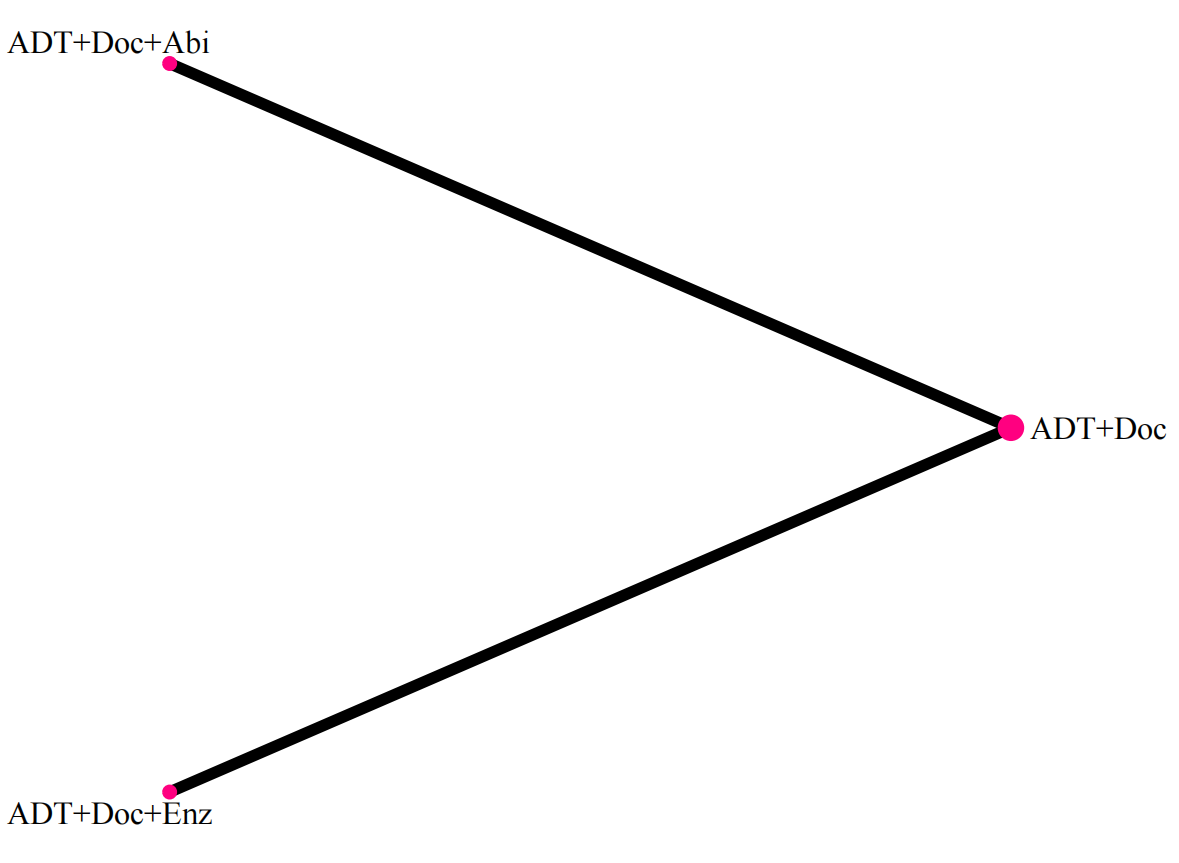


F：Network plot for rPFS for patients with ADT + Doc + ARSi sequential


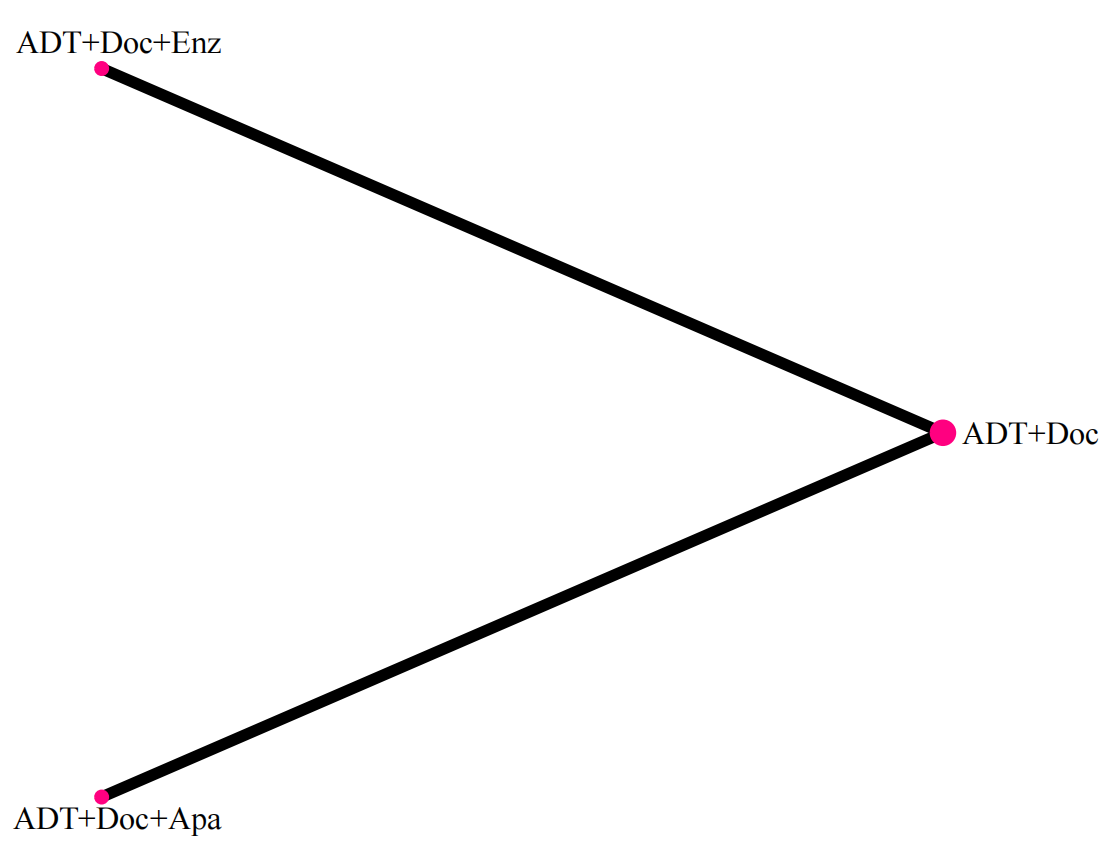


G：Network plot for generalized PFS for patients with Low volume


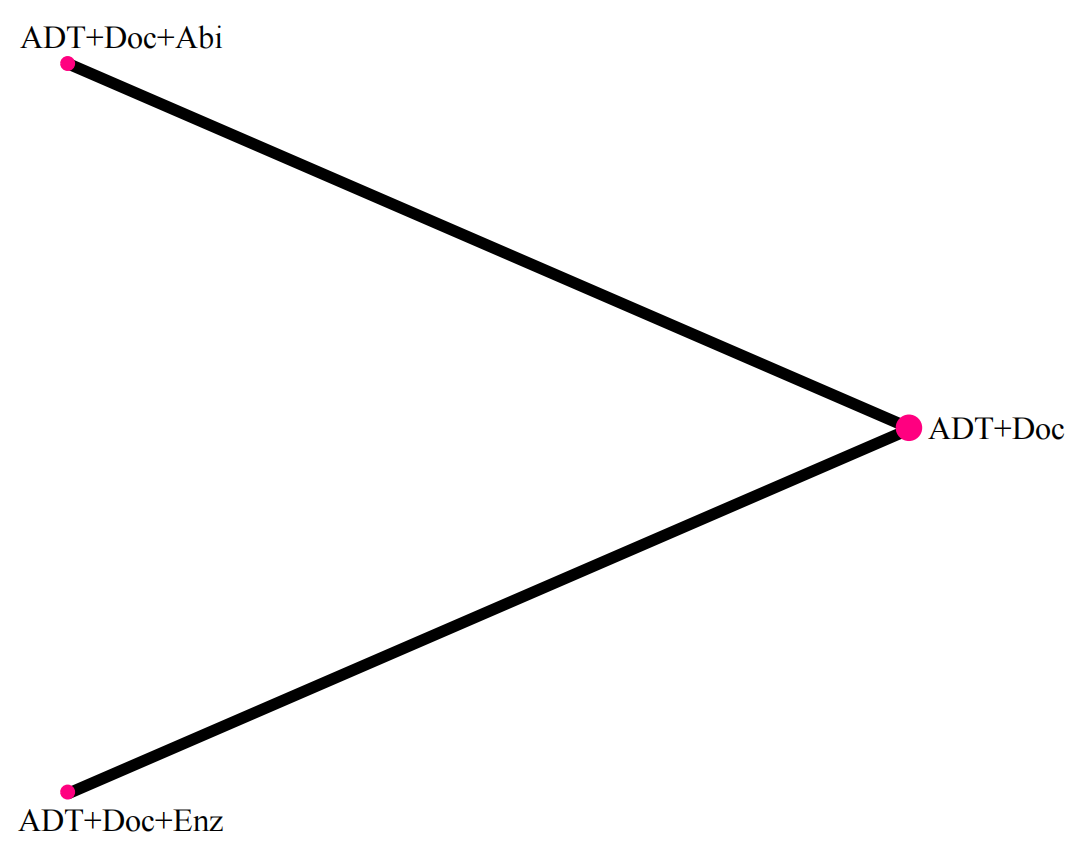


H：Network plot for generalized PFS for patients with High volume


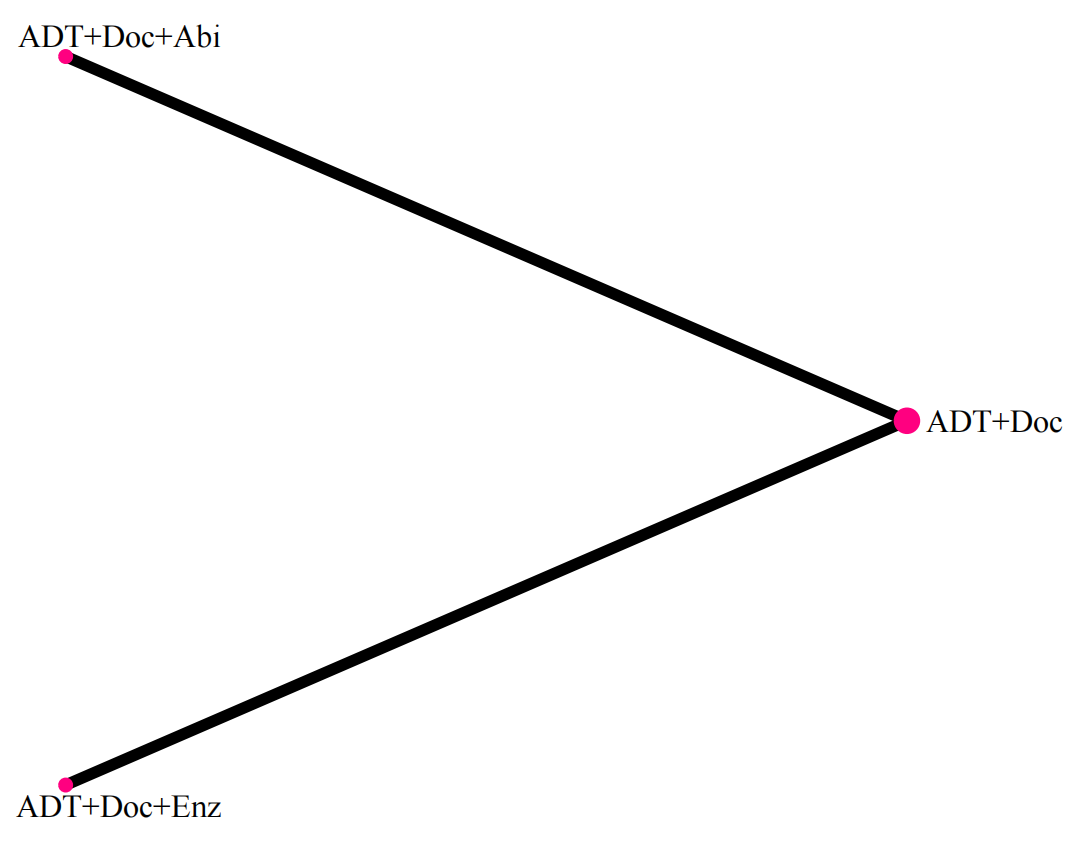


I：Network plot for PSA


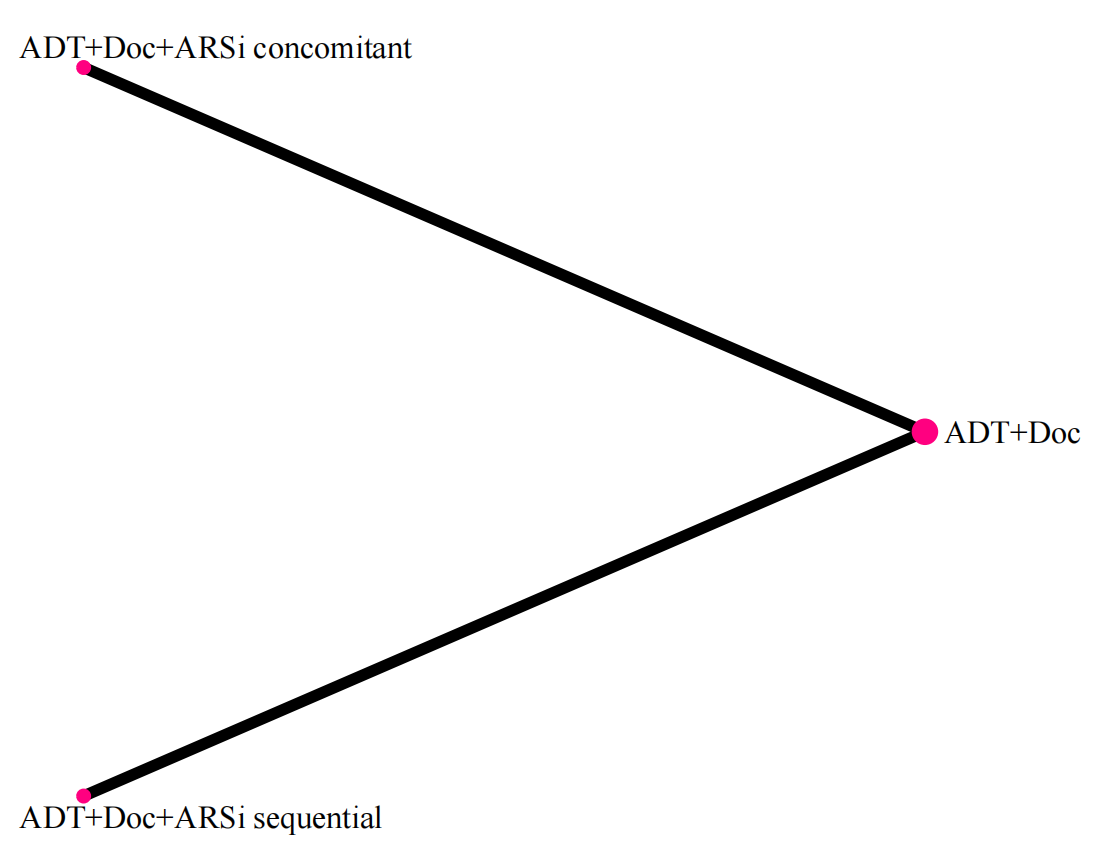


J： Network plot for SAE


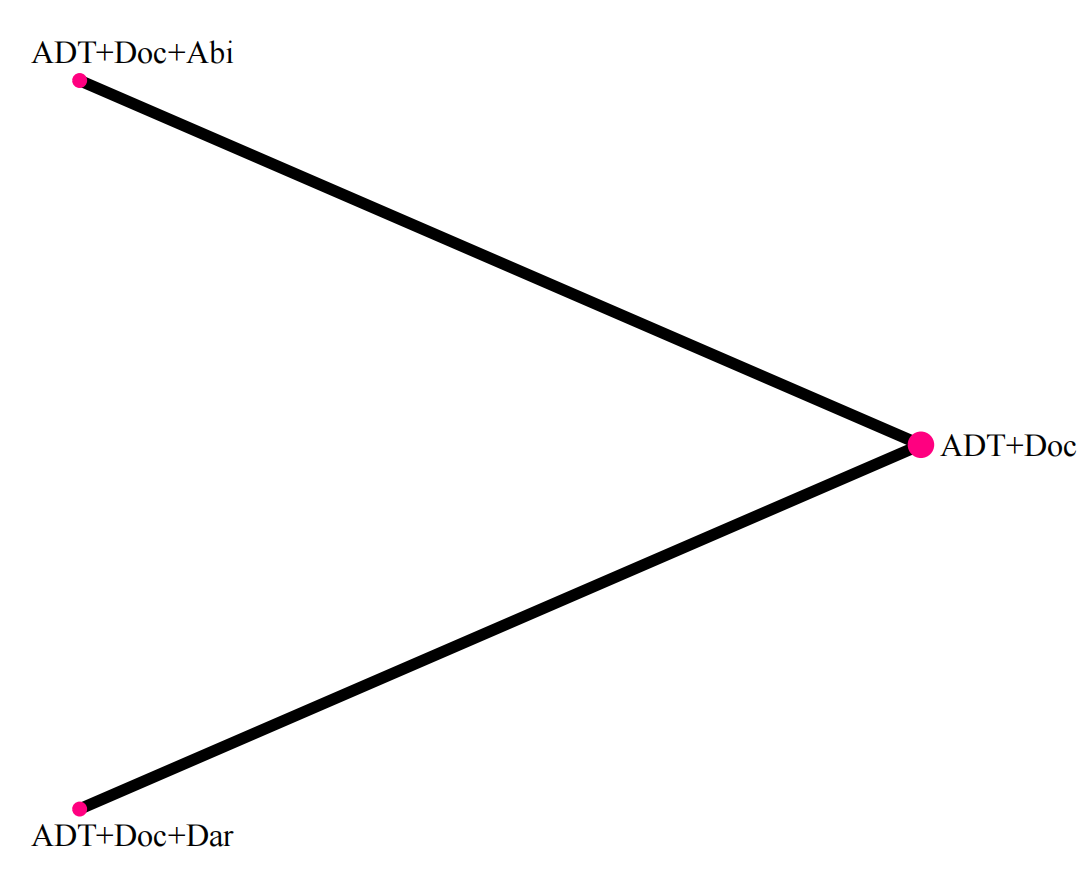


**Supplementary materials 6： Supplementary forest plots**

OS：overall survival；generalized PFS：generalized progression free survival；rPFS：radiographic progression free survival；PSA：Prostate-Specific Antigen；ADT：androgen deprivation treatment；Doc：docetaxel；Abi：abiraterone；Enz：enzalutamide；Apa：apalutamide；Dar：darolutamide；HV：high volume；LV：low volume；SAE：Serious adverse event.

A：OS for patients with ADT + Doc + ARSi concomitant (vs. ADT + Doc)


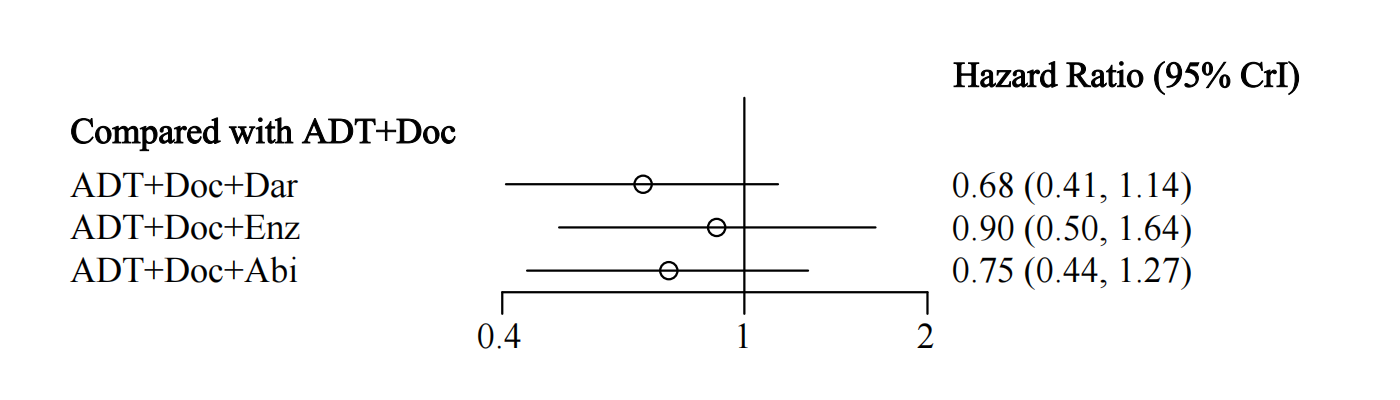


B：OS for patients with ADT + Doc + ARSi concomitant (vs. ADT + Doc + Dar)


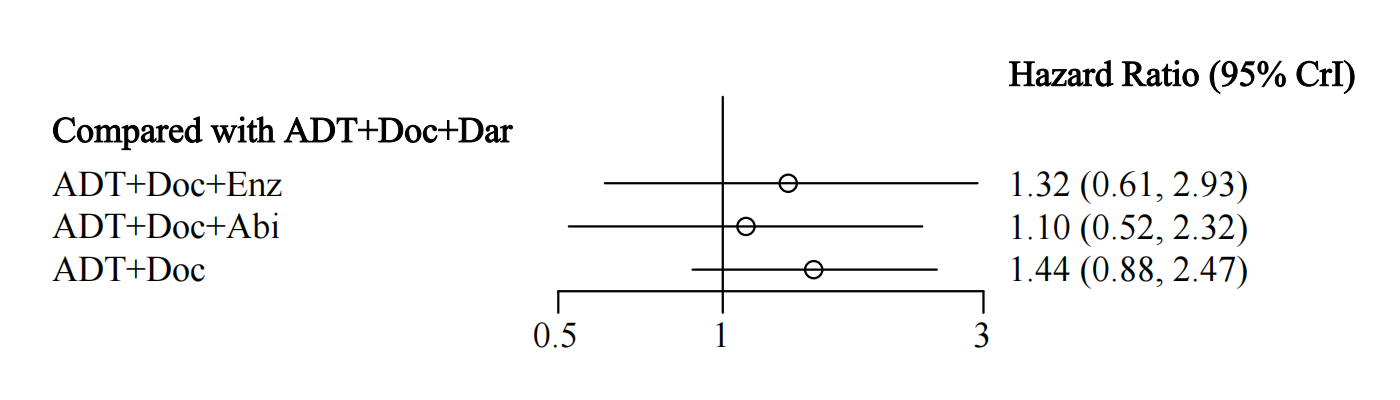


C：OS for patients with ADT + Doc + ARSi sequential (vs.ADT + Doc)


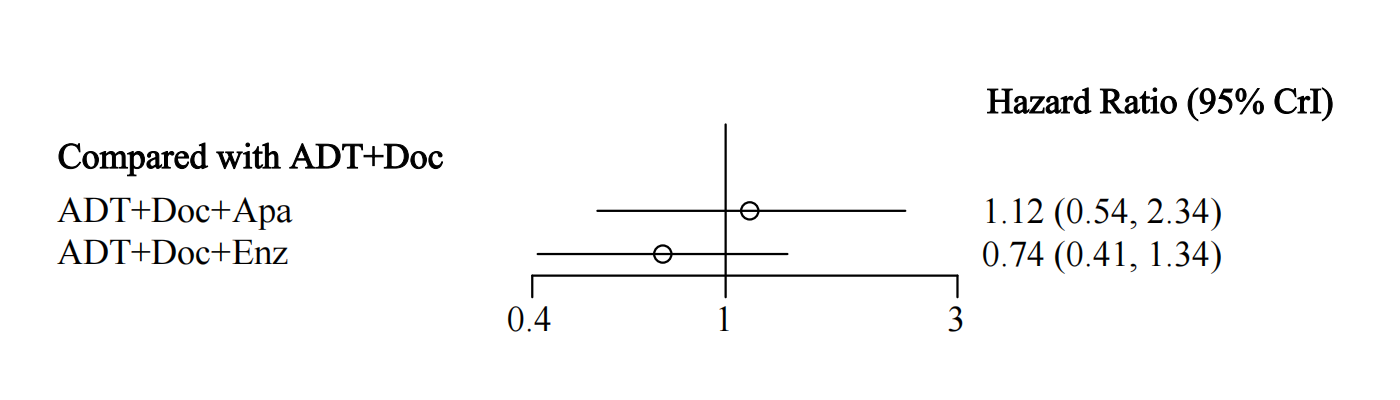


D：OS for patients with ADT + Doc + ARSi sequential (vs. ADT + Doc + Apa)


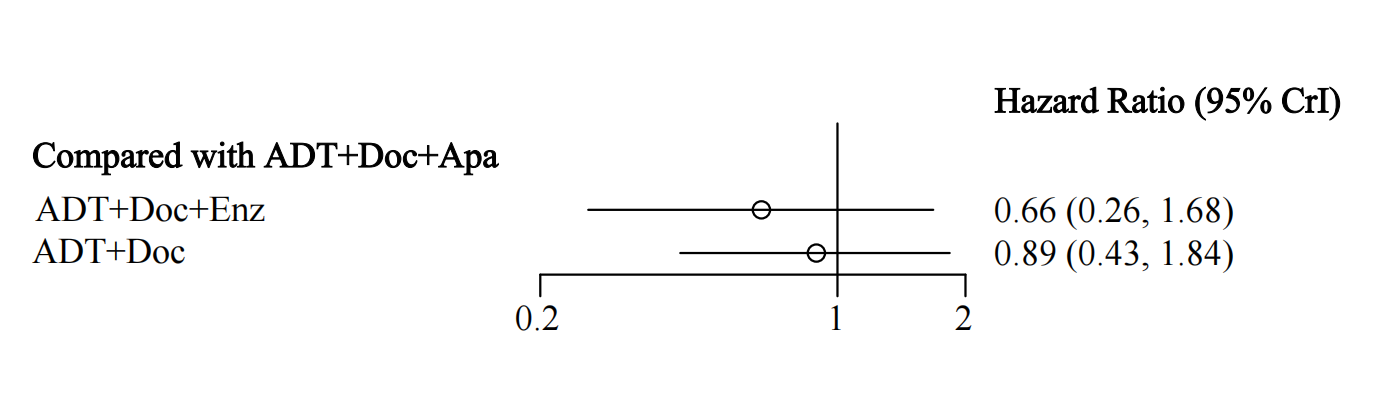


E：OS for patients with Low volume (vs. ADT + Doc)


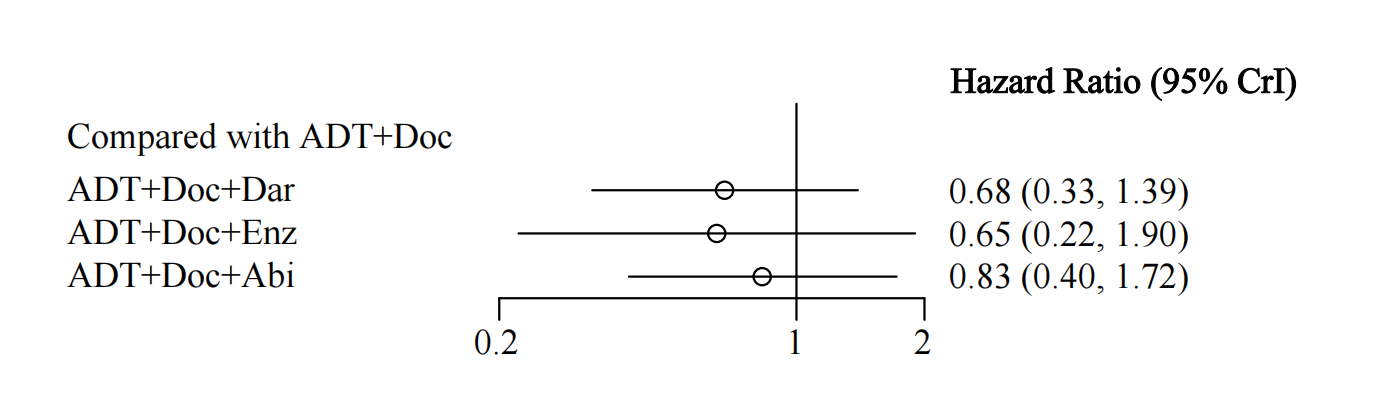


F：OS for patients with Low volume (vs. ADT + Doc + Enz)


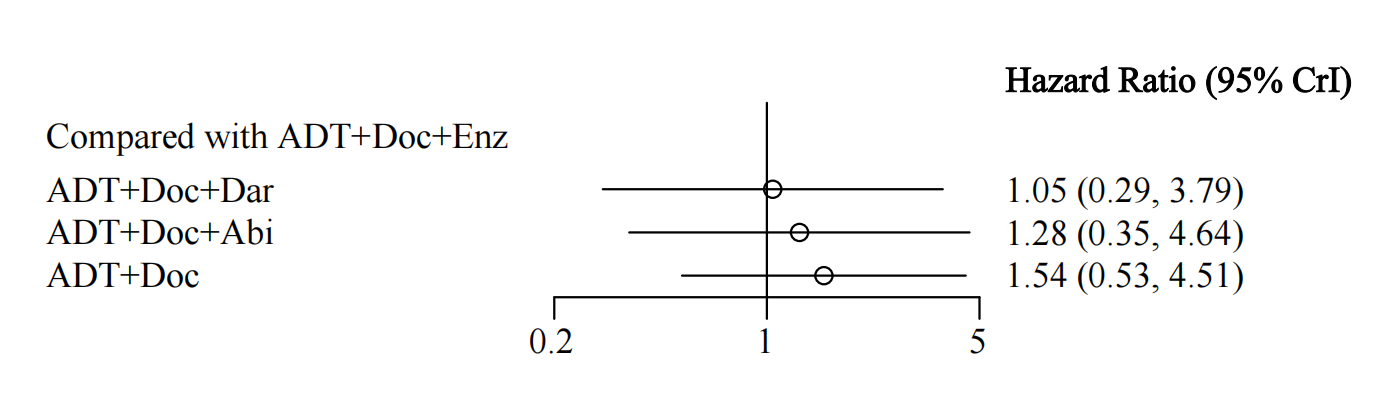


G：OS for patients with High volume (vs. ADT + Doc)


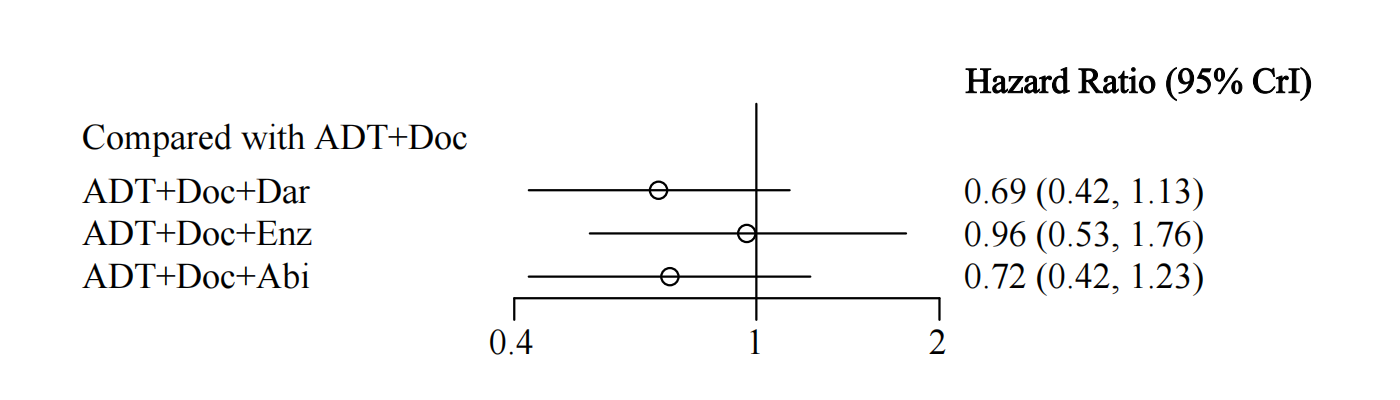


H：OS for patients with High volume (vs. ADT + Doc + Enz)


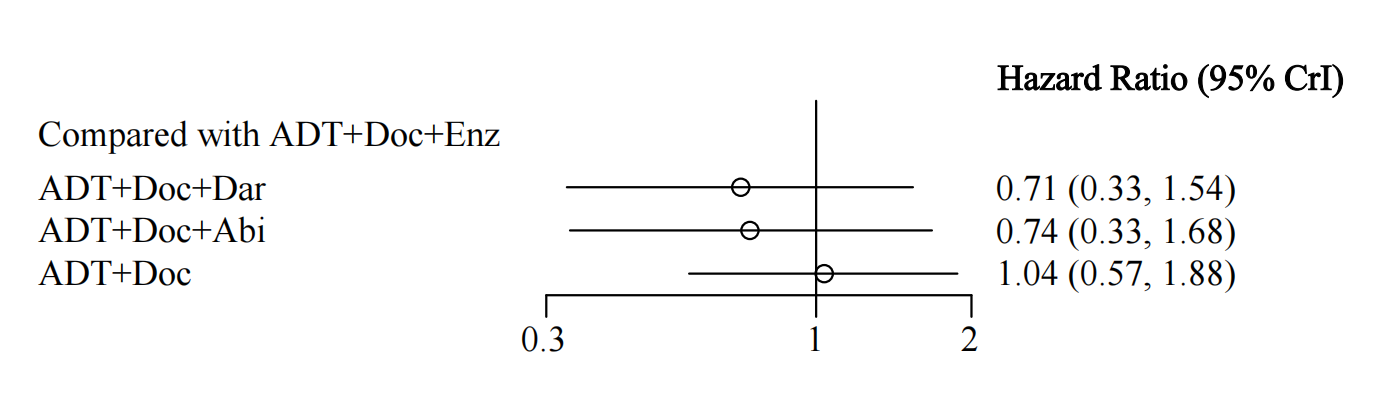


I：OS for patients with High volume (vs. ADT + Doc + Dar)


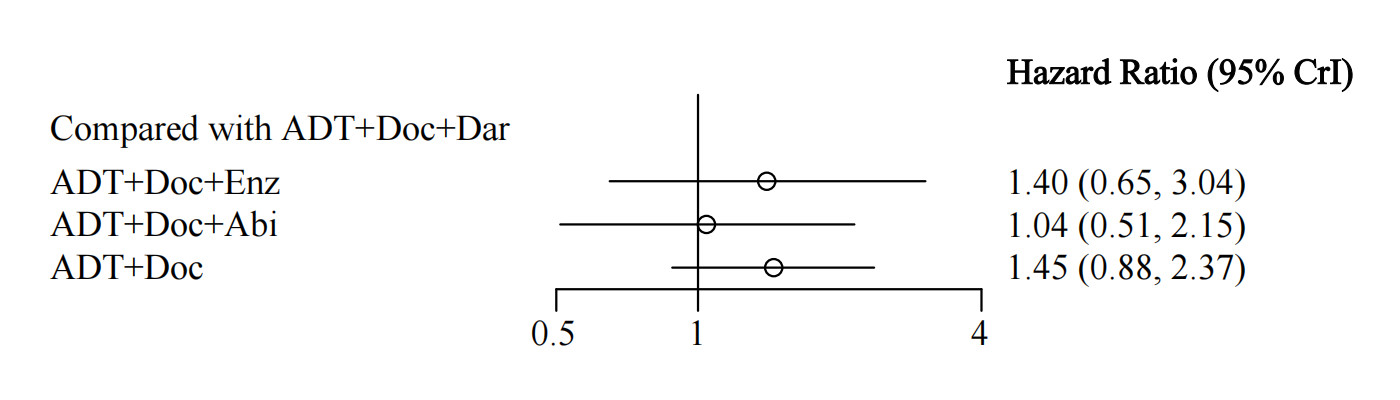


J：generalized PFS for patients with ADT + Doc + ARSi concomitant (vs. ADT + Doc)


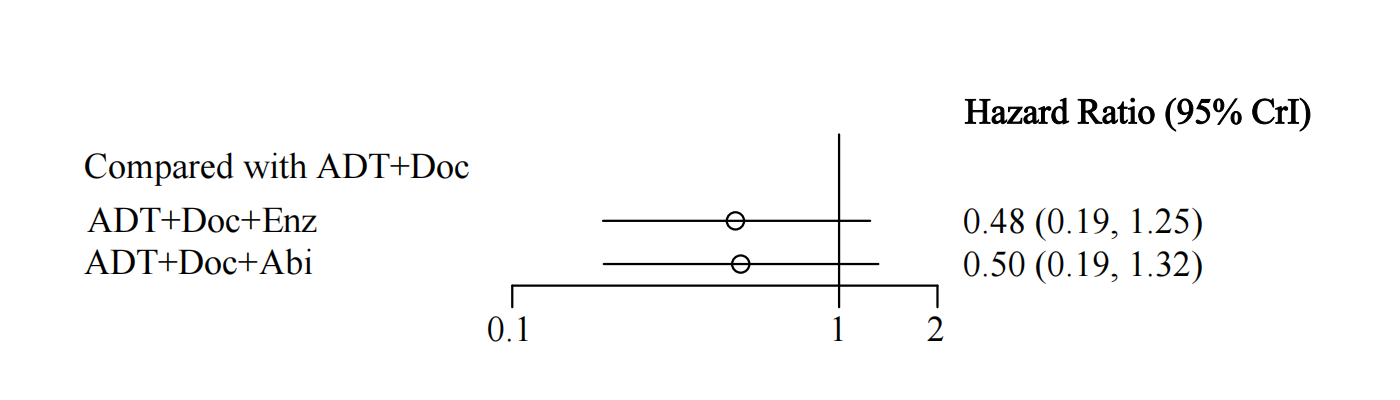


K：generalized PFS for patients with ADT + Doc + ARSi concomitant (vs. ADT + Doc + Enz)


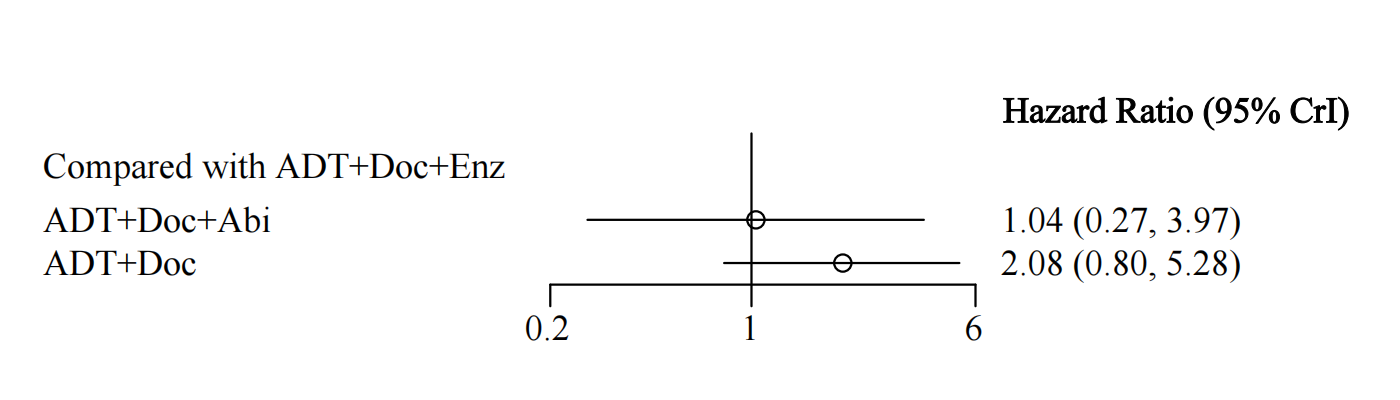


L：rPFS for patients with ADT + Doc + ARSi sequential (vs.ADT + Doc)


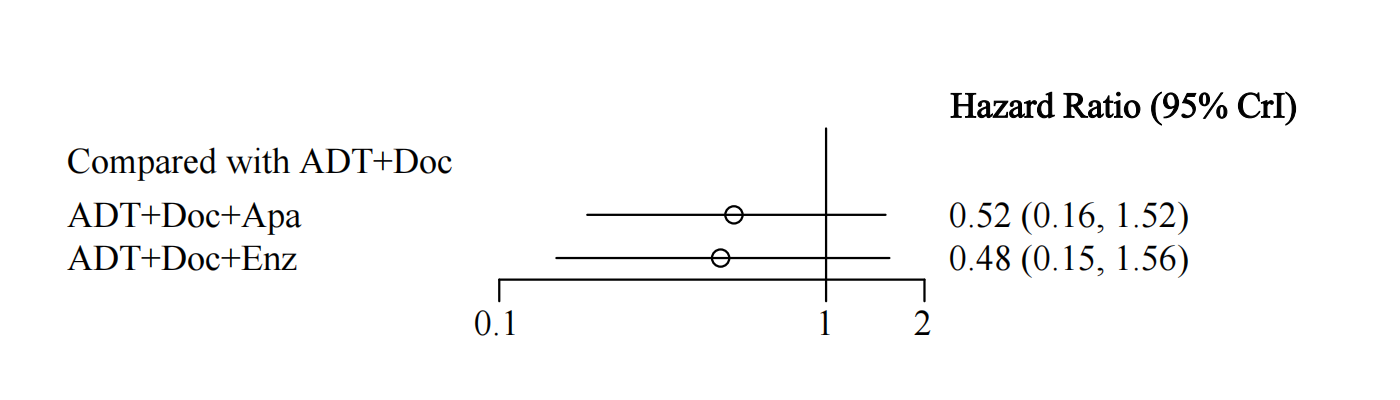


M：rPFS for patients with ADT + Doc + ARSi sequential (vs.ADT + Doc + Apa)


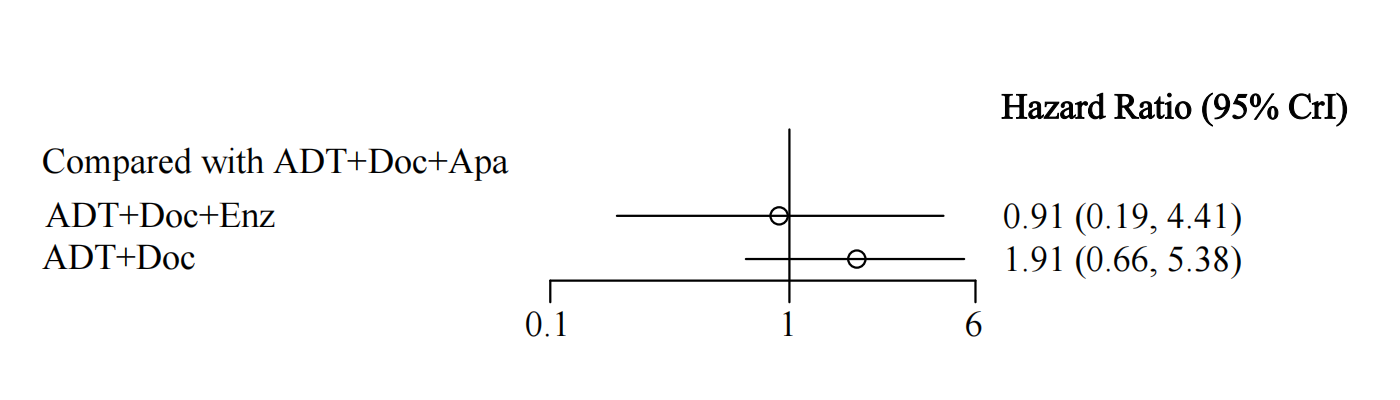


N：generalized PFS for patients with Low volume (vs. ADT + Doc)


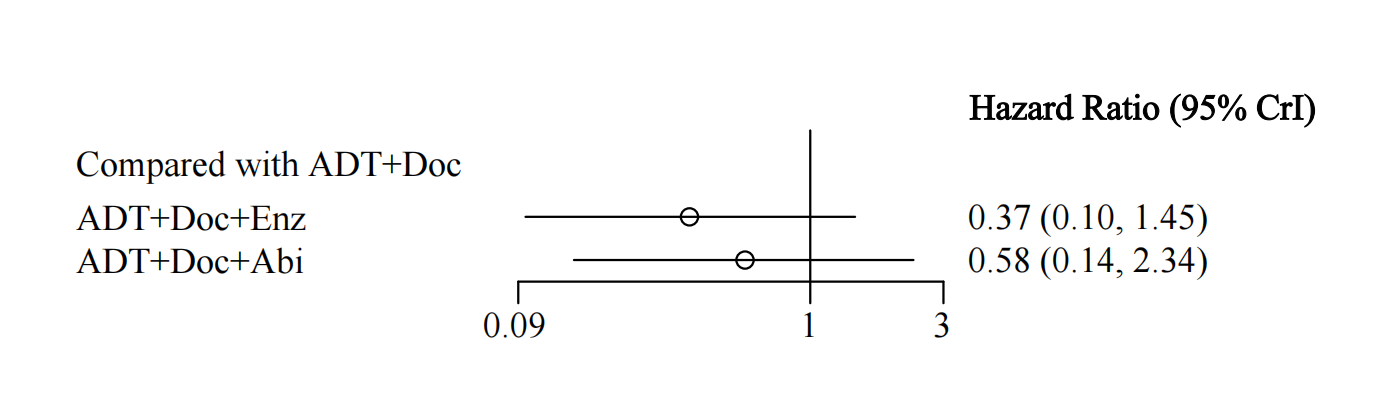


O：generalized PFS for patients with Low volume (vs. ADT + Doc + Enz)


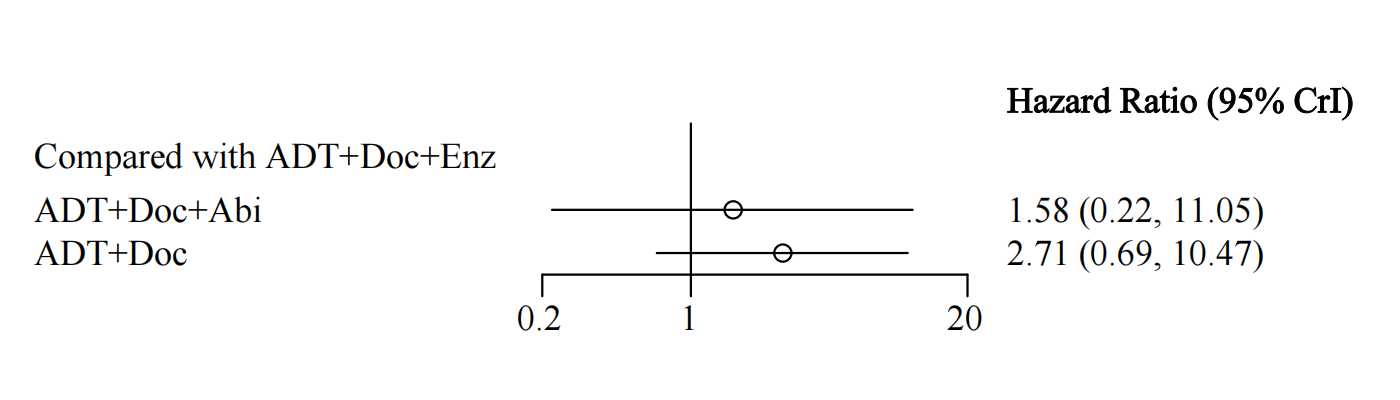


P：generalized PFS for patients with High volume (vs. ADT + Doc)


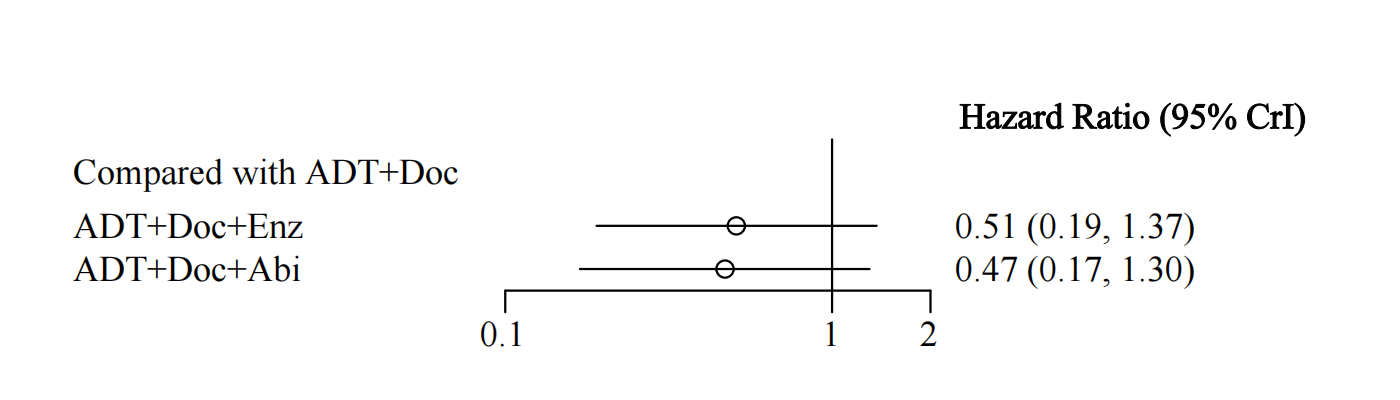


Q：generalized PFS for patients with High volume (vs. ADT + Doc + Enz)


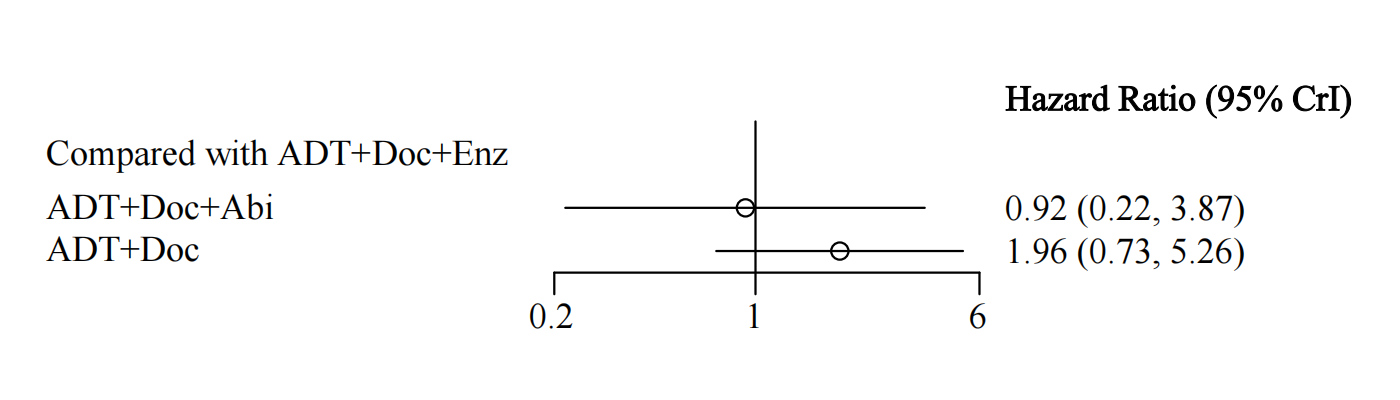


R：PSA (vs. ADT + Doc)


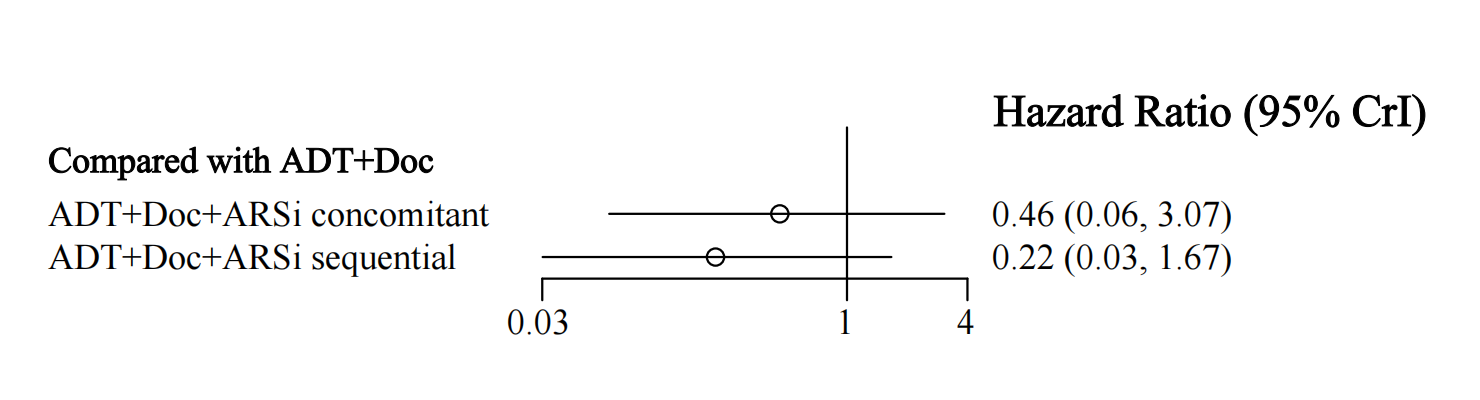


S：PSA (vs. ADT + Doc + ARSi concomitant)


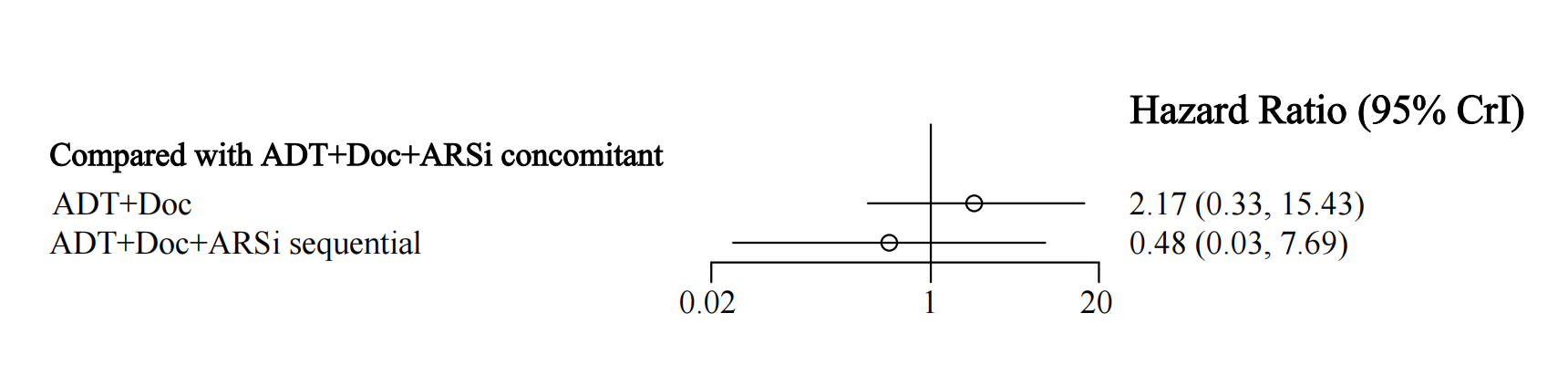


T：PSA (vs. ADT + Doc + ARSi sequential)


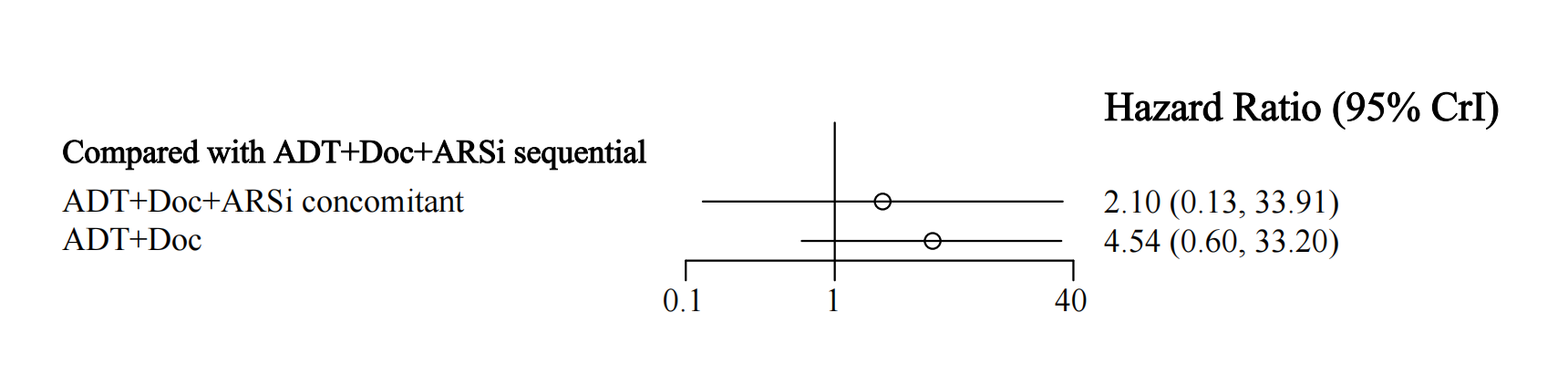


U：SAE (vs. ADT + Doc)


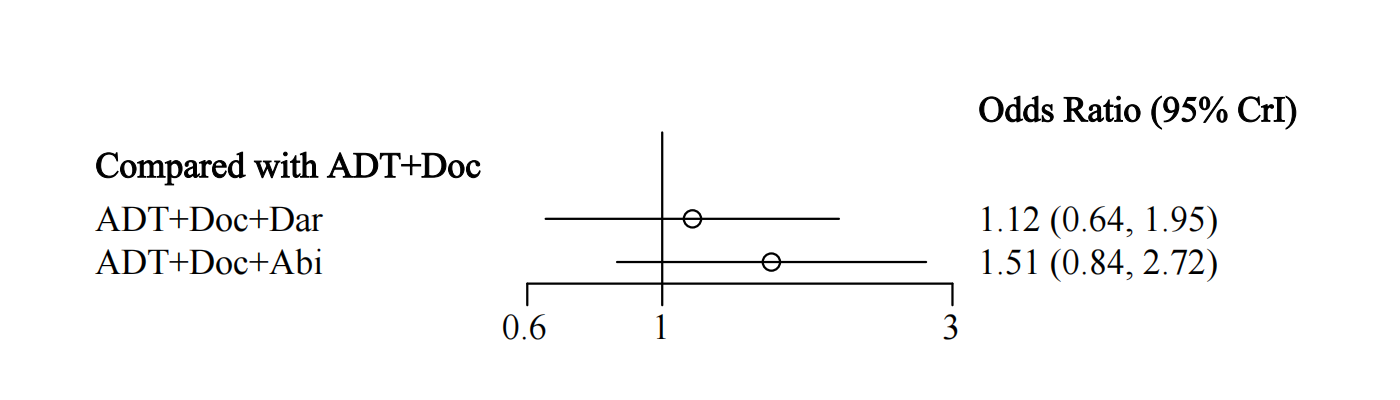


V：SAE (vs. ADT + Doc + Dar)


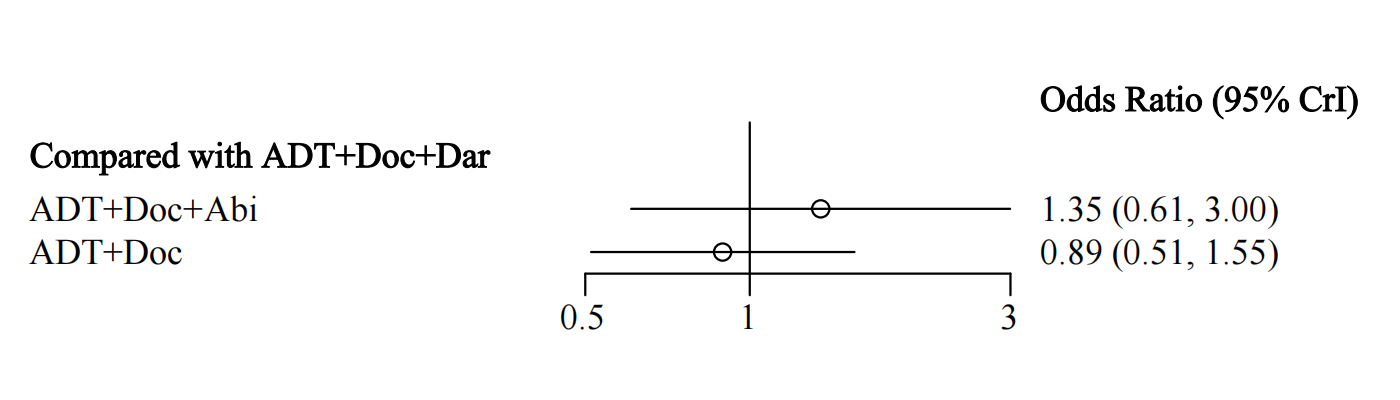


**Supplementary Material 7： Heterogeneity test**

ADT：androgen deprivation treatment；Doc：docetaxel；Abi：abiraterone；Enz：enzalutamide；Apa：apalutamide；Dar：darolutamide；OS：overall survival；generalized PFS：generalized progression free survival；NA：Not available.

Heterogeneity test for OS


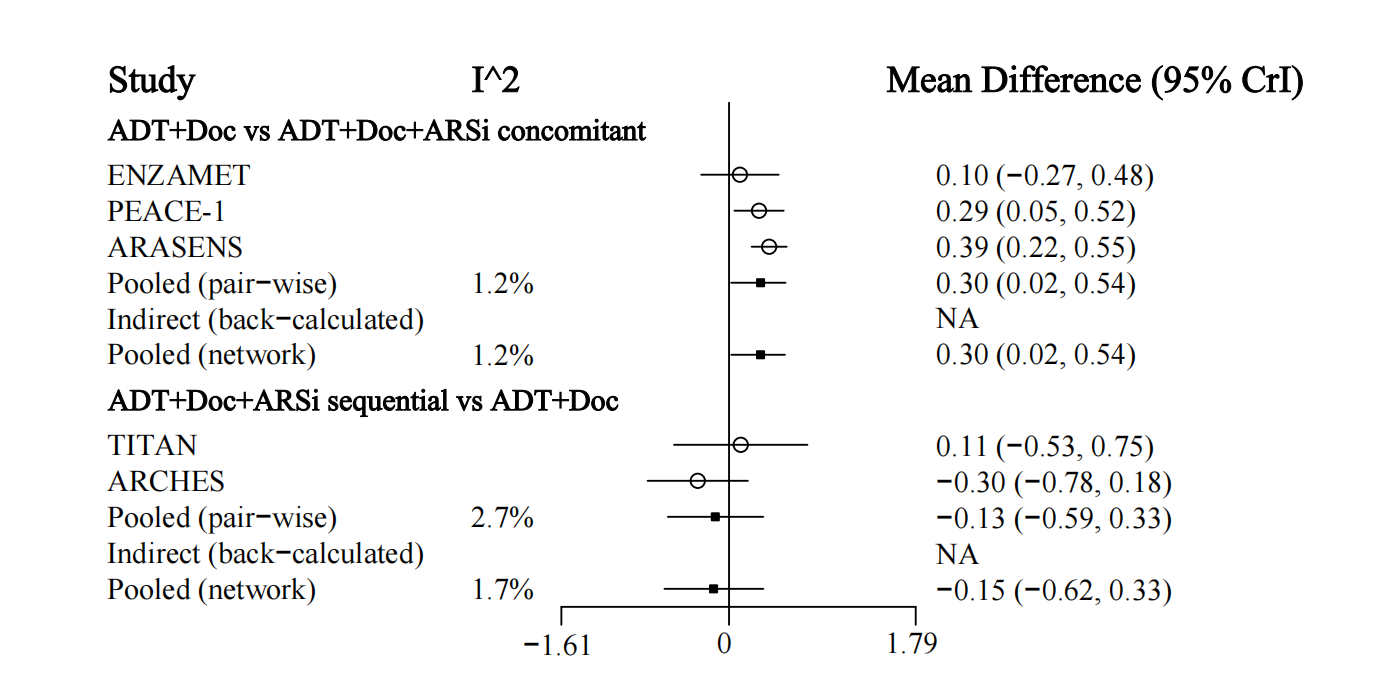


Heterogeneity test for generalized PFS


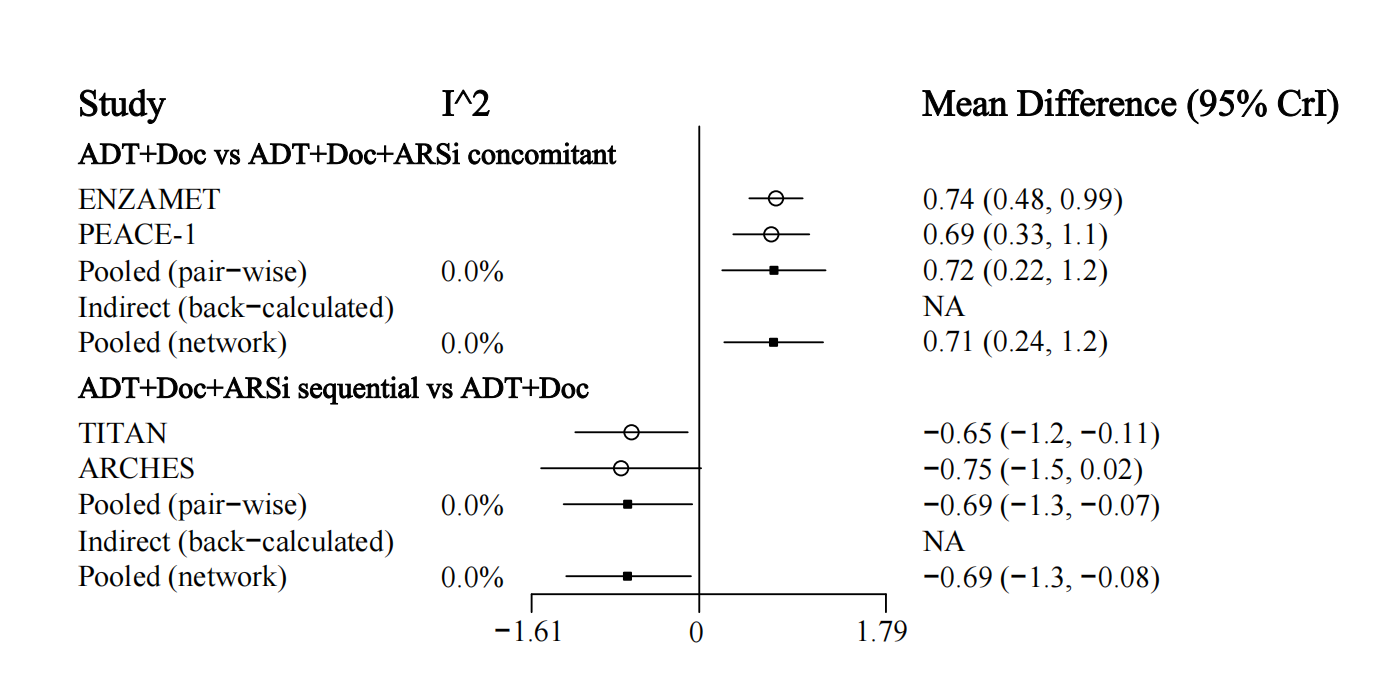


**Supplementary Material 8： Relative effect estimates for all possible treatment comparisons**

OS：overall survival；generalized PFS：generalized progression free survival；rPFS：radiographic progression free survival；PSA：Prostate-Specific Antigen；ADT：androgen deprivation treatment；Doc：docetaxel；Abi：abiraterone；Enz：enzalutamide；Apa：apalutamide；Dar：darolutamide；HV：high volume；LV：low volume；SAE：Serious adverse event.

OS, hazard ratio (95% CI)

| ADT + Doc + ARSi concomitant |  |  |
| --- | --- | --- |
| 0.74 (0.59, 0.98) | ADT + Doc |  |
| 0.85 (0.51, 1.46) | 1.15 (0.72, 1.82) | ADT + Doc + ARSi sequential |

OS (patients with ADT + Doc + ARSi concomitant), hazard ratio (95% CI)

| ADT + Doc + Dar |  |  |  |
| --- | --- | --- | --- |
| 0.76 (0.34, 1.64) | ADT + Doc + Enz |  |  |
| 0.91 (0.43, 1.91) | 1.19 (0.54, 2.65) | ADT + Doc + Abi |  |
| 0.68 (0.41, 1.14) | 0.90 (0.50, 1.64) | 0.75 (0.44, 1.27) | ADT + Doc |

OS (patients with ADT + Doc + ARSi sequential), hazard ratio (95% CI)

| ADT + Doc + Apa |  |  |
| --- | --- | --- |
| 1.51 (0.60, 3.86) | ADT + Doc + Enz |  |
| 1.12 (0.54, 2.34) | 0.74 (0.41, 1.34) | ADT + Doc |

OS (HV patients with ADT + Doc + ARSi concomitant), hazard ratio (95% CI)

| ADT + Doc + Dar |  |  |  |
| --- | --- | --- | --- |
| 0.71 (0.33, 1.54) | ADT + Doc + Enz |  |  |
| 0.96 (0.47, 1.96) | 1.34 (0.60, 3.00) | ADT + Doc + Abi |  |
| 0.69 (0.42, 1.13) | 0.96 (0.53, 1.76) | 0.72 (0.42, 1.23) | ADT + Doc |

OS (LV patients with ADT + Doc + ARSi concomitant), hazard ratio (95% CI)

| ADT + Doc + Dar |  |  |  |
| --- | --- | --- | --- |
| 1.05 (0.29, 3.79) | ADT + Doc + Enz |  |  |
| 0.81 (0.29, 2.25) | 0.78 (0.22, 2.83) | ADT + Doc + Abi |  |
| 0.68 (0.33, 1.39) | 0.65 (0.22, 1.90) | 0.83 (0.40, 1.72) | ADT + Doc |

generalized PFS, hazard ratio (95% CI)

| ADT + Doc + ARSi concomitant |  |  |
| --- | --- | --- |
| 0.49 (0.30, 0.80) | ADT + Doc |  |
| 0.97 (0.45, 2.12) | 1.99 (1.10, 3.7) | ADT + Doc + ARSi sequential |

generalized PFS (patients with ADT + Doc + ARSi concomitant), hazard ratio (95% CI)

| ADT + Doc + Enz |  |  |
| --- | --- | --- |
| 0.97 (0.25, 3.71) | ADT + Doc + Abi |  |
| 0.48 (0.19, 1.24) | 0.50 (0.19, 1.32) | ADT + Doc |

rPFS (patients with ADT + Doc + ARSi sequential), hazard ratio (95% CI)

| ADT + Doc + Enz |  |  |
| --- | --- | --- |
| 1.10 (0.23, 5.27) | ADT + Doc + Apa |  |
| 0.52 (0.19, 1.52) | 0.48 (0.15, 1.56) | ADT + Doc |

generalized PFS (HV patients with ADT + Doc + ARSi concomitant), hazard ratio (95% CI)

| ADT + Doc + Enz |  |  |
| --- | --- | --- |
| 1.08 (0.26, 4.58) | ADT + Doc + Abi |  |
| 0.51 (0.19, 1.37) | 0.47 (0.17, 1.30) | ADT + Doc |

generalized PFS (LV patients with ADT + Doc + ARSi concomitant), hazard ratio (95% CI)

| ADT + Doc + Enz |  |  |
| --- | --- | --- |
| 0.63 (0.09, 4.50) | ADT + Doc + Abi |  |
| 0.37 (0.10, 1.45) | 0.58 (0.14, 2.34) | ADT + Doc |

PSA, hazard ratio (95% CI)

| ADT + Doc + ARSi concomitant |  |  |
| --- | --- | --- |
| 0.46 (0.06, 3.07) | ADT + Doc |  |
| 2.10 (0.13, 33.91) | 4.54 (0.60, 33.20) | ADT + Doc + ARSi sequential |

SAE (patients with ADT + Doc + ARSi concomitant), hazard ratio (95% CI)

| ADT + Doc + Dar |  |  |
| --- | --- | --- |
| 0.74 (0.33, 1.65) | ADT + Doc + Abi |  |
| 1.12 (0.64, 1.95) | 1.51 (0.84, 2.72) | ADT + Doc |

**Supplementary Material 9： OS/rPFS ranking and ranking probability**

OS：overall survival；generalized PFS：generalized progression free survival；rPFS：radiographic progression free survival；PSA：Prostate-Specific Antigen；ADT：androgen deprivation treatment；Doc：docetaxel；Abi：abiraterone；Enz：enzalutamide；Apa：apalutamide；Dar：darolutamide；HV：high volume；LV：low volume；SAE：Serious adverse event.

A. OS for all patients


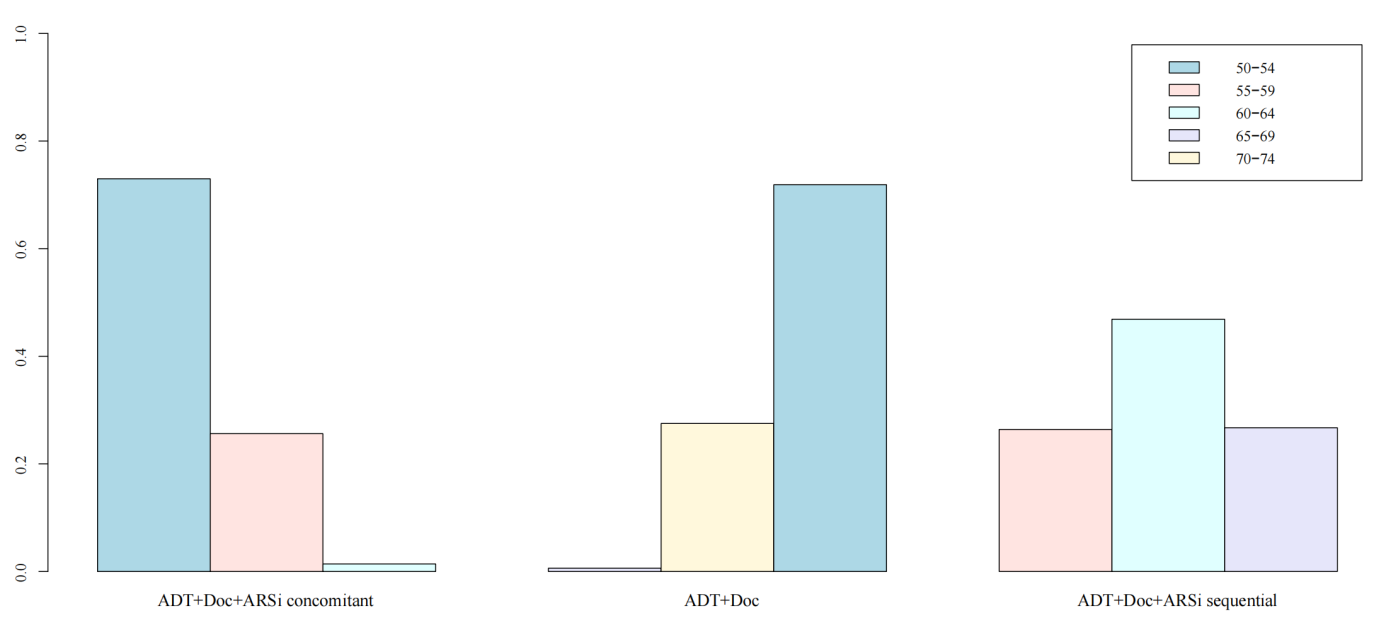


B. OS for patients with ADT + Doc + ARSi concomitant


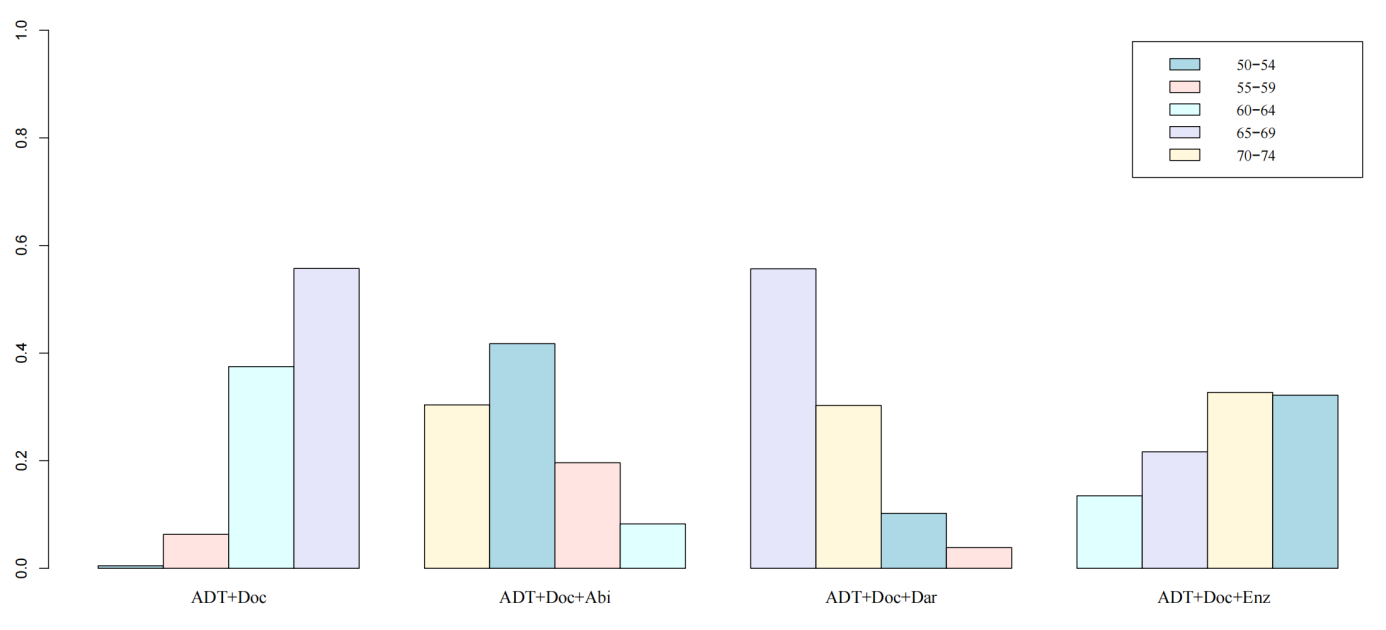


C. OS for patients with ADT + Doc + ARSi sequential


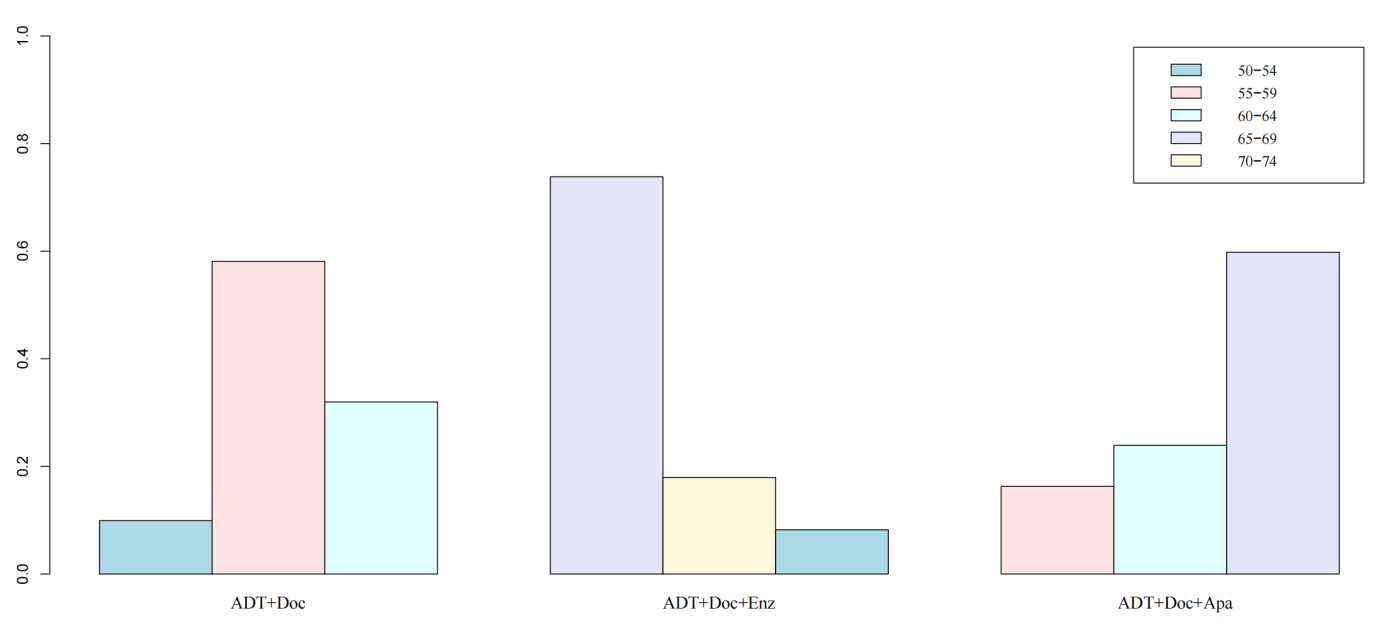


D. OS for HV patients with ADT + Doc + ARSi concomitant


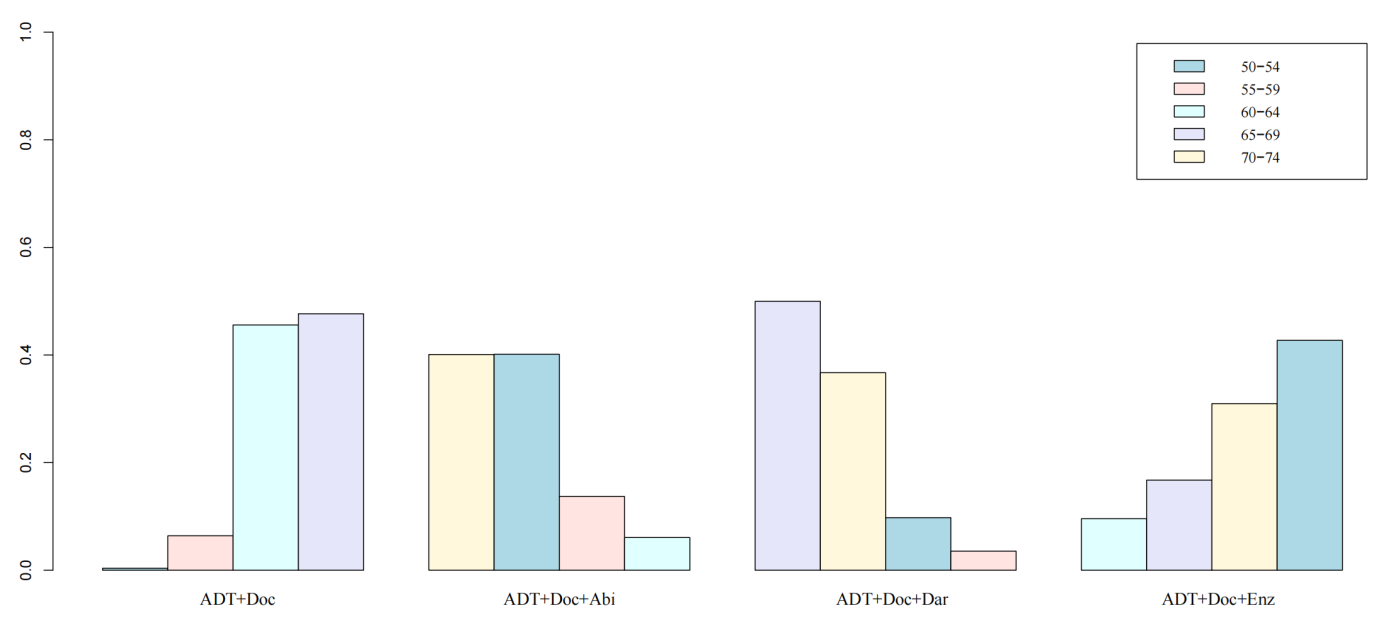


E. OS for LV patients with ADT + Doc + ARSi concomitant


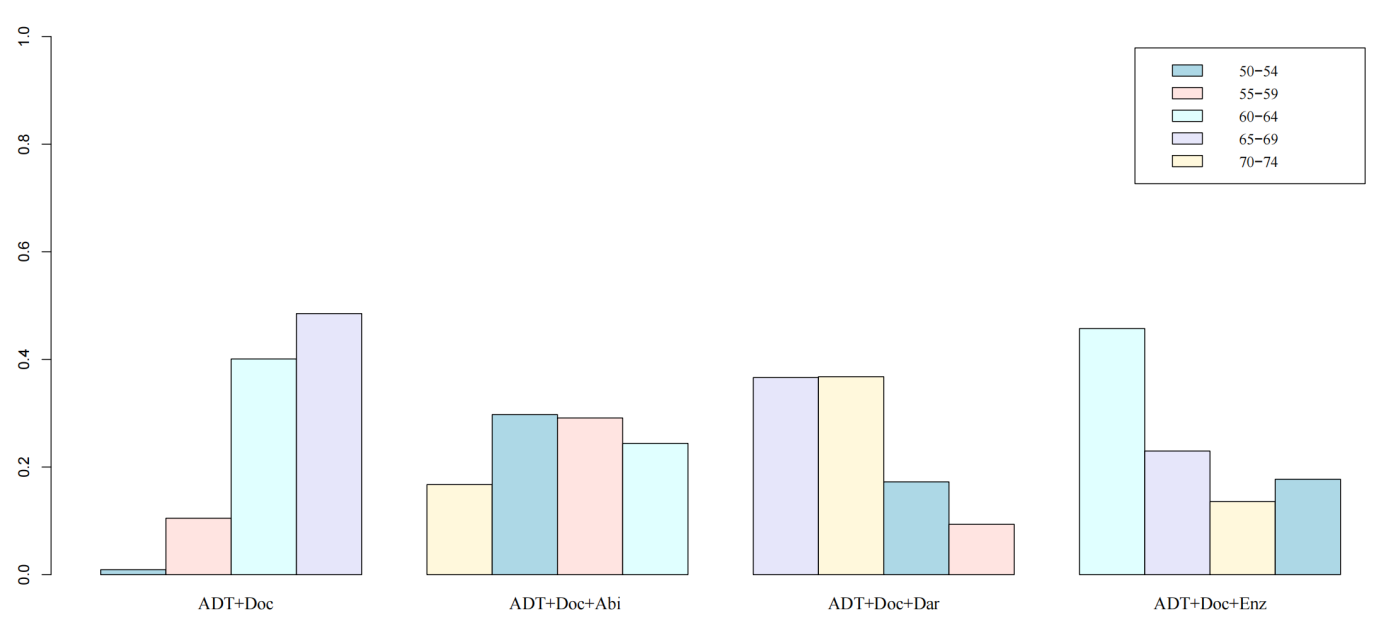


F. generalized PFS for all patients


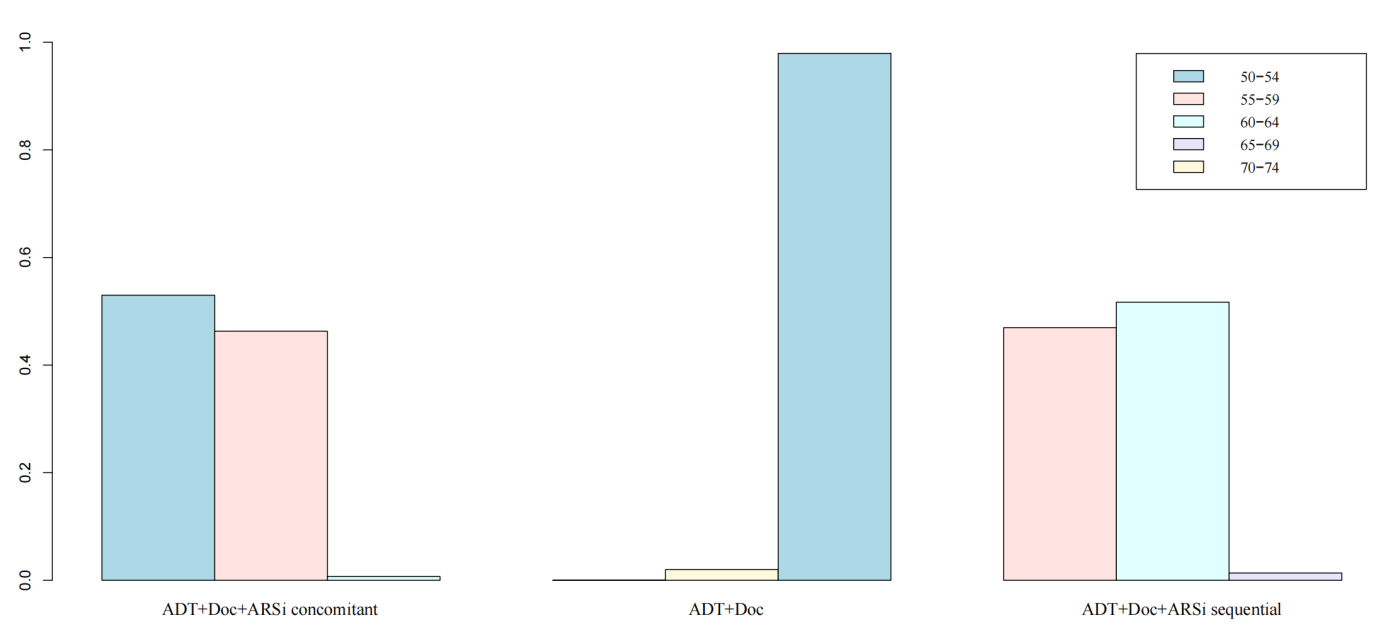


G. generalized PFS for patients with ADT + Doc ARSi concomitant


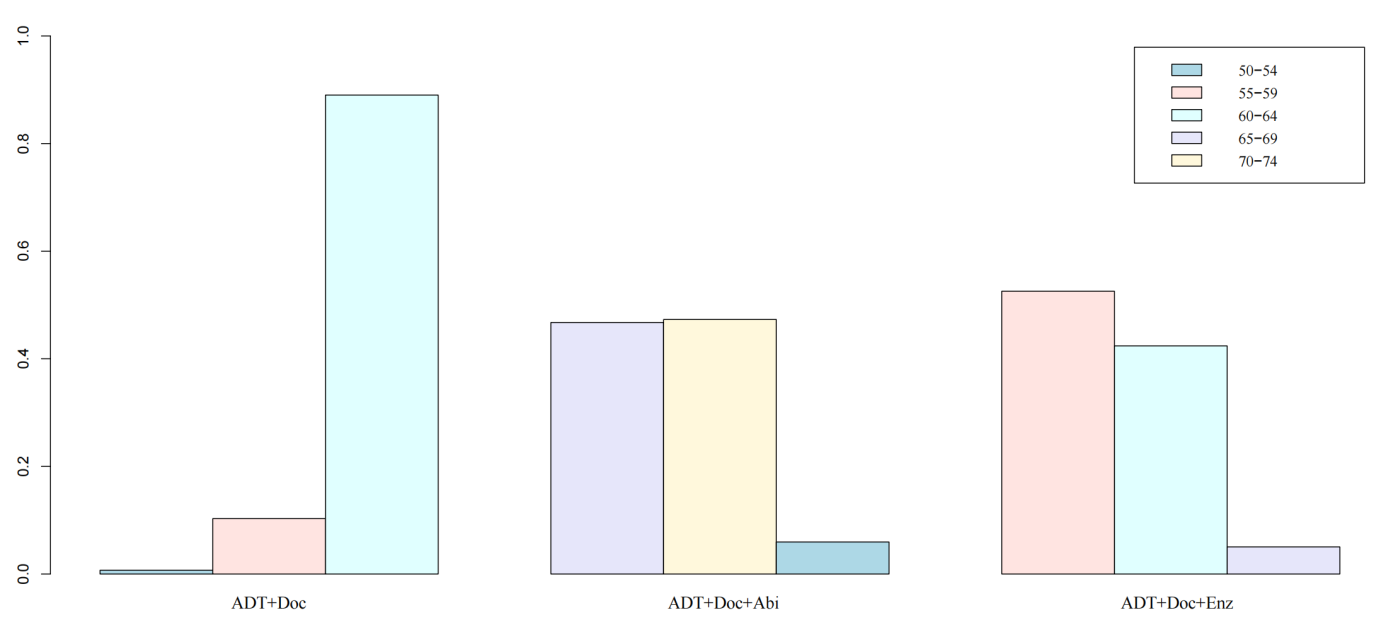


H. rPFS for patients with ADT + Doc + ARSi sequential


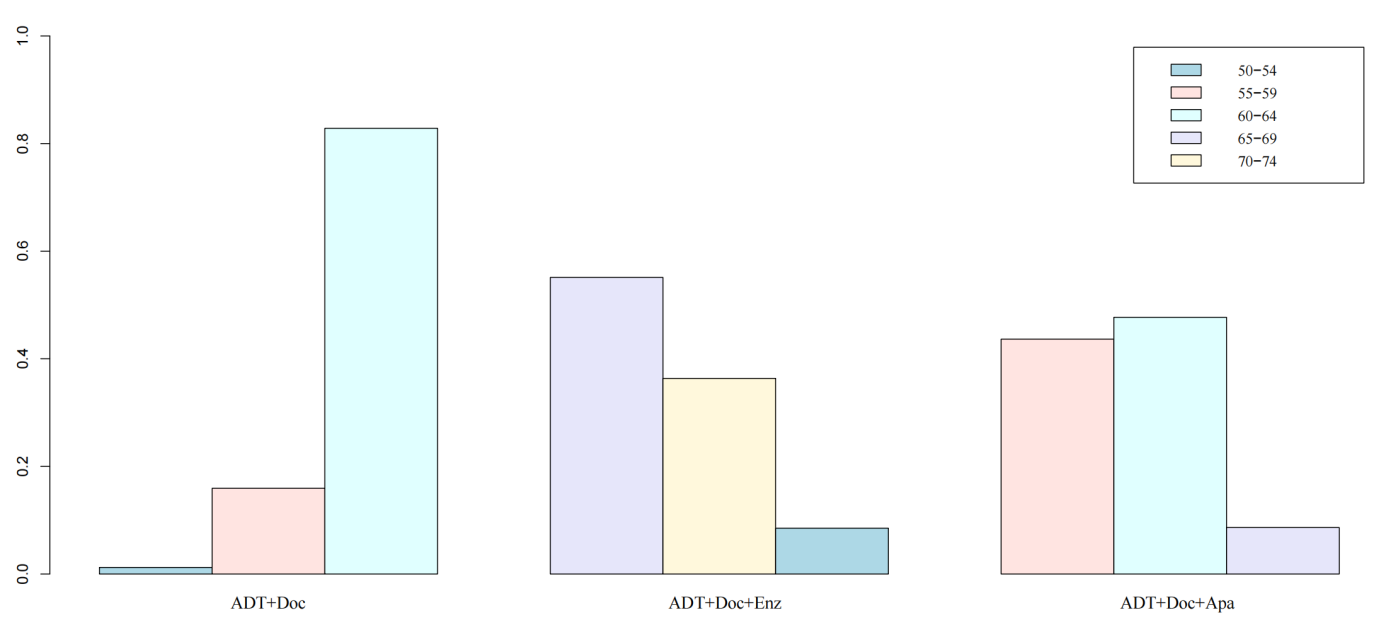


I： generalized PFSfor HV patients with ADT + Doc + ARSi concomitant


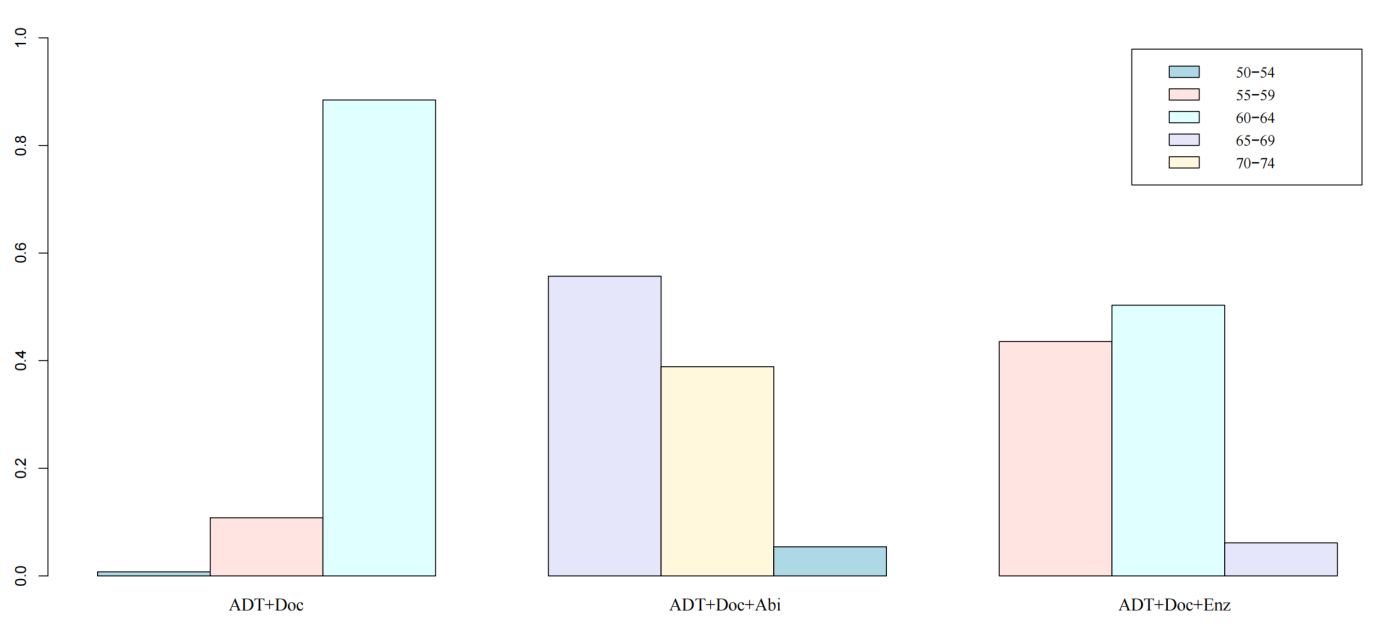


J： generalized PFS for LV patients with ADT + Doc + ARSi concomitant


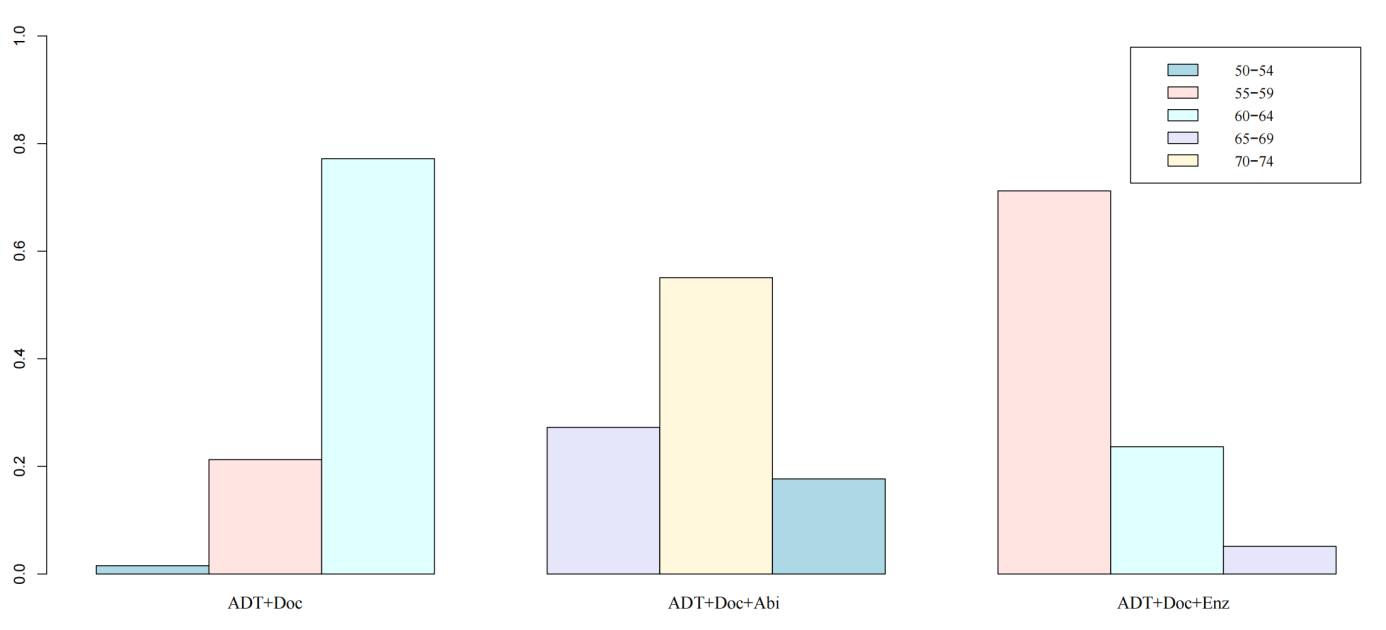


K： PSA for all patients


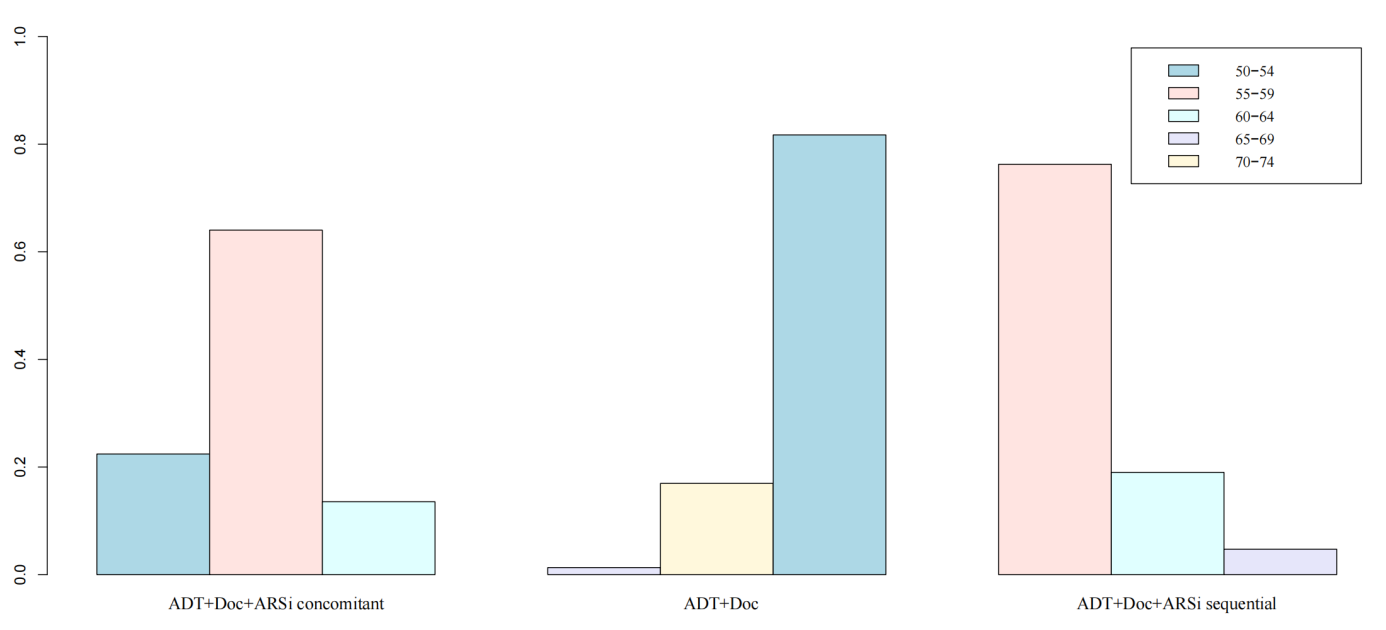


L： SAE


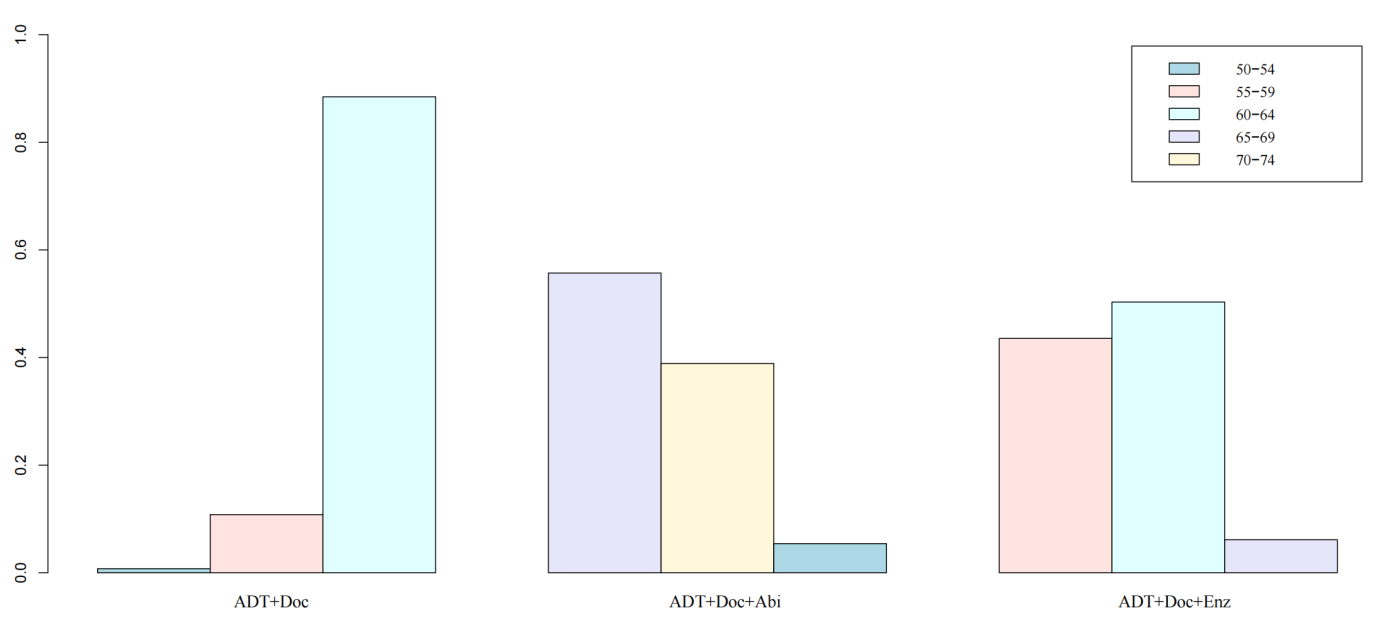


**Supplementary Material 10： Trace and density plots**

OS：overall survival；generalized PFS：generalized progression free survival；rPFS：radiographic progression free survival；PSA：Prostate-Specific Antigen；ADT：androgen deprivation treatment；Doc：docetaxel；Abi：abiraterone；Enz：enzalutamide；Apa：apalutamide；Dar：darolutamide；HV：high volume；LV：low volume；SAE：Serious adverse event.

A：Trace and density plots for OS


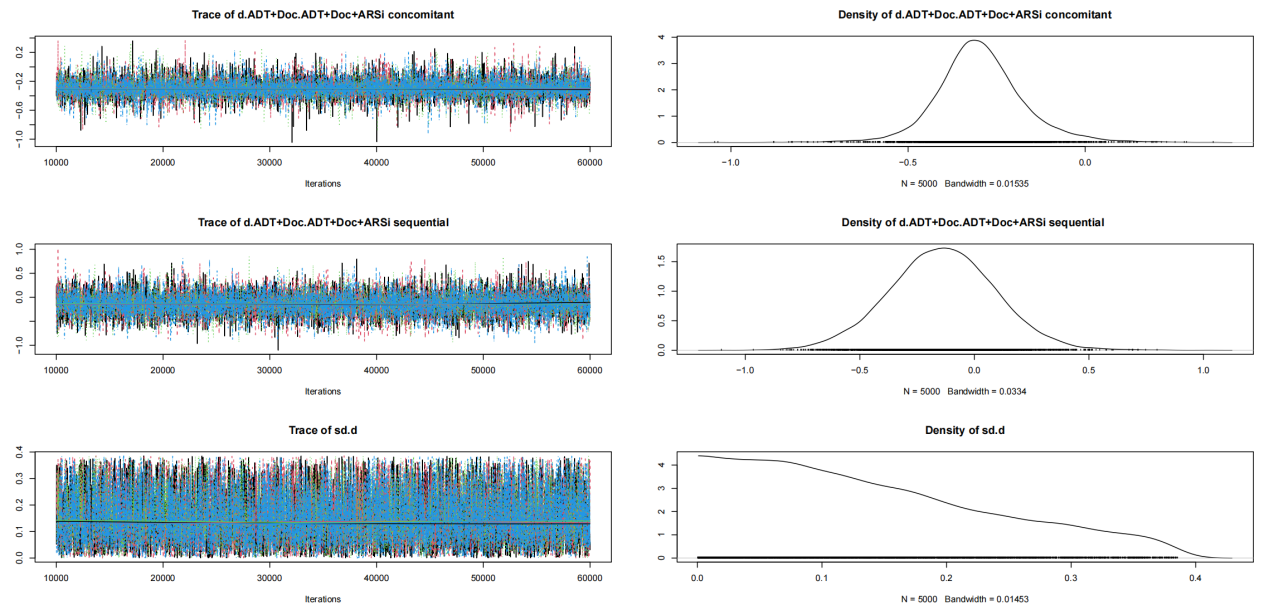


B：Trace and density plots for OS (patients with ADT + Doc + ARSi concomitant)


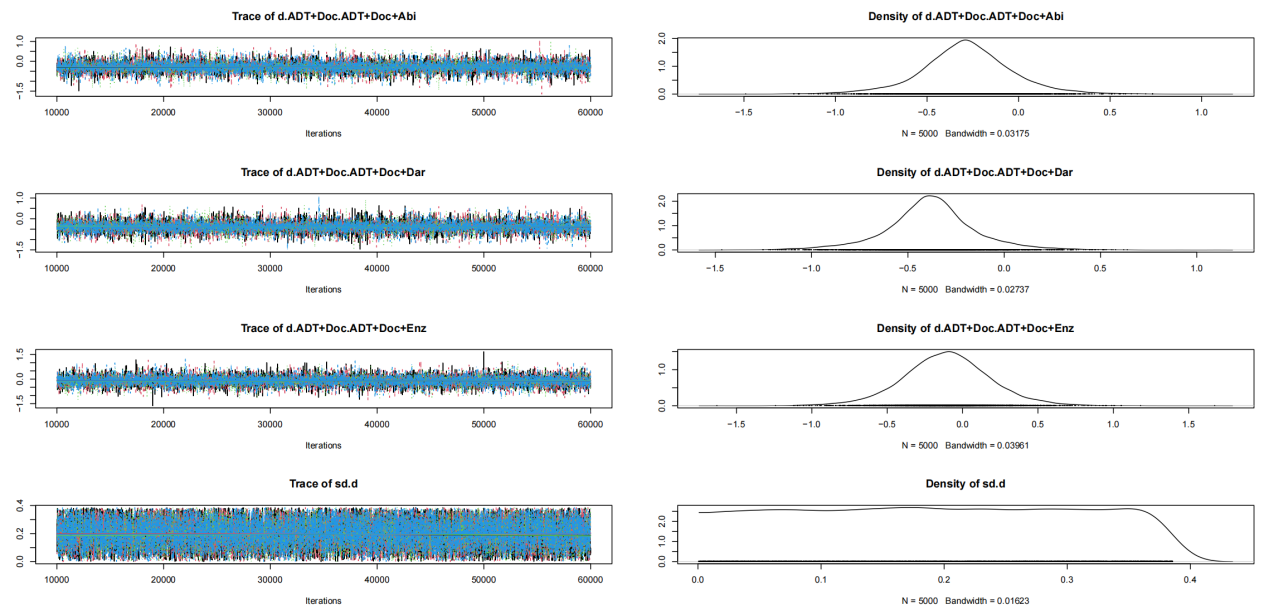


C：Trace and density plots for OS (patients with ADT + Doc + ARSi sequential)


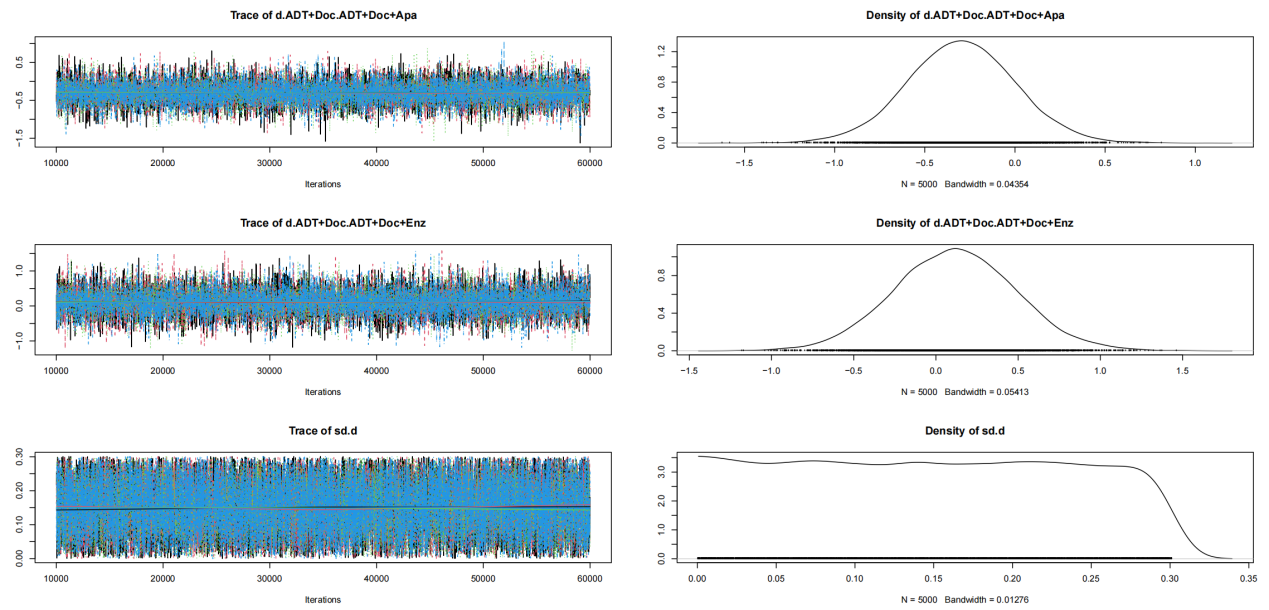


D：Trace and density plots for OS (HV patients with ADT + Doc + ARSi concomitant)


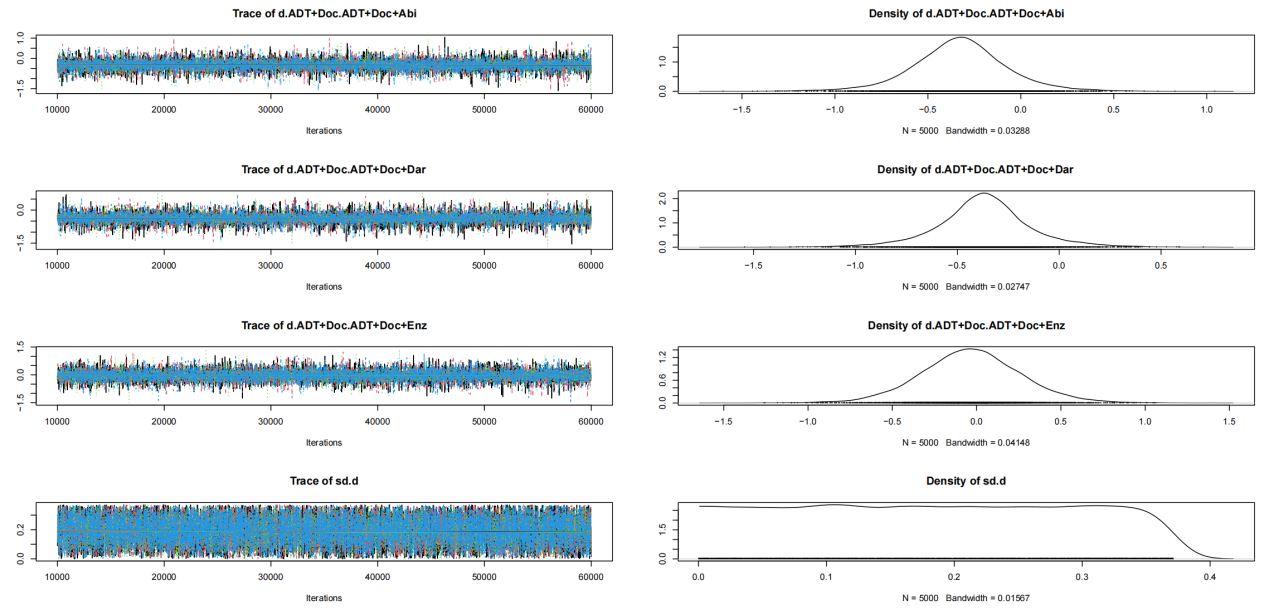


E：Trace and density plots for OS (LV patients with ADT + Doc + ARSi concomitant)


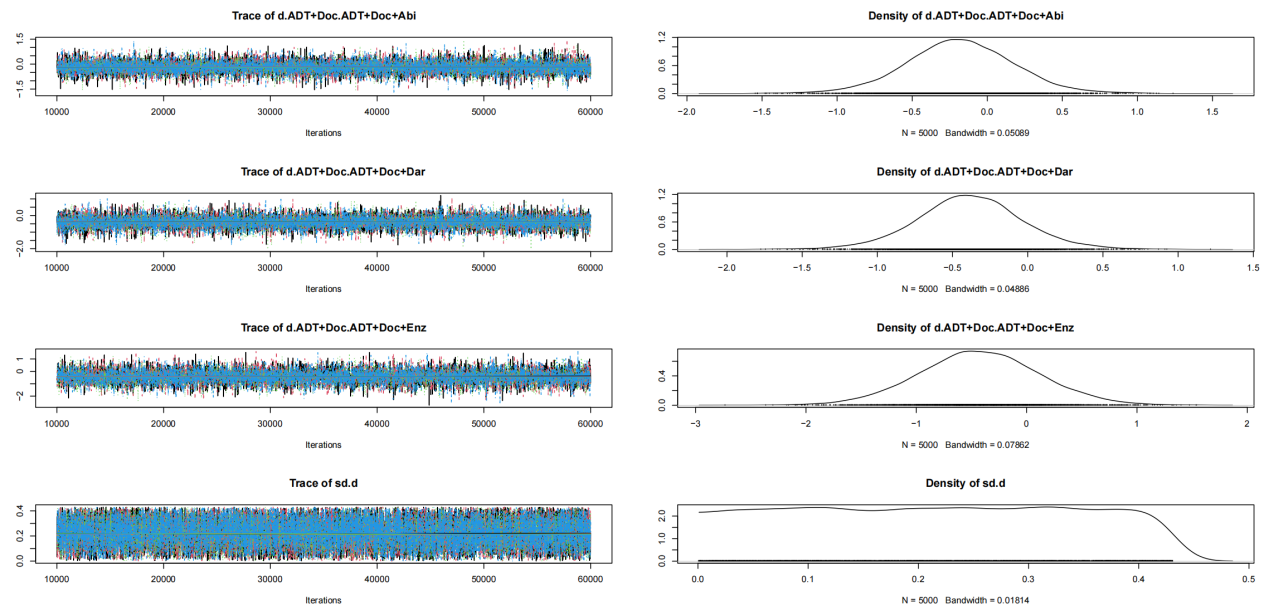


F：Trace and density plots for generalized PFS


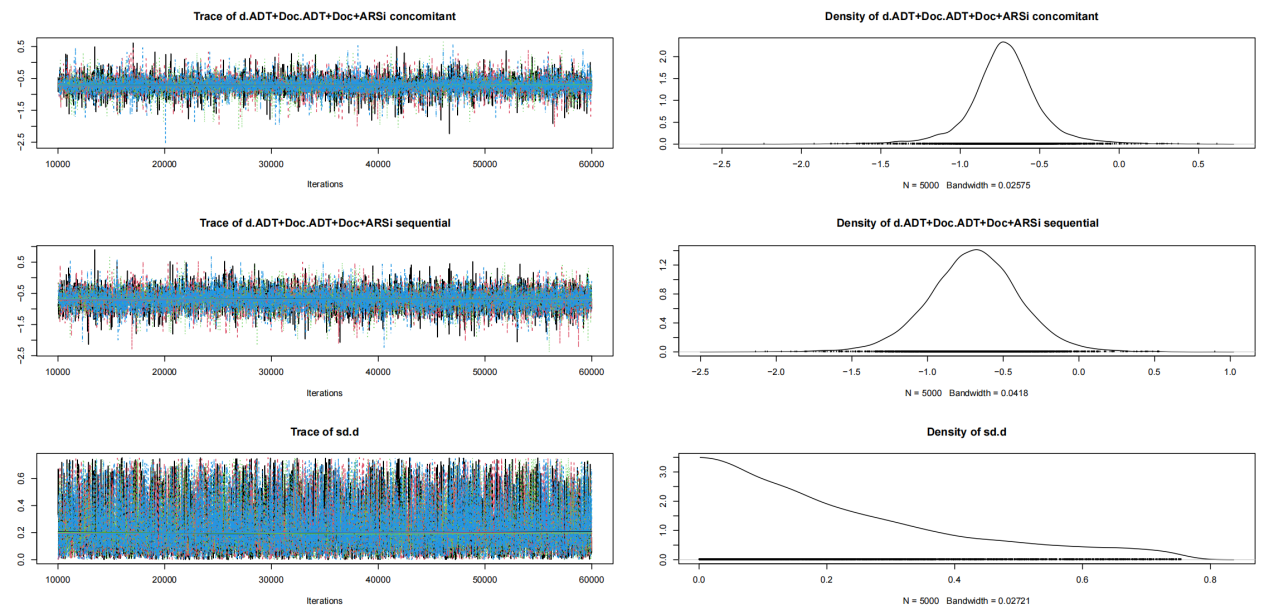


G：Trace and density plots for generalized PFS (patients with ADT + Doc + ARSi concomitant)


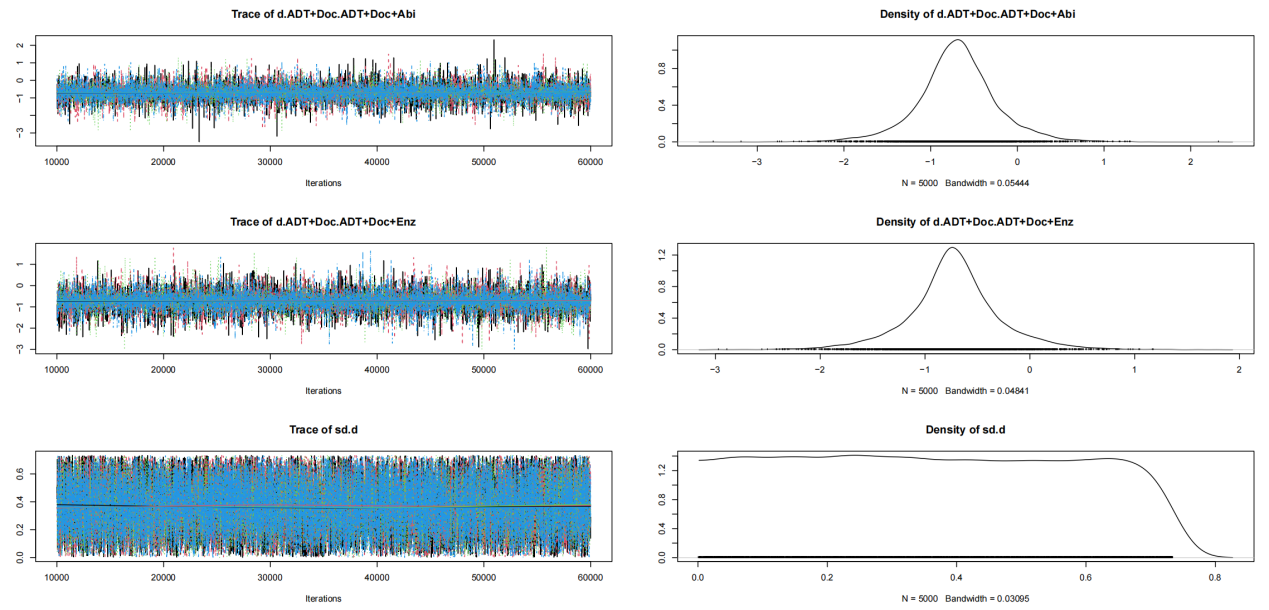


H：Trace and density plots for rPFS (patients with ADT + Doc + ARSi sequential)


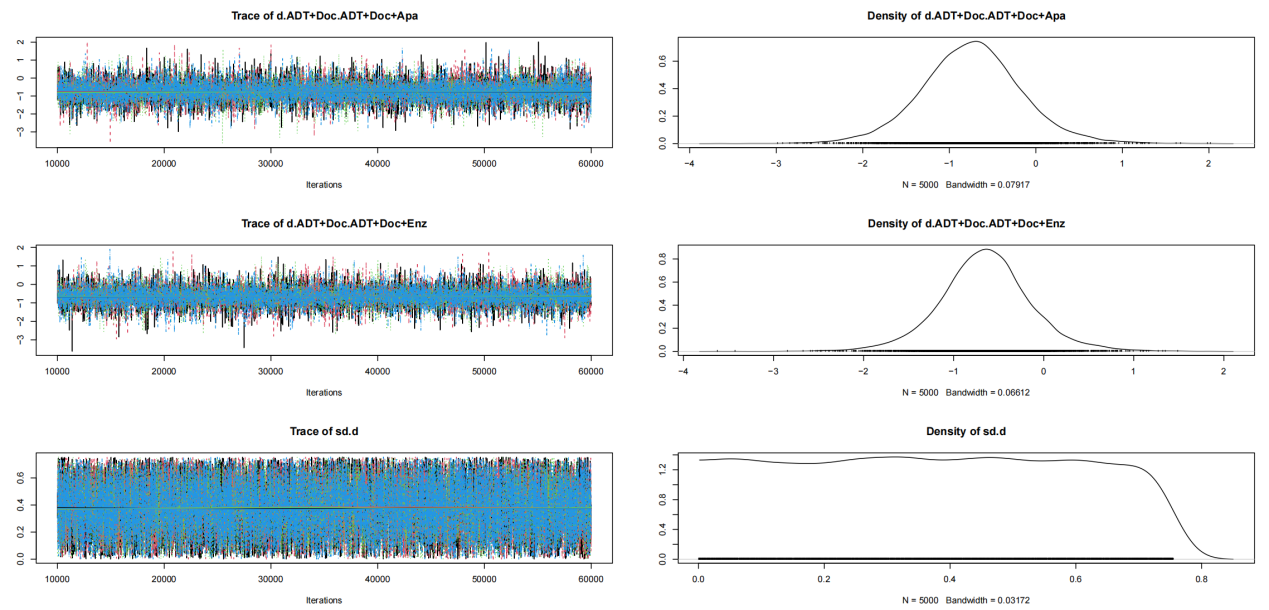


I：Trace and density plots for generalized PFS (HV patients with ADT + Doc + ARSi concomitant)


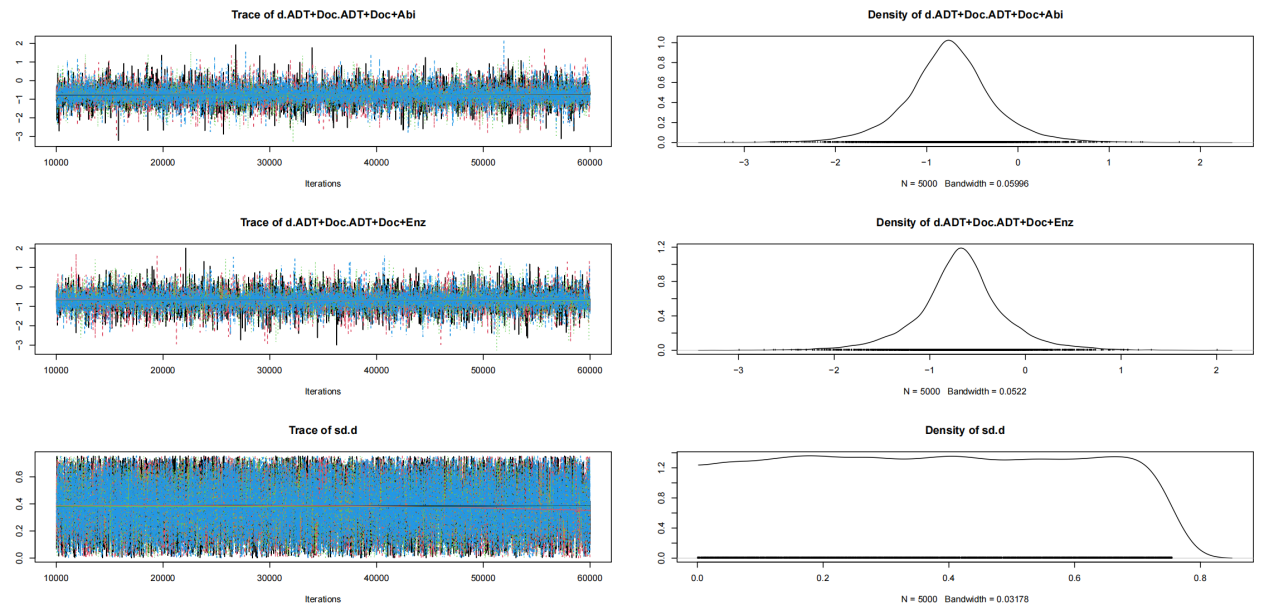


J：Trace and density plots for generalized PFS (LV patients with ADT + Doc + ARSi concomitant)


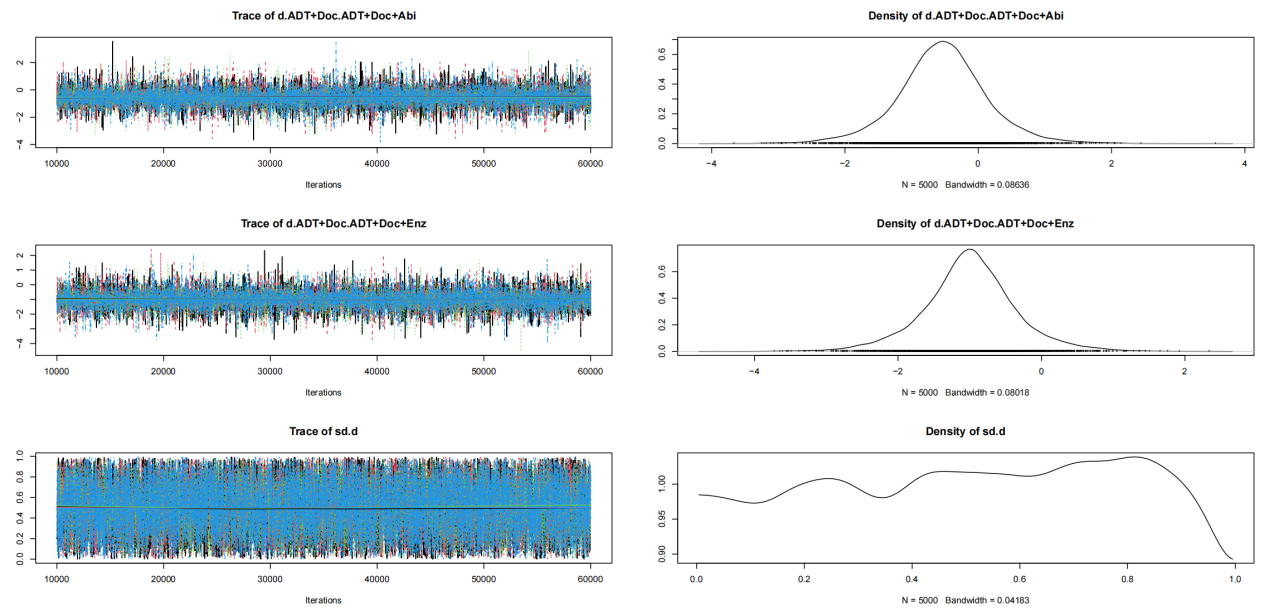


K：Trace and density plots for PSA


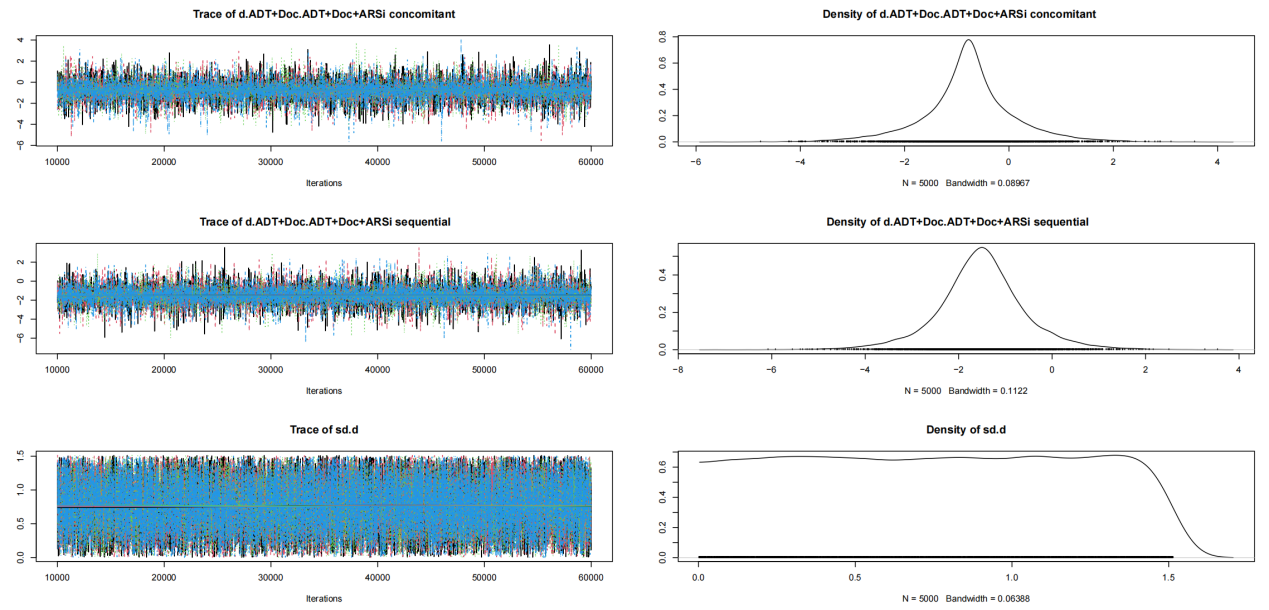


L：Trace and density plots for SAE


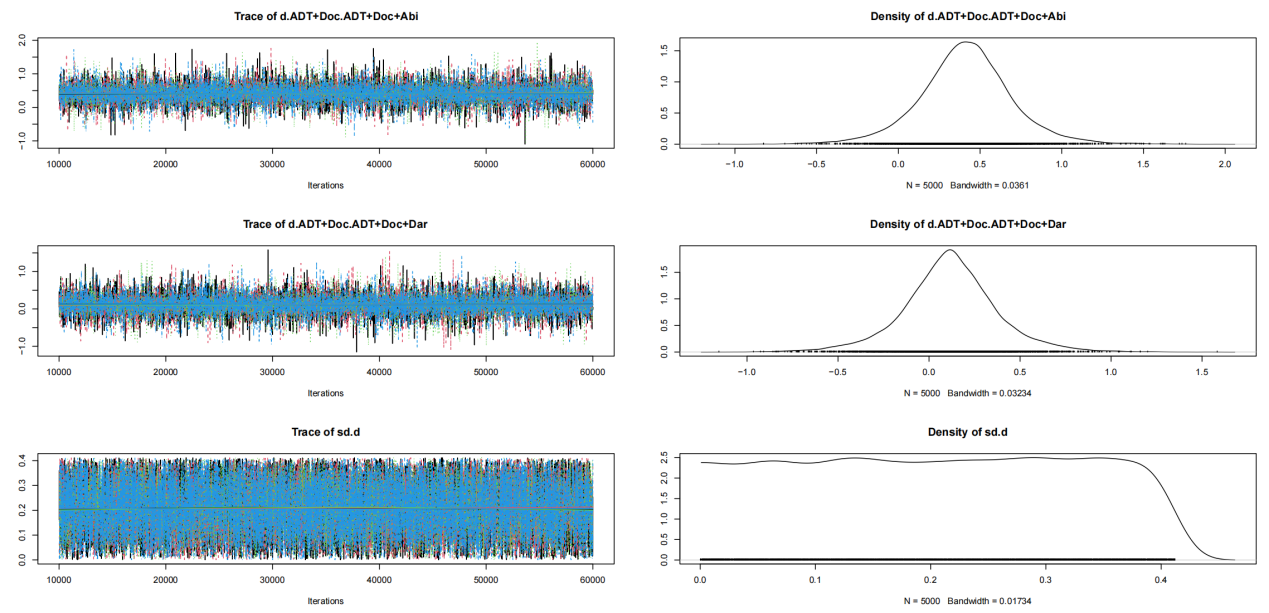


**Supplementary Material 11： SUCRA plots**

OS：overall survival；generalized PFS：generalized progression free survival；rPFS：radiographic progression free survival；PSA：Prostate-Specific Antigen；ADT：androgen deprivation treatment；Doc：docetaxel；Abi：abiraterone；Enz：enzalutamide；Apa：apalutamide；Dar：darolutamide；HV：high volume；LV：low volume；SAE：Serious adverse event.

A：SUCRA plots for OS


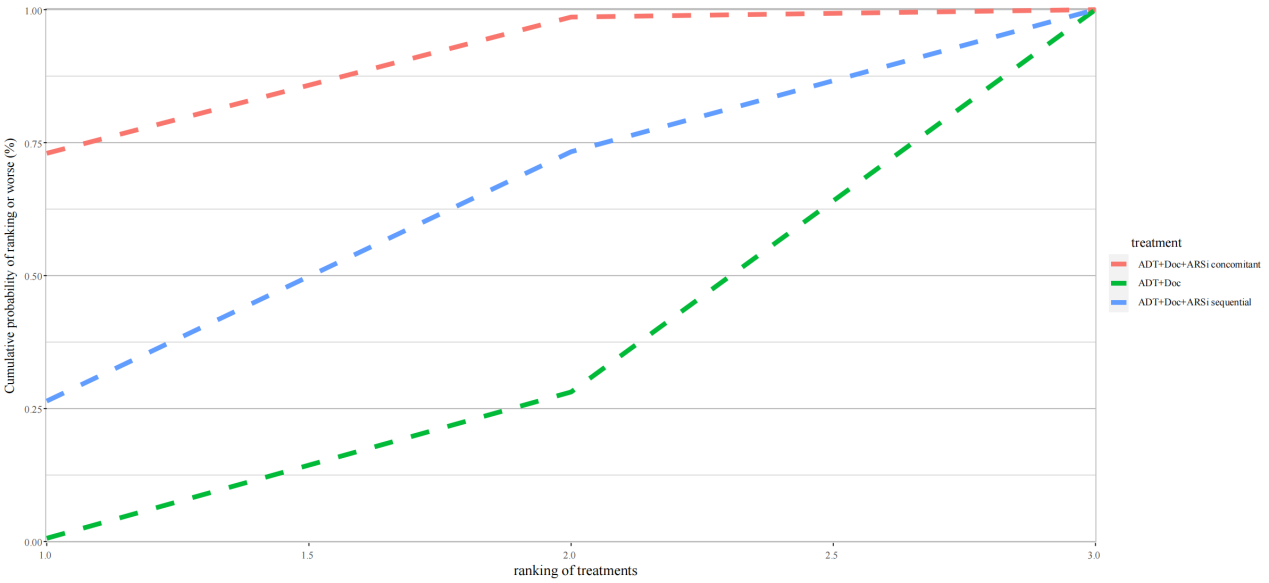


B：SUCRA plots for OS (patients with ADT + Doc + ARSi concomitant subgroup)


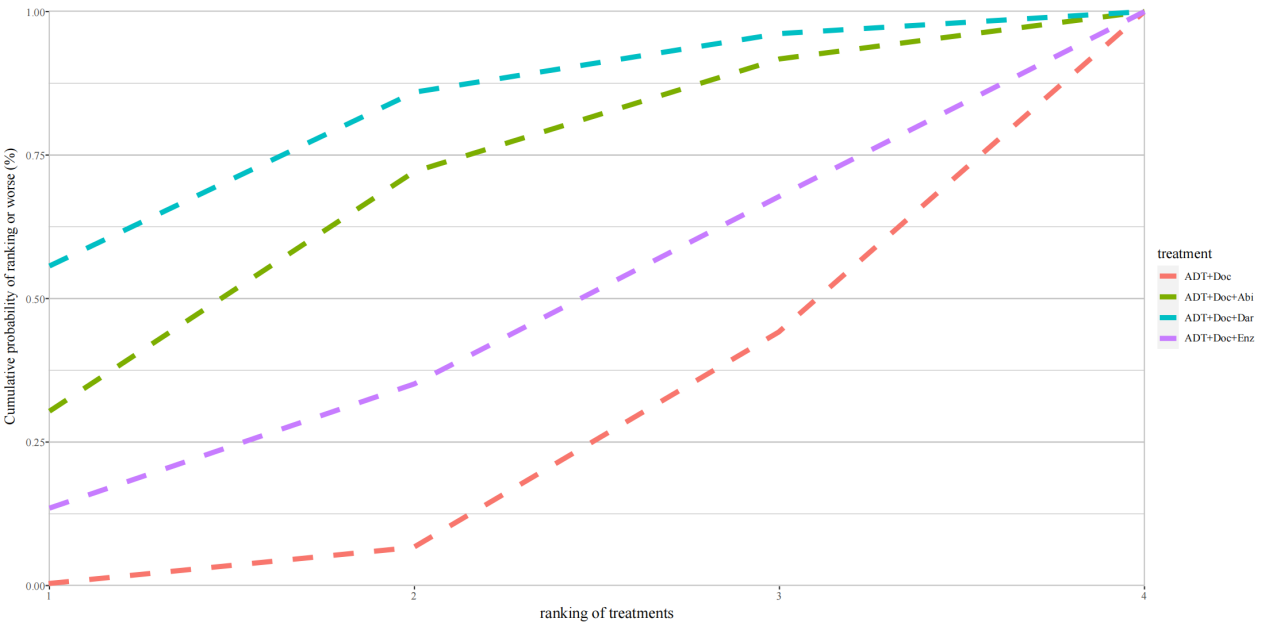


C：SUCRA plots for OS (patients with ADT + Doc + ARSi sequential subgroup)


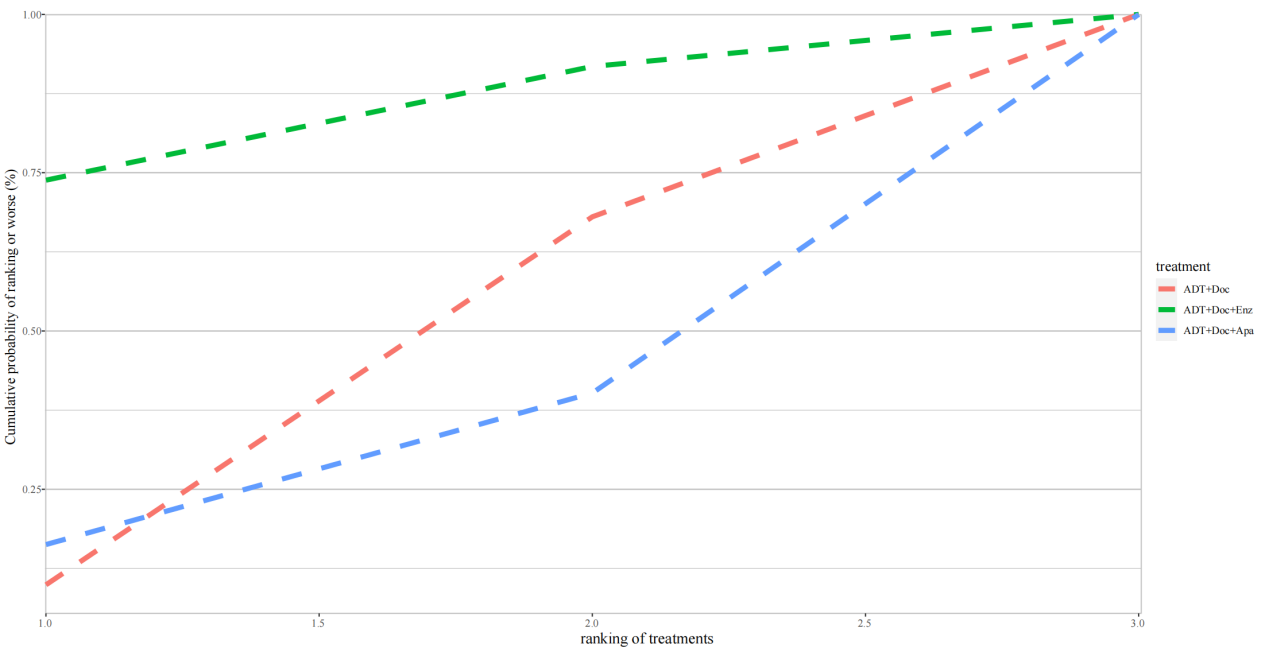


D：SUCRA plots for OS (HV patients with ADT + Doc + ARSi concomitant)


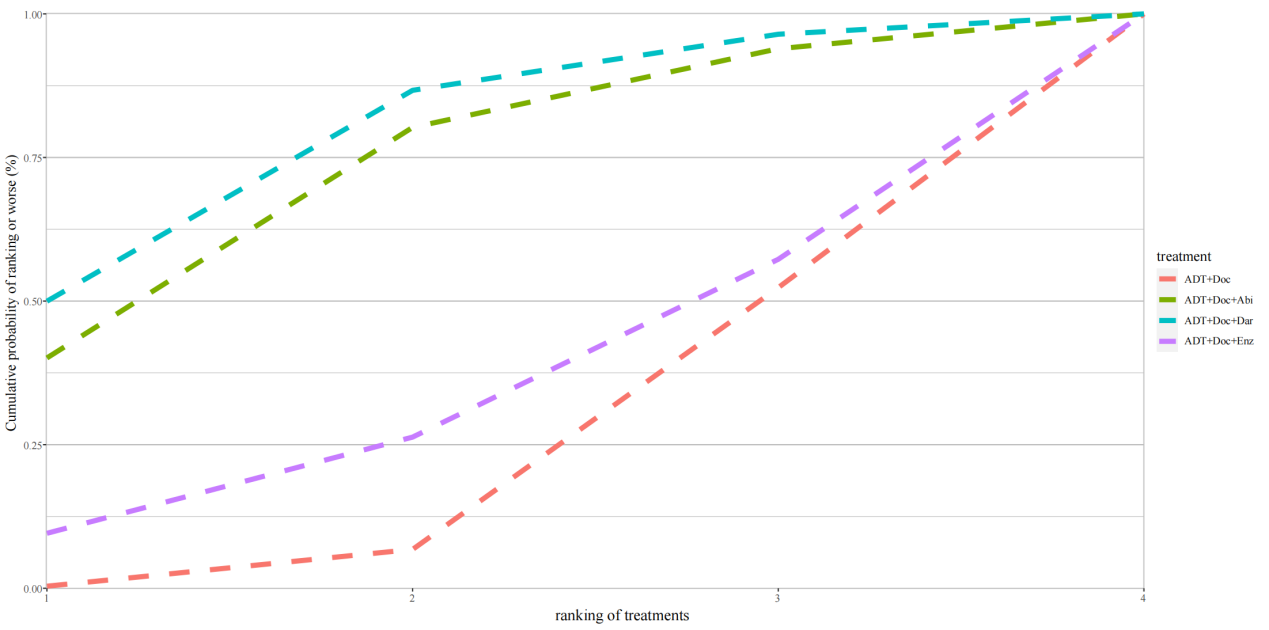


E：SUCRA plots for OS (LV patients with ADT + Doc + ARSi concomitant)


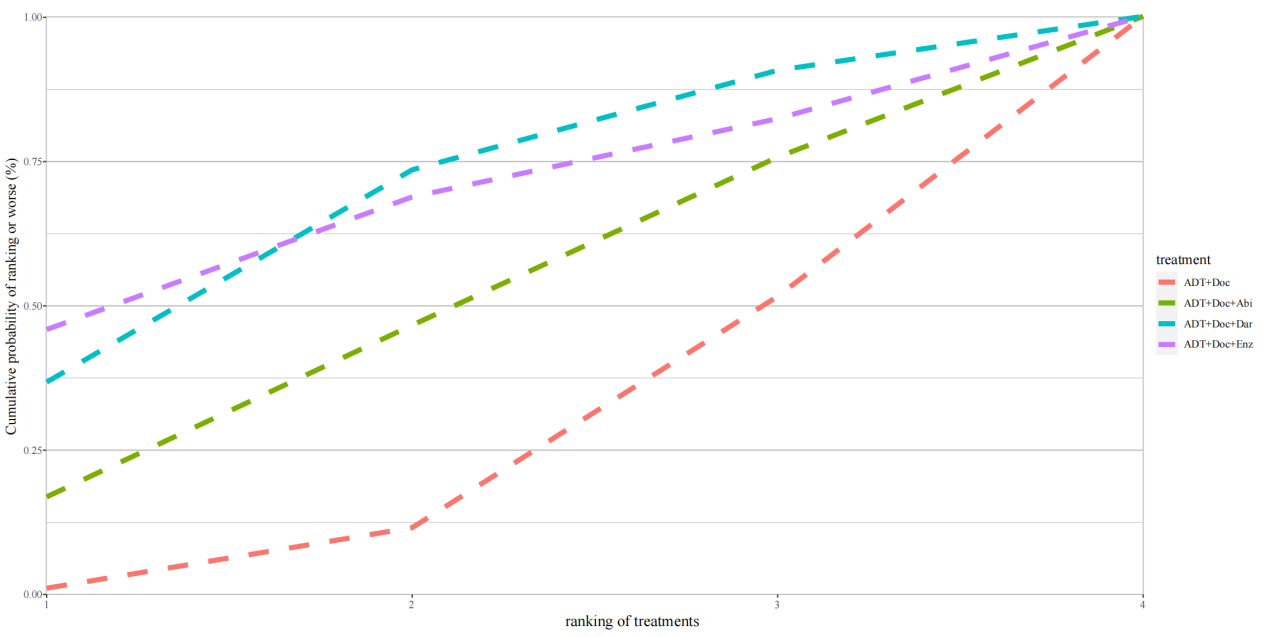


F：SUCRA plots for generalized PFS


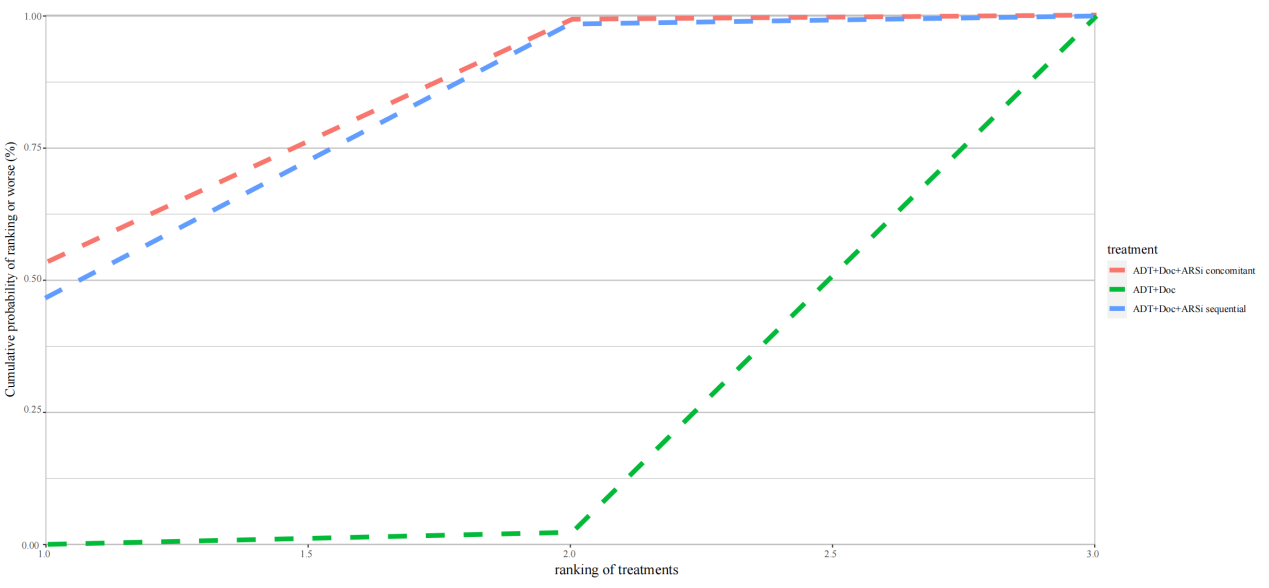


G：SUCRA plots for generalized PFS (patients with ADT + Doc + ARSi concomitant)


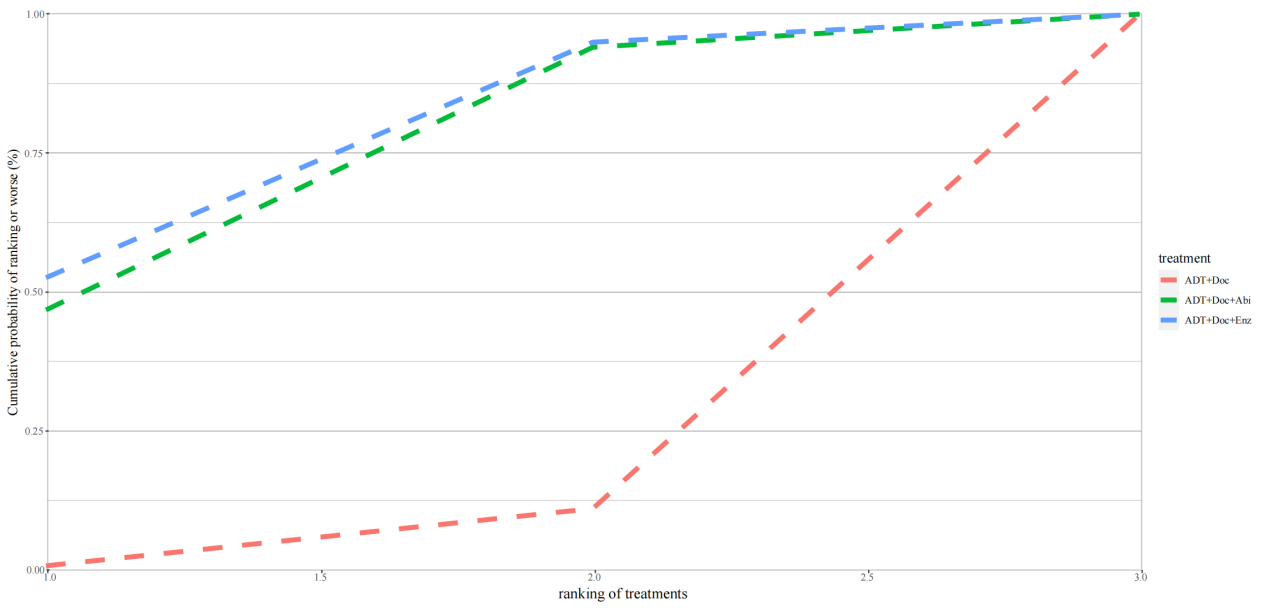


H：SUCRA plots for rPFS (patients with ADT + Doc + ARSi sequential)


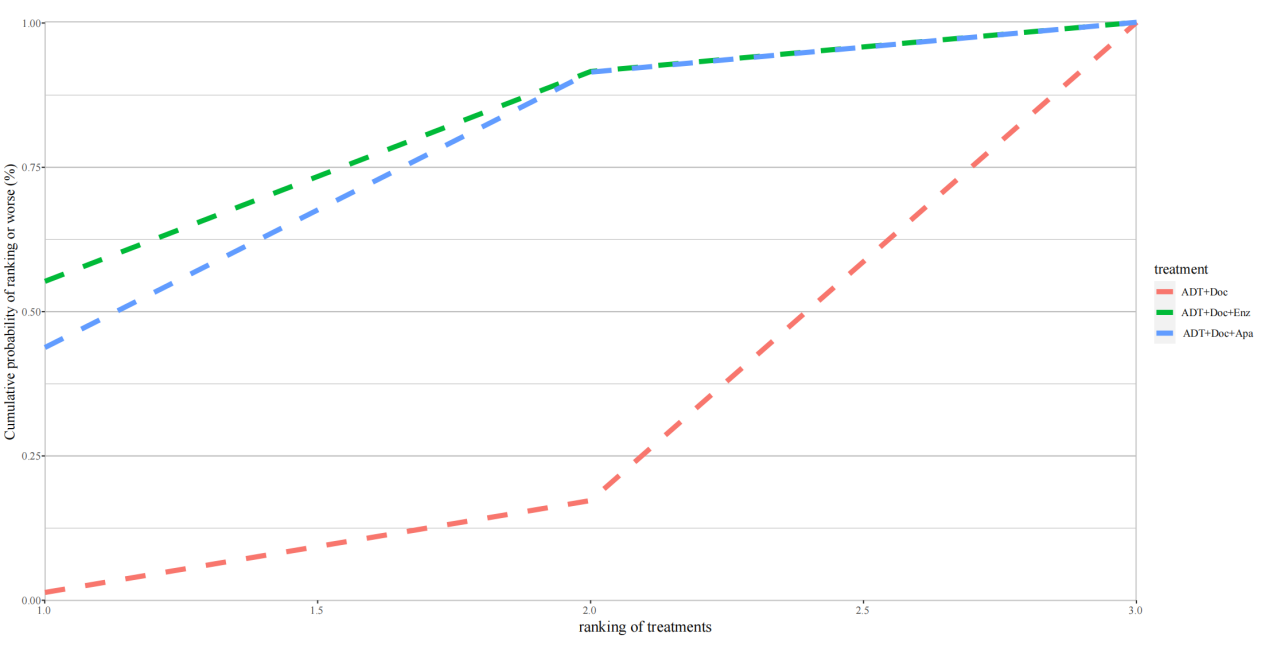


I：SUCRA plots for generalized PFS (HV patients with ADT + Doc + ARSi concomitant)


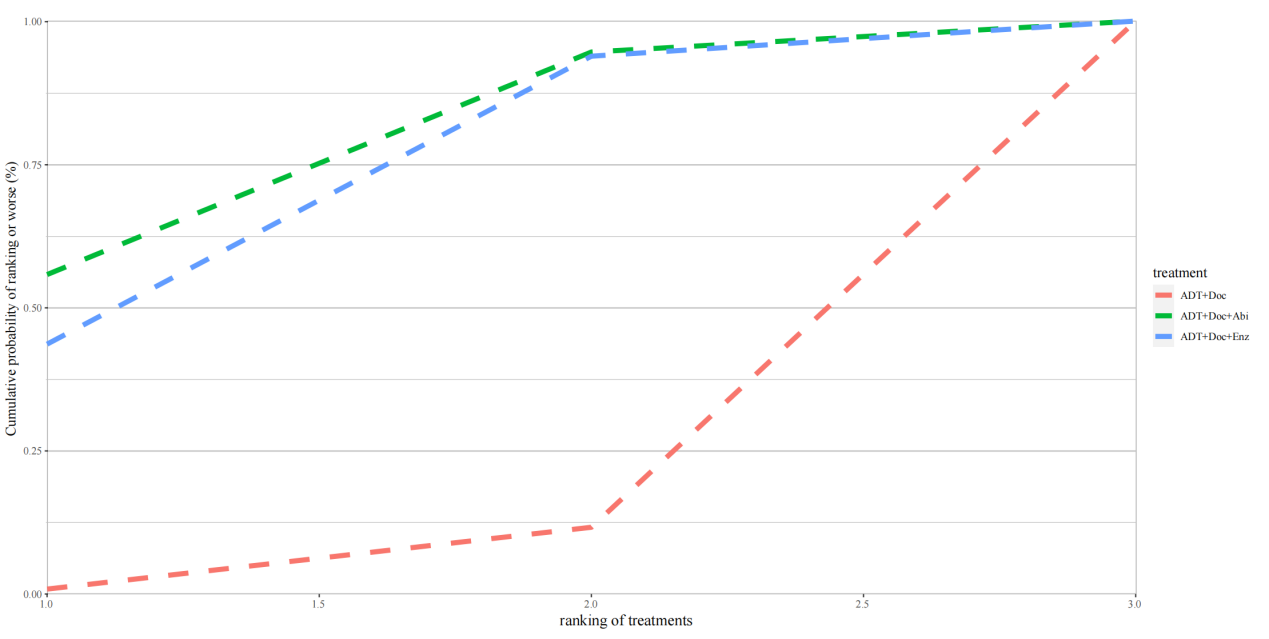


J：SUCRA plots for generalized PFS (LV patients with ADT + Doc + ARSi concomitant)


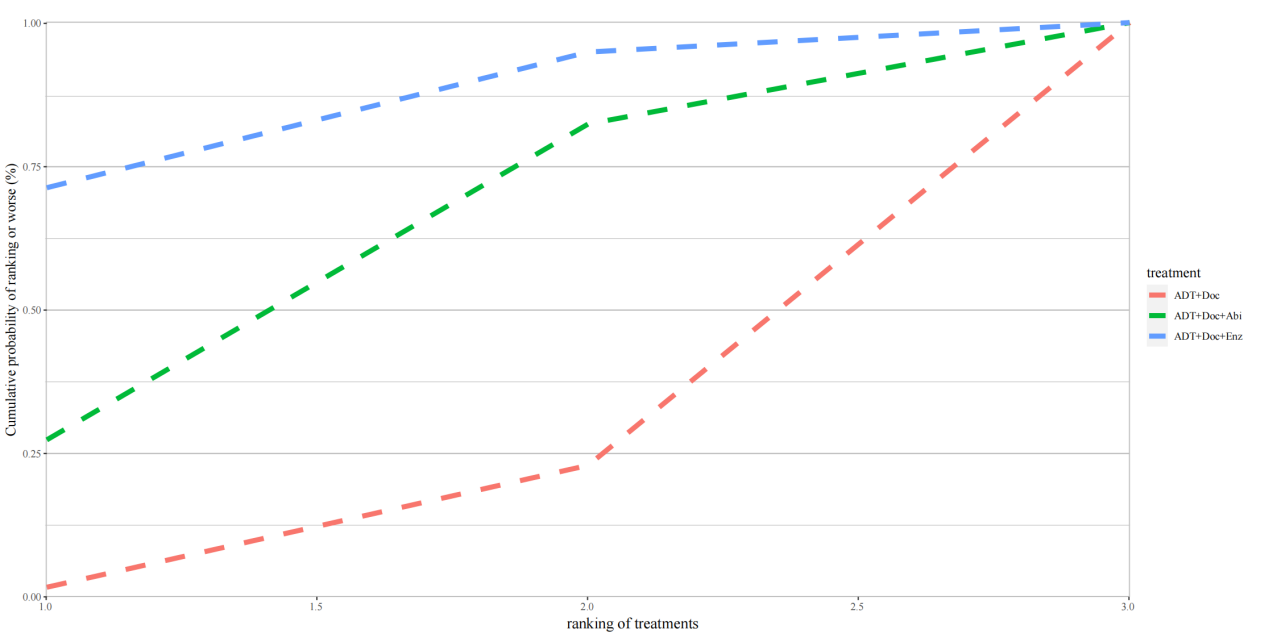


K：Trace and density plots for PSA


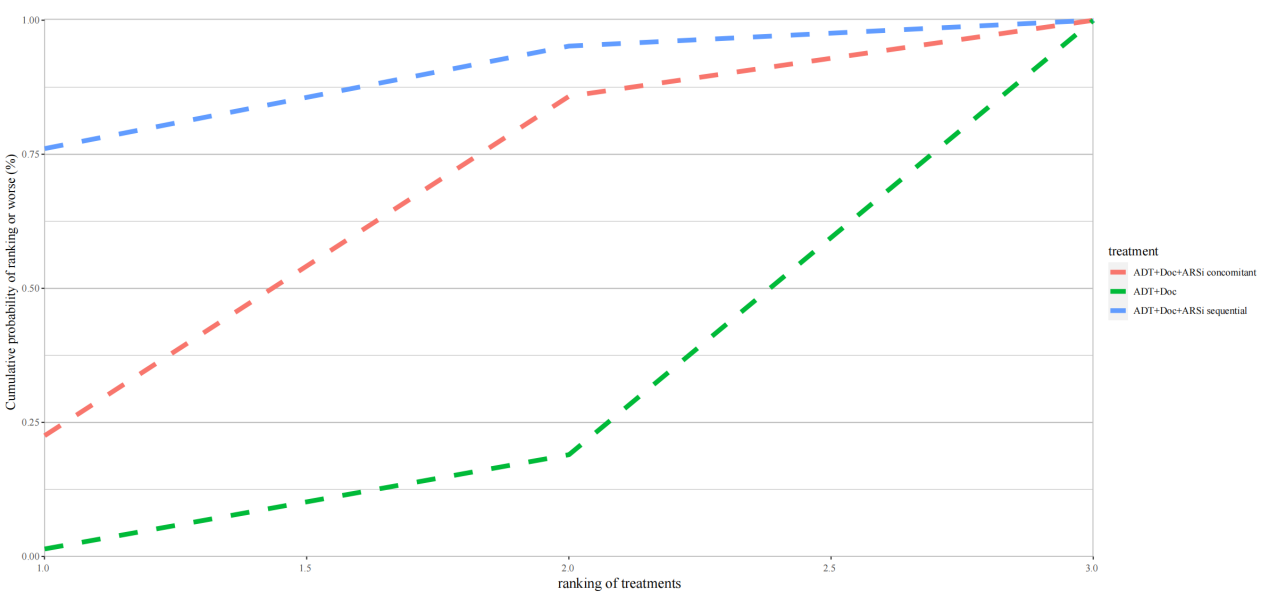


L：Trace and density plots for SAE


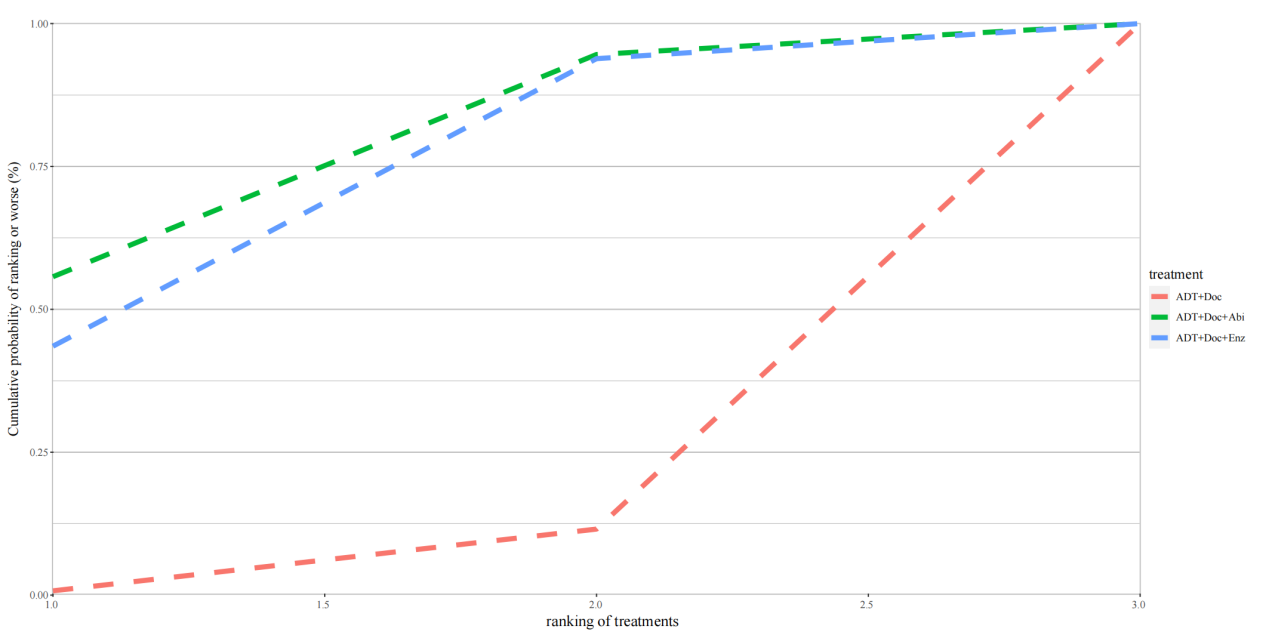


**Supplementary Material 11： BRG plots**

OS：overall survival；generalized PFS：generalized progression free survival；rPFS：radiographic progression free survival；PSA：Prostate-Specific Antigen；ADT：androgen deprivation treatment；Doc：docetaxel；Abi：abiraterone；Enz：enzalutamide；Apa：apalutamide；Dar：darolutamide；HV：high volume；LV：low volume；SAE：Serious adverse event.

A： BRG plots for OS


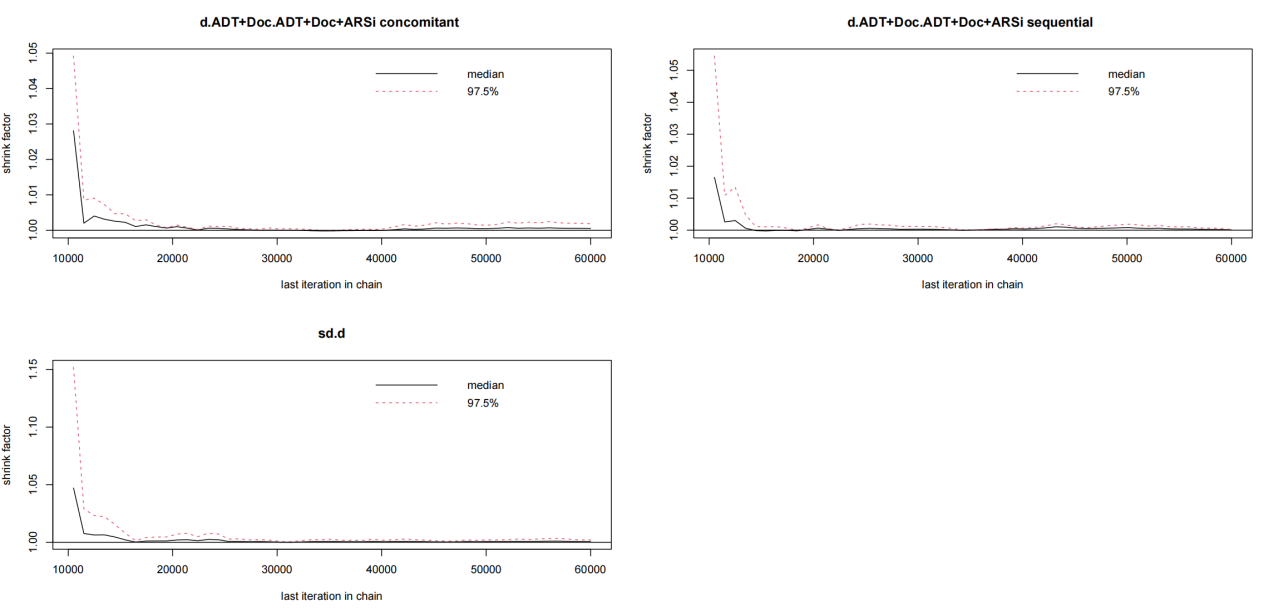


B： BRG plots for OS (patients with ADT + Doc + ARSi concomitant)


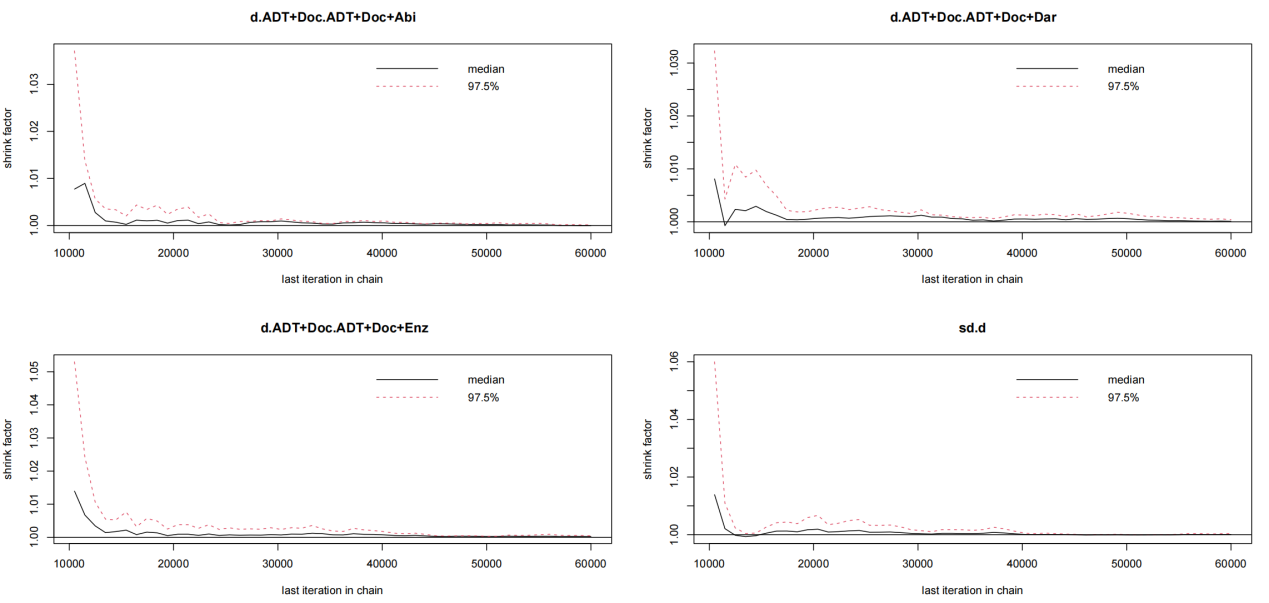


C： BRG plots for OS (patients with ADT + Doc + ARSi sequential)


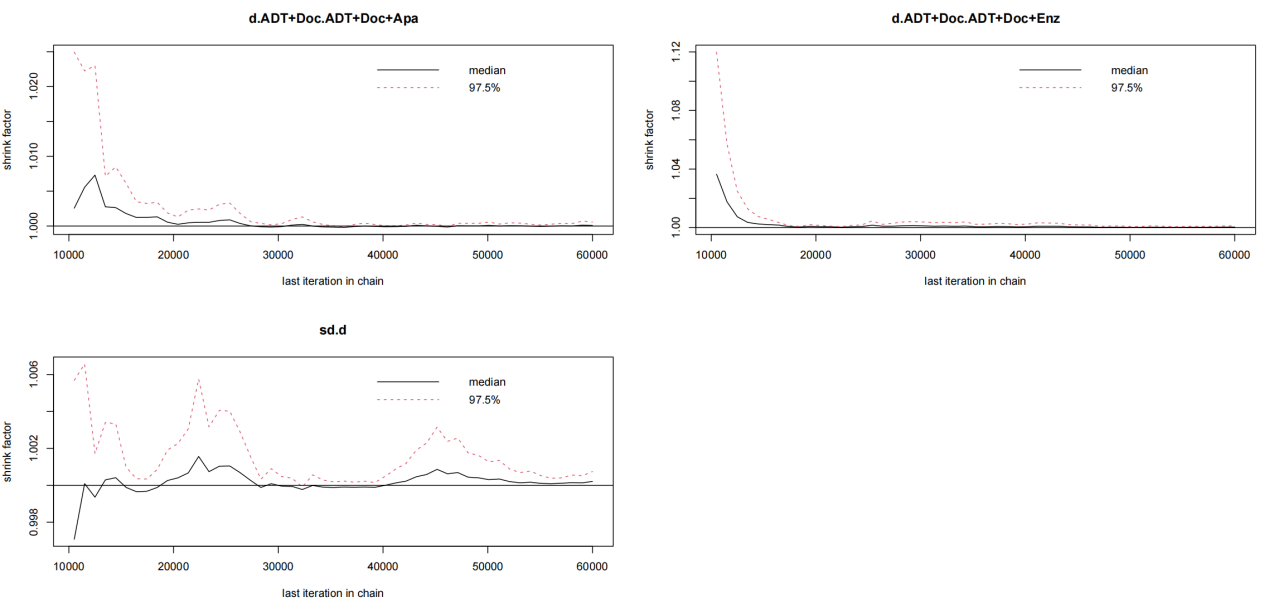


D： BRG plots for OS (HV patients with ADT + Doc + ARSi concomitant)


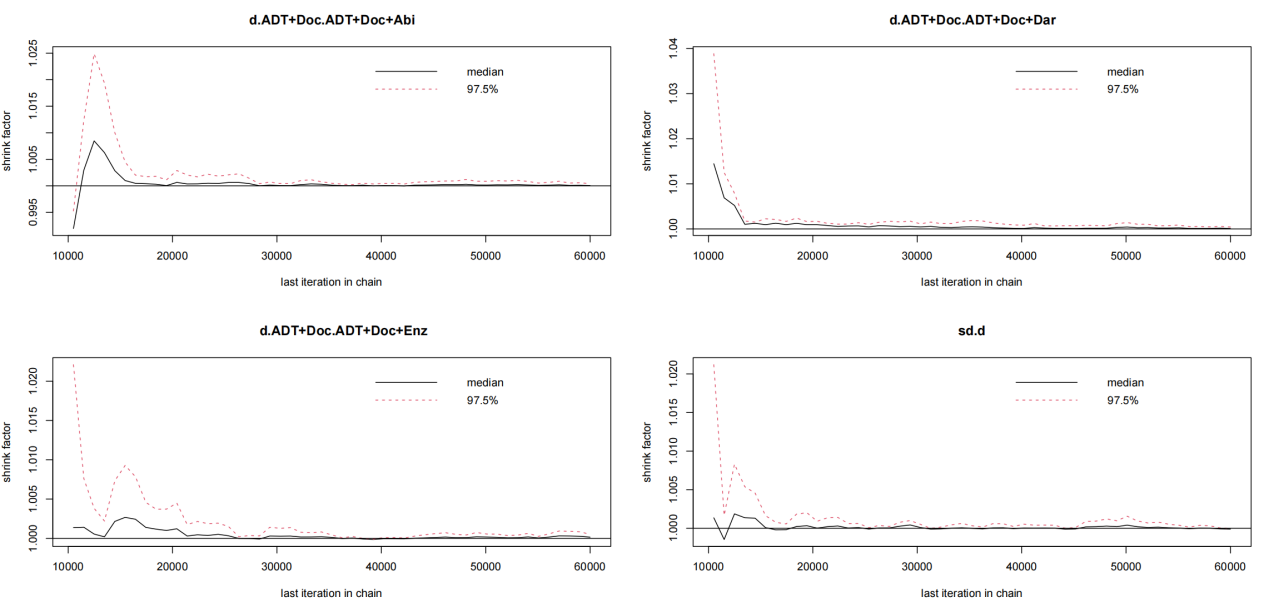


E： BRG plots for OS (LV patients with ADT + Doc + ARSi concomitant)


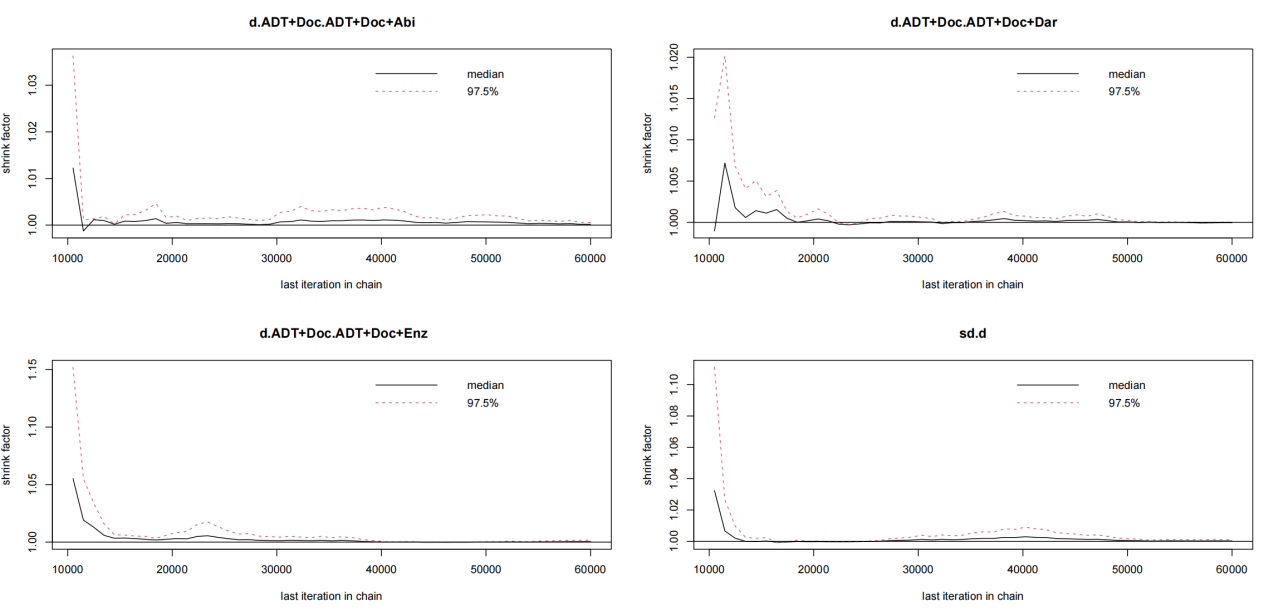


F： BRG plots for generalized PFS


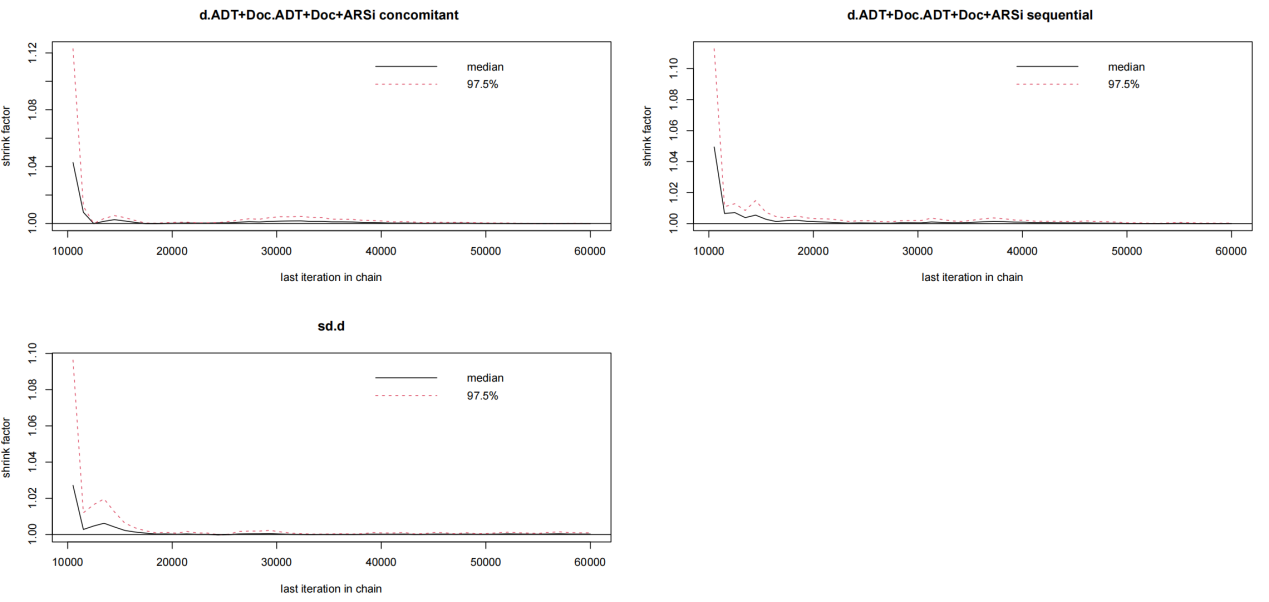


G： BRG plots for generalized PFS (patients with ADT + Doc + ARSi concomitant)


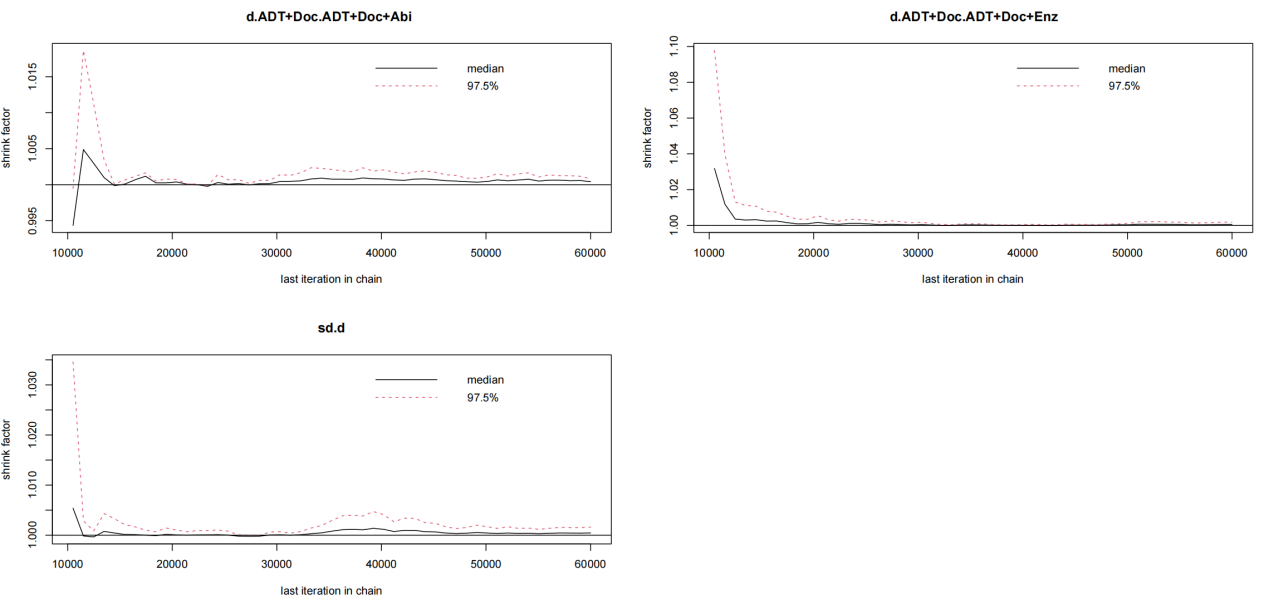


H： BRG plots for rPFS (patients with ADT + Doc + ARSi sequential)


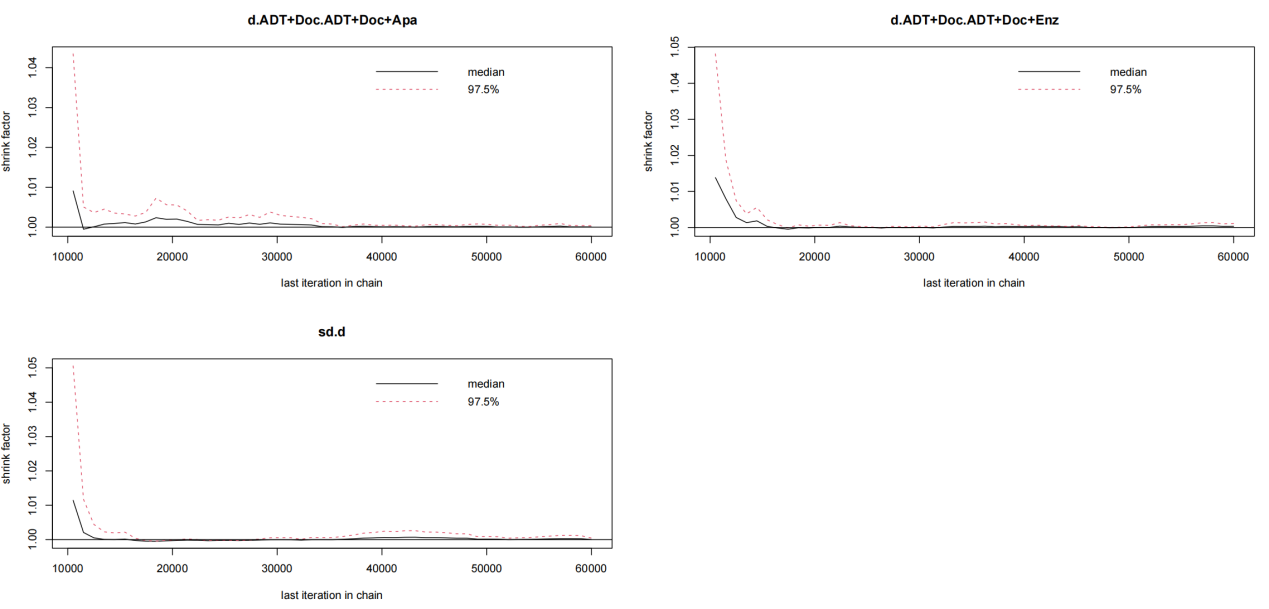


I： BRG plots for generalized PFS (HV patients with ADT + Doc + ARSi concomitant)


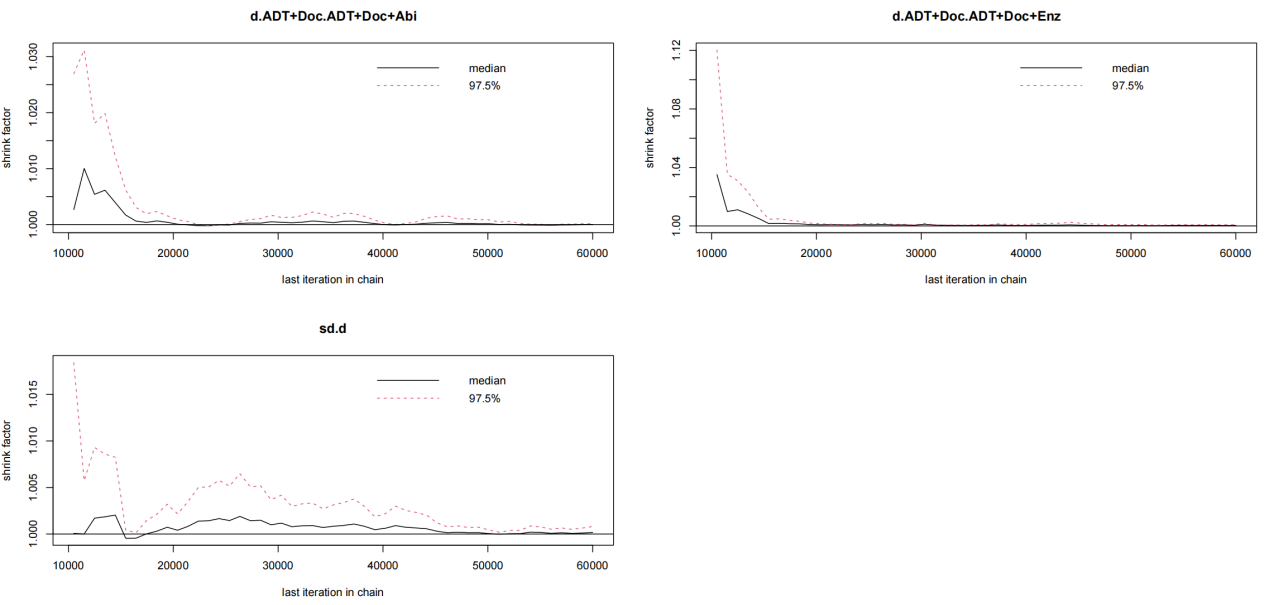


J： BRG plots for generalized PFS (LV patients with ADT + Doc + ARSi concomitant)


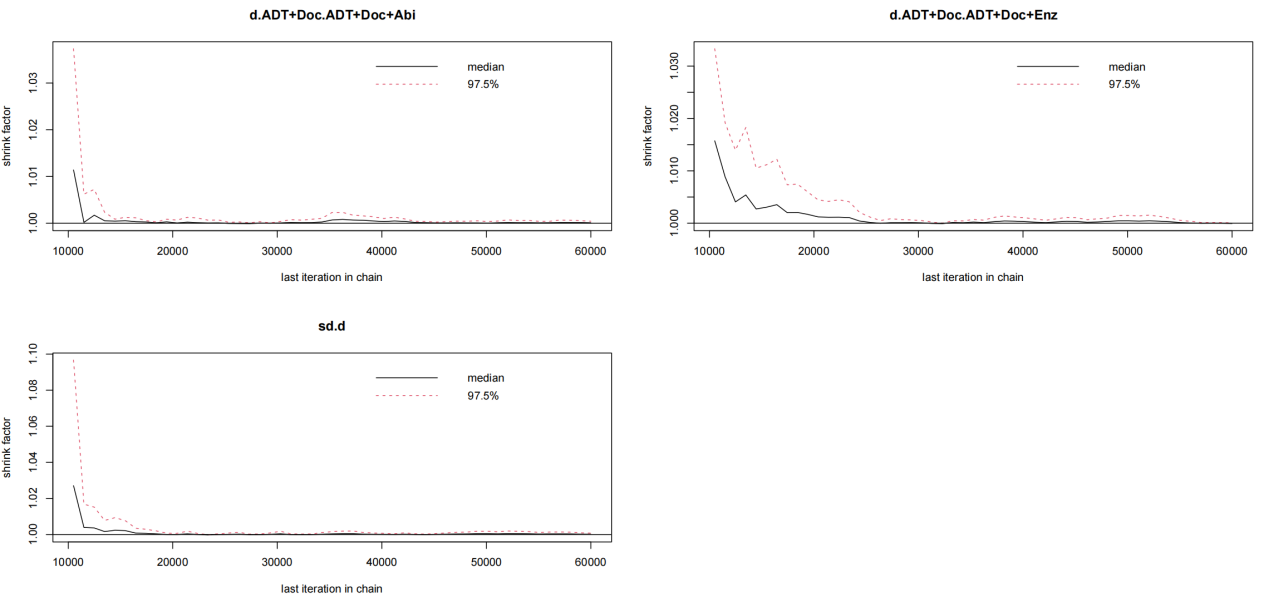


K： BRG plots for PSA


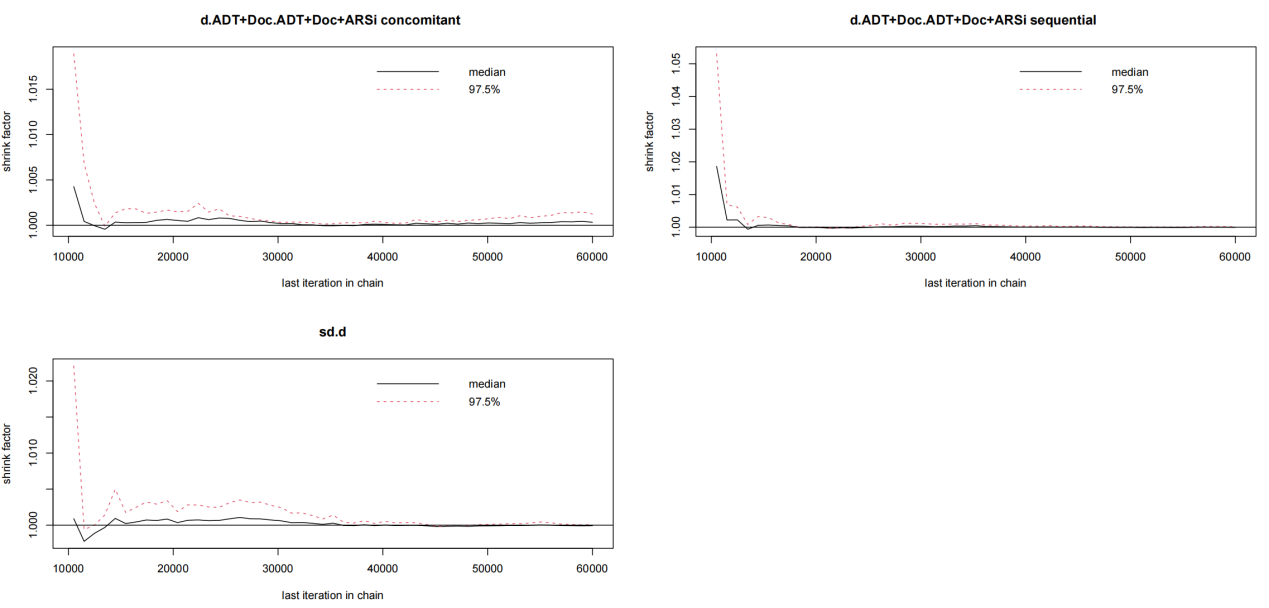


L： BRG plots for SAE


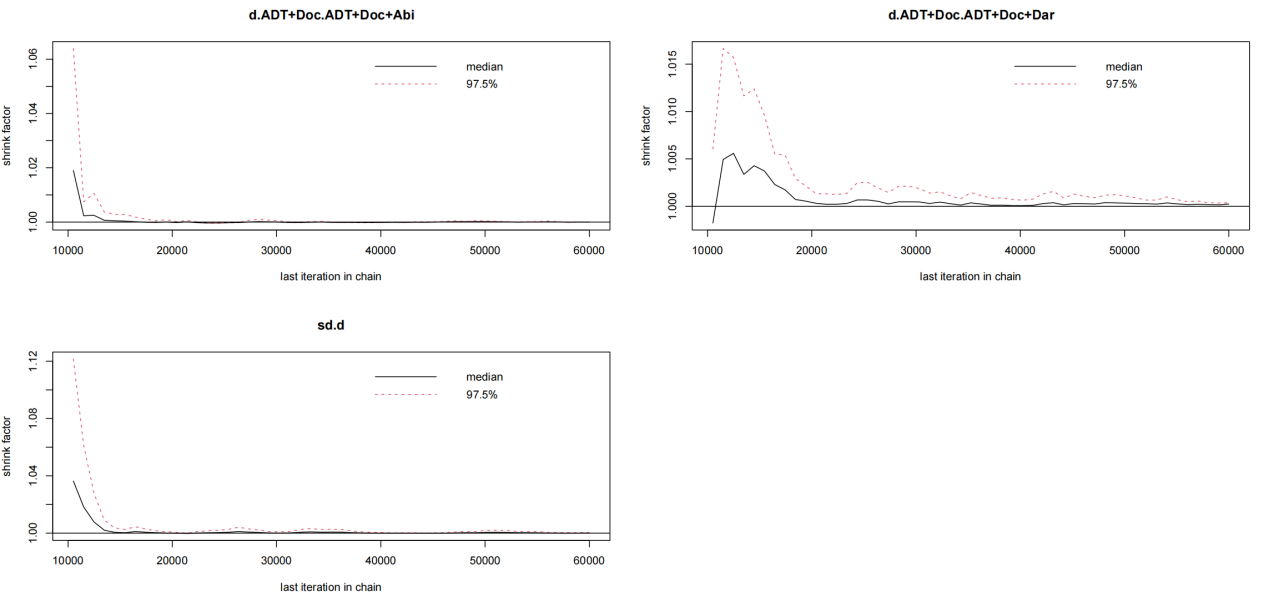


**Supplementary Material 12：AE data**

ADT：androgen deprivation treatment；Doc：docetaxel；Abi：abiraterone；Dar：darolutamide；AE：Adverse Event；SAE：Serious adverse event.

A. 3-5 Grade AE

| Study | Treatment | Grade 3 | | Grade 4 | | Grade 5 | |
| --- | --- | --- | --- | --- | --- | --- | --- |
|  |  | responders | Sample | responders | Sample | responders | Sample |
| ARASENS | ADT + Doc + Dar | 248 | 652 | 183 | 652 | 27 | 652 |
| ARASENS | ADT + Doc | 232 | 650 | 181 | 650 | 26 | 650 |

B. SAE

| Study | Treatment | responders | Sample |
| --- | --- | --- | --- |
| PEACE-1 | ADT + Doc + Abi | 217 | 355 |
| PEACE-1 | ADT + Doc | 181 | 355 |
| ARASENS | ADT + Doc + Dar | 292 | 651 |
| ARASENS | ADT + Doc | 275 | 654 |
